# Visualization and Curve-Parameter Estimation Strategies for Efficient Exploration of Phenotype Microarray Kinetics

**DOI:** 10.1371/journal.pone.0034846

**Published:** 2012-04-20

**Authors:** Lea A. I. Vaas, Johannes Sikorski, Victoria Michael, Markus Göker, Hans-Peter Klenk

**Affiliations:** DSMZ – German Collection for Microorganisms and Cell Cultures, Braunschweig, Germany; Cairo University, Egypt

## Abstract

**Background:**

The Phenotype MicroArray (OmniLog® PM) system is able to simultaneously capture a large number of phenotypes by recording an organism's respiration over time on distinct substrates. This technique targets the object of natural selection itself, the phenotype, whereas previously addressed ‘-omics’ techniques merely study components that finally contribute to it. The recording of respiration over time, however, adds a longitudinal dimension to the data. To optimally exploit this information, it must be extracted from the shapes of the recorded curves and displayed in analogy to conventional growth curves.

**Methodology:**

The free software environment R was explored for both visualizing and fitting of PM respiration curves. Approaches using either a model fit (and commonly applied growth models) or a smoothing spline were evaluated. Their reliability in inferring curve parameters and confidence intervals was compared to the native OmniLog® PM analysis software. We consider the post-processing of the estimated parameters, the optimal classification of curve shapes and the detection of significant differences between them, as well as practically relevant questions such as detecting the impact of cultivation times and the minimum required number of experimental repeats.

**Conclusions:**

We provide a comprehensive framework for data visualization and parameter estimation according to user choices. A flexible graphical representation strategy for displaying the results is proposed, including 95% confidence intervals for the estimated parameters. The spline approach is less prone to irregular curve shapes than fitting any of the considered models or using the native PM software for calculating both point estimates and confidence intervals. These can serve as a starting point for the automated post-processing of PM data, providing much more information than the strict dichotomization into positive and negative reactions. Our results form the basis for a freely available R package for the analysis of PM data.

## Introduction

The so called ‘-omics’ techniques yielded tremendous insights in the biology of cellular organisms. They address different steps in the information transfer from coding DNA (genomics) *via* RNA (transcriptomics) to the proteins (proteomics and interactomics) to finally yield the cellular metabolites (metabolomics and fluxomics) [1]–[3]. Other ‘-omics’ techniques are MicroRNomics, probiogenomics, lipidomics and fluxomics [4]–[7]. Their unifying theme is the study of the cellular totality of the organisms of interest to obtain a systematic insight into basic biology [8]–[9] and to reconstruct complex metabolic networks and flow-charts of fluxes [10]–[13]. The data flood to be processed is enormous, depending on the experimental setup.

A major biological feature, the phenotype, was until recently not accessible with high-throughput techniques. This is unfortunate, as it is the phenotype which is the object of selection and, hence, is the level at which evolutionary directions are governed [14]. All previously addressed ‘-omics’ techniques merely study components which finally contribute to the phenotype [15].

In microbiology, a simple way to assess the phenotype is to characterize an organism's replication behavior under specific conditions [16]–[17] by analyzing the shape of the growth curve during the commonly known growth phases. The length of the lag phase reveals how fast and well the organism acclimates to a specific environmental condition, while the period of cell replication, the log phase, and the stationary phase (when growth comes to an end) indicate the particular way the growth is achieved [18]. Unfortunately, manually recording growth curves is an extremely time- and cost-intensive work.

The Phenotype MicroArray (PM) system appears to close the gap of capturing a large number of phenotypes in high-throughput systems. In this approach, a physiological reaction producing NADH engenders a redox potential and flow of electrons to reduce a tetrazolium dye [19] such as tetrazolium violet, thereby producing purple color. The more rapid this metabolic flow, i.e. cellular respiration, the faster the formation of purple color [20]–[21]. The OmniLog® PM system records the color change every 15 minutes in an automated setting under up to 2000 distinct physiological challenges, such as the metabolism of single carbon sources, metabolism under varying osmolyte concentrations, and response to varying growth-inhibitory substances [20]–[21]. The challenges can be further augmented by modifying environmental conditions such as the temperature and the composition of the gaseous phase.

In common ‘-omics’ techniques, the recorded value is a mostly qualitative information on the difference between two experiments, usually obtained from measurements at a single time point, which is often an endpoint [22]. In contrast, the PM respiration kinetics add a longitudinal dimension. This higher level of PM data complexity contains additional valuable biological information coded in the shape characteristics of the recorded curves in analogy to conventional growth curves as introduced above [18]. These curve features can, in principle, unravel fundamental differences or similarities in the respiration behavior of distinct organisms, which cannot be identified by endpoint measurements alone.

This wealth of data was till now hardly exploited, as the kinetics were usually only qualitatively assessed [23]–[27]. The mere classification into a positive or negative reaction to an environmental challenge appeared to be sufficient, whereas the kinetic information itself was neglected. Also, the application of PM in functional genomics, as, e.g., for improving genome annotation [28] and assessing gene function using knock-out techniques, exploits only presence/absence calls [29]. Nevertheless, already these early studies exhibited the complexity of the situation by, in the light of current knowledge, completely unexpected or even incomprehensible results [30]. Even though the need for a more sophisticated strategy for data analysis was emphasized long ago [31], only data recording could be accelerated until now. Although first attempts to establish web-based data storage and analysis infrastructures were already made [32], an efficient bioinformatic evaluation tool that includes all steps of longitudinal-data analysis, or even a methodical collection analogous to BIOCONDUCTOR for conventional microarray data [33], is still unavailable.

The native OmniLog® PM software [34] displays the PM measurements only according to the 8×12-wells plate layout and provides only limited functionality for the visual comparison of kinetic curves, especially if more than two or even numerous curves are compared. The PM software includes a parametric analysis, which calculates parameters describing a curve's kinetic shape but disregards modeling or curve-fitting approaches and does not provide confidence intervals (CIs), even though it is well known that these can be used to examine statistically detectable differences [35]–[36]. Third-party tools include data visualization [37], but to the best of our knowledge are not publicly available. Some simple but effective approaches to data analysis using summary statistics of growth curves [38] or hypothesis-testing frameworks [39] were also published, but these approaches reduce the information content of each curve to one or a few single values and use these to determine respiration differences on the various substrates without considering the curve shapes.

The development of statistical methods for the analysis of longitudinal data started with the pioneering work of Laird and Ware [40] which discussed a general family of models including growth models and repeated-measures models as special cases. Studies on nonlinear and linear mixed-effects models, the integration of splines, random coefficients and variance modeling into a flexible analysis approach based on linear mixed models followed this seminal work [41]–[46]. Highly elaborated tools for the evaluation of longitudinal data are implemented in statistical software such as the packages *drc* [47] and *grofit* [48] in R, *PROC MIXED* in SAS [49], *xtmixed* in Stata [50], and *MIXED* in SPSS [51]. Also, many mathematical models describing growth behavior have been developed [17],[52]–[53].

Most empirical equations such as the logistic law [54] or Richards curves [55] fit well onto growth data *via* plain non-linear regression if the growth follows the typical sigmoid shape, but mathematical simplicity also plays a key role [53]. Hence, the application of these models to even slightly non-typical growth behaviors (e.g., the simple violation of the assumption of symmetry around inflection) can lead to systematic errors [56] and potentially to biologically unreasonable results (see below). To overcome this problem, the best-fitting model can be detected using the Akaike information criterion (AIC), which balances between fit and model simplicity [57]–[58]. Unfortunately, general guidelines for the selection of the types of models to test are unavailable. Spline smoothers [42], [45] are an alternative to describe growth or respiration behavior, particularly if violations of model assumptions are both common and also reveal biologically important information.

Here we explored the free software environment R [59] for both data visualization and fitting of growth curves for the comparative analysis of PM data. R is one of the most widely used solutions for statistical computing, featuring powerful interactive data exploration as well as programming tools and numerous add-on packages. We first assessed the suitability of the *lattice* package [60] for (re-)implementing and comparing previously published [37] and alternative strategies for raw data visualization of 10,944 bacterial respiration curves. Second, we examined which kinds of divergences from typical sigmoid growth curves occur, which kinds of artifacts might affect the reproducibility of the results and, hence, which basic quality-control measures are necessary and can be performed using the here presented software tools. Third, following the model-fitting approach of [61] we assessed the *grofit* package [48] for automatically conducting model fits as well as model-free fits using spline smoothers. The reliability of both approaches when inferring curve parameters (and their CIs) from PM data was compared with the current implementation in the native OmniLog® PM analysis software [34] and the specific merits and deficiencies of either method were determined. Fourth, we applied the tools to research questions relevant for establishing settings for OmniLog® PM production runs, illustrating how the experimenter can detect significance and magnitude of differences between the considered curve parameters to ensure reproducibility of the results in accordance with predefined quality standards [62]. Finally, as another example for the post-processing of the inferred parameters, we classified the curves into characteristic shapes. In contrast to the typical dichotomization of PM curves into occurrence of respiration and lack thereof [27], we here inferred curve archetypes [63] to explicitly address the question of how many, and which, classes of curve shapes optimally represent the data.

Our results enable us to propose software solutions for exploiting multiple respiration kinetics from automated systems such as PM. Since we consider mainly biologically focused users, we believe that the introduction and availability of convenient and reliable data exploration techniques *via* freely available software such as R will allow users of the PM technology to conduct in-depth data analyzes that go significantly beyond the consideration of mere endpoint measurements and presence/absence calls.

## Materials and Methods

### Organisms studied and PM measurements conducted

The first dataset comprised two strains of two species of bacteria (*Escherichia coli* DSM 30083^T^, *E. coli* DSM 18039 = K12, *Pseudomonas aeruginosa* DSM 1707 and *P. aeruginosa* 429SC). *P. aeruginosa* DSM 1707 was grown on M1 agar (5 g/l peptone, 3 g/l meat extract (Oxoid), 15 g/l agar); all other strains were grown on LB medium (lysogeny broth; 10 g/l peptone, 5 g/l yeast extract, 10 g/l NaCl, 15 g/l agar) for nearly 24 h and subsequently measured on GEN III MicroPlates™ (AES Chemunex BLG 1030) in the PM modus over 91 h. Each strain was measured in ten technical replicates. To ensure that all ten replicate plates were inoculated with cells of identical physiological conditions, the desired cell concentration was adjusted in a pool of ten vials of GEN III inoculation medium A (AES Chemunex BLG 72401) which was then simultaneously inoculated into ten GEN III plates. The second dataset followed the same design, but was collected two weeks later, thus representing a biological repetition. The two datasets thus comprised a total of four strains × two biological replicates × ten technical replicates × 96 substrates, hence 7680 individual curves.

To additionally investigate the impact of the age of cultures on the technical and biological reproducibility, the third dataset focused on a single strain only, *E. coli* DSM 18039 = K12, which was grown on solid LB medium for 16.75 h (t_1_), 18.00 h (t_2_), 19.33 (t_3_), 20.50 (t_4_), 21.92 (t_5_), 23.25 h (t_6_), 24.5 h (t_7_), 25.58 h (t_8_) or 40.33 h (t_9_), respectively, and subsequently measured on GEN III MicroPlates™ in the PM modus over 91 hours. For each growth duration age four technical replicates were performed except for t_9_, which was repeated only twice. Dataset 3 thus comprised one strain × eight growth durations × four technical replications × 96 substrates plus (t_9_) one strain × one growth duration × two technical replicates × 96 substrates, hence 3072+192 = 3264 individual curves.

All raw measurements are included in Files S1, S2 and S3.

### Visualization of PM raw data

As the functionality of the native OmniLog® PM software [34] is specialized on only few functions (see above) we first used the add-on package *lattice* [60] for R [59] to visualize the PM curves as heat maps using the function *levelplot()*, equivalent to a re-implementation of the approach of [37]. We then applied *lattice* to explore alternative visualization strategies using trellis graphics, which arrange graphics in a regular grid-like structure. Large and complex structured datasets can be regularly subdivided according to variables from the chosen experimental design, and in each panel one subset can be graphed, finally providing coordinated, high-dimensional views [64]. As curves are the most comprehensive display of kinetics, we used the high-level function *xyplot()*, which can plot curves in any requested sub-division, combination and constellation. We examined which display method provided the most natural way to assess data quality and data integrity. The main potential artifacts, the range of potential curve shapes and other issues potentially affecting measurement reproducibility were identified during this step by visual inspection of all curves.

Plots of all respiration curves are included in File S4, S5 and S6.

### Parameter estimation from respiration curves

For the description of functional dependencies of two measured variables a mathematical function can be fitted onto the data. In general, such a fit aims at minimizing the distances between the raw data points and the values predicted by the function. The choice of a type of function is usually motivated by some basic assumption about the underlying system. The selection of a function is an interpreting activity and a crucial step in the analysis [65]. Alternatively, the dependency between two measured variables can be described by smoothing splines. Those splines can be thought of as a concatenation of cubic polynomial segments that are joined together at their ends or knots [66]. Their unique property as an empirical function is that they can represent any variation in curve shape.

The parametric analysis method of the native OmniLog® PM software [34] only crudely accesses possible differences in curve shapes, because it uses only few data points from the curve for the computation of curve kinetic parameter values (see p. 38 in chapter 5 of the OmniLog® user guide [34]). The maximum height (“MaxHeight”) is given as the 10^th^ percentile highest value among all values over all time points, and the minimum height (“MinHeight”) is calculated as the 12^th^ smallest value among the first 48 reads over all time points. The length of the lag phase is calculated from the raw data using the formula “MidTime - (MidHeight – MinHeight)/Slope” [34], while “MidTime” is described as the first time a value exceeds MidHeight. “MidHeight” is defined as the value midway between MinHeight and MaxHeight. The Slope is calculated as “sum of rises over run between 15% Time and MidTime −1 and rises over run between MidTime +1 and 85% Time” divided by “85% Time minus 15% Time” from the raw data [34]. Here, “x% Time” is defined as the first time a value exceeds the value x% of the way between MinHeight and MaxHeight. The calculation of the area under the curve (AUC) is described as “the sum of all OmniLog values over all time points (area under the curve)” [34], which treats the color changes between time points as a step function. Also, native OmniLog® PM software only provides point estimates but not CIs, which are important for statistical evaluations [35]–[36]. Hence, the software cannot be used to investigate whether two quantitatively similar curves differ in a statistically detectable way.

In contrast, the basic part of R's add-on package *grofit* [48] provides a framework for parameter estimation using model fitting and model-free spline fitting separately and also allows the statistical assessment of the curves using CIs. The model-based approach fits each predetermined model by a non-linear least-squares regression. The Akaike Information Criterion is used to select a best model. The spline-fitting approach is based on a cubic smoothed spline and follows the framework implemented in the R function *smooth.spline()*. We here applied the default smoothing parameter. The package *grofit* [48] was originally built to derive dose-response curves and calculate descriptive pharmacological or toxicological values. For the here proposed application the intermediate output, which contains estimates for curve-describing parameters, is used. Those parameters are the length of the lag phase λ, the growth (here: increase in respiration) rate μ (corresponding to “slope”) and the maximum cell growth (here: respiration) A (corresponding to the maximum value recorded). As an additional descriptive parameter of cell growth (here: respiration), the area under the curve (AUC) is estimated via numerical integration (see the second figure in [48] for details). In the case of the model-based approach the other parameters are directly estimated as parts of the model. The parameter extraction from the fitted splines needs additional steps; here, A is calculated as the maximum value of the fitted spline. The parameter μ (growth rate) is calculated as the maximum slope of the spline, also yielding the corresponding fitted value y_μ_ and the time point t_μ_ of its occurrence. A tangent at this point has the form y(t) = μ(t-λ) and thus yields the length of the lag-phase λ via y_μ_ = μ(t_μ_-λ) (Kschischo, pers. comm.).

In addition to the point estimates for the parameters from both model and spline, confidence limits can be calculated *via* bootstrapping, with 95% being the default value [67]. Significant differences can then be detected as non-overlapping CIs. In case of no overlap, the differences between the opposite limits of the considered CIs describe the smallest expectable mean difference.

We assessed in detail in how many (and which) cases a model fit was impossible using one of the default models: (i) logistic growth, (ii) Gompertz growth, (iii) modified Gompertz growth and (iv) Richards growth [48]. Particular emphasis was laid on biologically unreasonable parameter estimates as observed in preliminary experiments (data not shown) such as negative values for λ and estimates for the A exceeding 400 OmniLog® units (due to technical restrictions, the current version of the OmniLog® device yields measurements at most 400 OmniLog® units in height; Bochner, pers. comm.). To provide a rough estimate for the proportion of positive and negative reactions, we applied a (partially arbitrary) threshold of 100 OmniLog® units, i.e. larger estimates of A indicated positive reactions, other values indicated negative reactions (for a more advanced treatment see the inference of archetypes below). We also determined the correlations between all four parameters from the same curve fitting as well as between those from model fitting and spline fitting applied to the same raw data. Spearman's correlation index and Kendall's τ were compared, since the data are not necessarily normally distributed and the relationships not necessarily linear.

The accuracy with which the parameters (estimated using the three different approaches) fitted to the raw data was investigated for all types of observed curve shapes (see above) and visualized using a set of individual curves representative for each shape. These exemplars could also be used to illustrate the difference in parameter estimation between model and spline fit and thus for the identification and explanation of the effect of difficult-to-fit curve shapes on parameter estimates. Moreover, they were used to determine the most useful way of displaying parameters estimated together with their CIs. The proposed methods here intentionally resign any multiplicity adjustment, because the analyses are expected to detect all interesting phenomena while it would be worse to miss some of them.

### Detecting significant differences

Because there is no restriction on the type of sample to be analyzed, the PM technique is capable of dealing with a rather unlimited amount of distinct experimental questions. That is, not only isolated strains or well-defined mutants are manageable, but also mixed or environmental samples are feasible [68]–[69]. For most of them predictions about their behavior are impossible, thus the experimenter needs to compare repeated measurements to be able to assess the range of variability in the specific sample, strain, etc. Depending on the experimental design, the usual sources of variations, namely variation between technical repetitions, between biological repetitions and between experimental repetitions etc., occur and contribute to the total variation of each curve or set of curves. To demonstrate the value of CIs for data evaluation, we assessed scenarios where (i) curves differ significantly in general, (ii) replications differ significantly in some parameters but not in others, and (iii) differences between replications are not statistically detectable, as indicated by the 95% CIs. Such exemplars were also used to determine efficient ways to display these differences. As a laboratory example, we calculate 95% CIs from the third dataset to assess whether there was a significant impact of the age of the bacterial inoculation culture on technical and biological reproducibility. That is, the repetitions measured after distinct durations of cultivation need to be compared against each other because, if such a dependency was detected in a real-world dataset, the experimenter would need to more strictly standardize cultivation times prior to conducting PM measurements.

Since up to now the *grofit* package is not intended for fitting a single model or spline on a set of several repetitions of a longitudinal data set, we present two alternative approaches for their comparison. First, we provide a graphical solution which yields preliminary insights into the overall behavior of the considered groups and is based on mean parameter estimators and mean CIs calculated by averaging the corresponding values estimated from the individual curves. Second, as a somewhat more sophisticated approach, we provide a simultaneous multiple comparison procedure of means [70]. It provides test decisions using 95% CIs for the differences of parameter means according to a user-defined set of comparisons.

Parameter estimates from all respiration curves including CI limits are available in Files S7 and S8. The behavior of the negative controls (well A01) was examined more closely, particularly regarding the question whether it is valid to subtract these values from the measurements from all other wells before estimating curve parameters, a procedure which is sometimes recommended [34]. Apparently, this strategy assumes a biologically sensible additivity between the negative control and respiration reactions caused by the substrates. Our results are presented in File S9.

### Which, and how many, classes of curve shapes?

To explicitly infer the optimal number of distinct shape classes for classifying the curves, we applied the R package *archetypes* [63] to the parameter estimates from the spline fits from the 1^st^ and 2^nd^ dataset. Archetypes are characteristic extreme types of combinations of multivariate observations found by minimizing a convex residual sum of squares (RSS) criterion; the implementation ensures that the real measurements can be represented by convex combinations of the archetypes. The algorithm alternates between finding the best set of coefficients for the given archetypes and finding the best archetypes for a given set of coefficients. The overall RSS is reduced successively because in each step several convex least-squares problems are solved. While the number of archetypes is predefined in each run, the algorithm can be restarted for a series of numbers of archetypes (we tested 1 to 10) and, according to the “elbow criterion”, the optimal number is the one that resulted in the largest step towards a lower RSS compared to subsequent improvements. We used the *stepArchetypes()* function with five random starts per given number of archetypes. As some of the spline estimates for the parameter λ were outliers below zero (see below), its distribution was truncated (made symmetrical) by setting all values lower than the maximum times −1 to this value.

## Results

### Visualization of PM raw data

**Figure 1:**
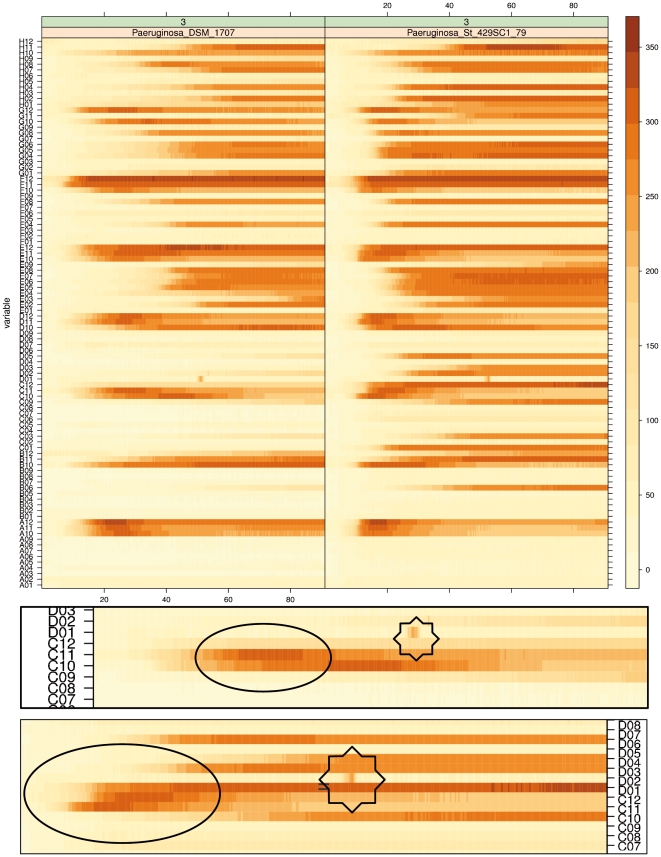
Visualization of PM curves as heat maps *via* the function *levelplot()* as a re-implementation of the approach of Jacobsen et al. (2007) in R. Each respiration curve is displayed as a thin horizontal line, in which the curve height as measured in OmniLog® units is represented by color intensity (darker parts indicate higher values). The x-axes correspond to the measurement time in hours. The upper part shows an overview of two plates. Here, the descriptions of the y axes (only visible if enlarged, but see below) list the names of the wells; the descriptions of the x axes list the measurement time in hours. The boxes below represent magnified parts of the upper panel to illustrate the color changes in the case of decreasing color intensities (regions surrounded by black ellipses) or technical problems such as short-term intermediate peaks (positions marked by eight-pointed stars).

Using a subset of dataset 1 the visualization of PM curves as heat maps *via* the *lattice* function *levelplot()* is shown in Fig. 1. The user was free to define any ordering of the lines in the columns, since the well position on the 8 × 12 GEN III MicroPlate™ is given on the y-axis and identification was easily possible. This also allowed the comparison of technical and/or biological replicates after an appropriate re-arrangement (data not shown). One advantage of this visualization technique was that numerous curves could be displayed in relatively small space, when vertical lines representing the respiration curves were stacked (Fig. 1). Further data quality assessment was feasible straightforwardly; for instance, deviations from the expected monotonic increase of the curve height could be identified and located (Fig. 1).

**Figure 2:**
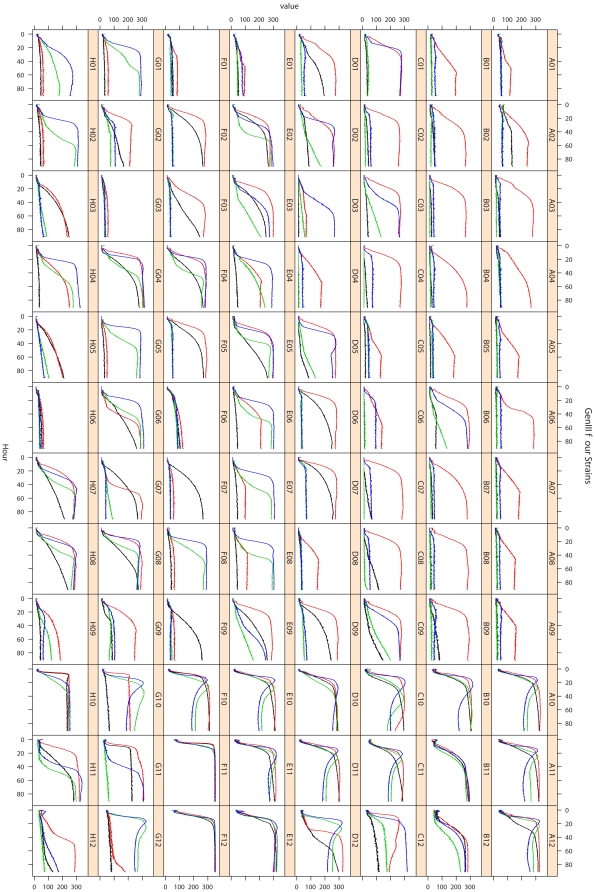
Visualization of PM curves as such *via* the function *xyplot()*. PM curves from a representative technical repetition from the first dataset were arranged according to the original 8×12 wells plate layout. The respective curves from all four strains are superimposed; the affiliation to each strain is indicated by color as follows: black, *E. coli* DSM 18039; red, *E. coli* DSM 30038^T^; green, *P. aeruginosa* DSM 1707; blue, *P. aeruginosa* 429SC. The x-axes show the measurement times in hours, the y-axes the curve heights in OmniLog® units. In the caption of each panel the corresponding coordinate of the well is shown. Details of the curves from wells G11 and H11 are examined in Figs. 3 and 4.

In Fig. 2 kinetic data are plotted according the original 8×12 well layout as superimposed curves by using the function *xyplot()*. Here, the user was also free to rearrange distinct numbers of curves in individual compositions of panels. Distinct organisms or treatments could be color-coded (Fig. 2) or alternatively indicated by distinct line types (not shown). Such a graphical display easily enabled to monitor the general performance of an organism and to simultaneously identify potential artifacts, such as individual replications that deviate in curve height or shape, or other irregularities such as deviations from the expected monotonic increase of the curves, particularly when superimposing the curves from distinct replicates of the same reaction in one panel.

**Figure 3:**
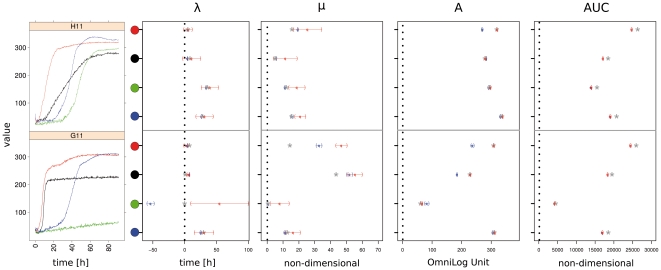
Comparison of parameter and CI estimates from the same curves using three distinct approaches. Left, enlarged view of the curves from wells G11 and H11 as depicted in Fig. 2. As in Fig. 2, the affiliation to each strain is indicated by color as follows: black, *E. coli* DSM 18039; red, *E. coli* DSM 30038^T^; green, *P. aeruginosa* DSM 1707; blue, *P. aeruginosa* 429SC. Right, point estimates and 95% CIs for each of the four parameters lag phase (λ), slope (µ), maximum (A) and area under the curve (AUC) estimated from the eight curves depicted on the left using either the model-fitting (blue dots and CIs) or the spline approach (red dots and CIs). The gray stars are the respective point estimators inferred with the native OmniLog® software (which does not provide CIs). The colored circular areas refer to the colors of the curves in the left part of the figure.

We felt that data quality and integrity could be checked faster and more comprehensively using the second, curve-based visualization approach. The curve display gave a more intuitive and straightforward overview of the data, while simultaneously facilitating the development of an overall assessment of an organism's behavior in the experiment. Moreover, color codes for results from distinct organisms, replications and experiments enabled informative superimposed displays (Fig. 2), which would be difficult when color is used to indicate signal strength (Fig. 1). For this reason, we used the visualization approach of Fig. 2 to inspect the curves from all PM experiments. By this, we found various combinations of negative reactions, where (nearly) no color was formed in the wells, and positive reactions per organisms and/or experiment, particularly between the distinct biological replicates (see File S4, S5 and S6). It also turned out that a surprisingly large number of curves from positive reactions diverged from the typical sigmoid shape of growth curves (Figs. 2, 3). In most sets of technical and/or biological replicates which included such deviating curve shapes, these occurred in all of the respective replicates (see Files S4 and S5).

### Parameter estimation from respiration curves

We estimated the parameters length of the lag phase λ, respiration rate μ (slope), maximum cell respiration A and the area under the curve (AUC) using both the model fitting and model-free spline fitting approach from the basic part of R's add-on package *grofit* [48]. While all parameter estimates are included in Files S7 and S8, summary statistics from parameter estimation are shown in Table 1.

**Table 1:** Reliability in parameter estimation.

	Value	Dataset 1	Dataset 2	Dataset 3
curves	# total	3840	3840	3264
	# without fittable models	244 (6.35%)	64 (1.66%)	44 (1.35%)
	# without fittable splines	0 (0%)	0 (0%)	0 (0%)
experimental groups	# total	768	768	864
	# without fittable models	12 (1.56%)	1 (0.13%)	2 (0.23%)
	# without fittable splines	0 (0%)	0 (0%)	0 (0%)
	# model parameter λ<0	281 (36.59%)	229 (29.82%)	205 (23.73%)
	# spline parameter λ<0	221 (28.78%)	154 (20.05%)	124 (14.35%)
	# model parameter A>400	10 (1.3%)	3 (0.39%)	12 (1.39%)
	# spline parameter A>400	0 (0%)	0 (0%)	0 (0%)
	# model parameter A<100	282 (36.72%)	245 (31.9%)	162 (18.75%)
	# spline parameter A<100	291 (37.89%)	236 (30.73%)	146 (16.9%)
spline fits	# A<100	1464	1196	583
	# A<100 and λ<0	884 (60.38%)	733 (61.29%)	485 (83.19%)
	mean λ if A<100 and λ<0	−207.6±2871.0	−6.8±8.1	−12.1±12.4
	# A>100	2376	2664	2681
	# A>100 and λ<0	106 (4.46%)	106 (3.98%)	130 (4.85%)
	mean λ if A>100 and λ<0	−3.3±4.4	−3.3±4.3	−3.0±3.5

Summary statistics from parameter estimation from the three dataset exemplars using both the model fitting and model-free spline fitting approach from the basic part of R's add-on package *grofit* (Kahm et al. 2010). Results with parameters ë<0 and A>400 indicate biologically unreasonable estimates; parameter estimates 0<A<100 approximately indicate negative reactions.

Depending on the specific dataset, between 1.4% and 6.4% of the curves could not be fitted by the modeling approach. Hence, for some experimental groups no parameter estimation was possible at all, resulting in one to twelve groups without parameter estimation depending on the dataset. In contrast, the spline resulted for all datasets, yielding parameter estimates for every group (Table 1). As mentioned above, biologically reasonable values for λ can, in principle, not be negative or exceed the last time point of measurement, whereas a reasonable A should be a positive value not greater than 400. Slightly negative values and those only slightly exceeding 400, however, can be judged as just negligibly inaccurate estimations of 0 and 400, respectively. The model-fitting approach resulted in negative estimates for λ in 23.7% to 36.6% of the groups and in A estimates exceeding 400 in 0.4% to 1.4% of the groups (yielding at least one uninterpretable parameter in between 25.4% and 39.5% of the groups).

In contrast, the spline fit yielded negative λ in only 14.4% to 28.8% of the groups; hence around 10% fewer groups with unreasonable λ point estimators. Not a single group was found with an estimate for A exceeding 400 (Table 1). Accordingly, uninterpretable values for one parameter (λ), if any, did not result in uninterpretable values for others (A). For those spline estimates with A>100 (approximately representing positive reactions), only between 4.0% and 4.9% of the lambda values were negative, and only slightly so (mean between −3.3 and 3.0 h). The vast majority of negative λ occurred for A<100 (approximately representing negative reactions). In the cases of datasets 2 (mean −6.8) and 3 (mean −12.1), these values were also only slightly negative. Only in the case of dataset 1, additionally a number of extremely low λ estimates were encountered (mean −207.6). There was little difference between model fitting and spline fitting regarding the estimated proportion of negative reactions (Table 1).

Kendall and Spearman correlations between the parameters describing the curves are listed in Table 2. In the model-fitting framework the correlation between λ and the other parameters was quite low. Also, μ was moderately correlated with A but more strongly with AUC (0.732/0.712). The correlation between A and AUC was a bit lower (0.700/0.522). Within the parameters from the spline computation, λ had even less influence on the remaining parameters. Interestingly, here μ was comparably strongly correlated with both A and AUC, and the correlation between A and AUC from the spline (0.854/0.963) was much higher than for the model. That is, λ was on average less strongly correlated with the other parameters in the case of the spline, whereas all other correlations were stronger. Accordingly, estimates for AUC correlated most strongly between model and spline, followed by μ, A and finally λ in decreasing order. In File S10, graphical representations of the overall relative behavior of the parameter estimates in all-against-all correlation plots for both the model and the spline fit approach are provided.

**Table 2:** Within-method and between-method interdependence of parameter estimates.

		Model				Spline			
		λ	μ	A	AUC	λ	μ	A	AUC
Model	λ		0.428	0.367	0.294	0.641	0.360	0.371	0.295
	μ	0.248		0.536	0.732	0.296	0.846	0.717	0.734
	A	0.362	0.320		0.7	0.324	0.538	0.748	0.700
	AUC	0.372	0.712	0.522		0.205	0.721	0.843	0.998
Spline	λ	0.571	0.131	0.225	0.211		0.299	0.285	0.203
	μ	0.940	0.837	0.272	0.620	0.025		0.723	0.736
	A	0.437	0.658	0.571	0.963	0.051	0.584		0.854
	AUC	0.372	0.713	0.523	0.999	0.046	0.621	0.963	

All-against-all correlations measured using Kendall's τ (above the diagonal) and Spearman's correlation index (below the diagonal) describe how the different curve parameters estimated using either model fitting or spline fit are associated with each other and with the corresponding parameters from the alternative fitting approach.

In Fig. 3 eight examples for the distinct types of curve shapes identified in our datasets (wells G11 and H11 for all four strains, respectively, see Fig. 1) are used to explore the specific behavior of, and the potential problems specifically associated with, model fitting and spline fitting in comparison with the native OmniLog® PM software.

On substrate H11 (Fig. 3, upper row) the respiration curves for all strains indicated positive reactions, but their shapes were substantially different. By using the curve parameters together with their CIs, the differences were easily detectable and one could intuitively comprehend the differences in curve shape. Spline fitting yielded broader CIs for λ and μ. None of the parameter estimates were biologically unreasonable.

On substrate G11 (Fig. 3, lower row), besides two common curve shapes (blue and black) two deviating data situations are shown (red and green curve), which were nevertheless common in our measurements (see File S2). The red curve reveals a primary, steep ascent followed by an interim plateau, before a second, shallower ascent conducts to the final maximum height. This behavior occurred in all ten repetitions of this experiment (see File S2). The model-based estimate for μ was lower than the spline-based one, but both were higher than the OmniLog® estimate.

The green curve describes an intrinsically negative reaction (no respiration curve), but instead a slight and linear increase in color development. This probable noise was apparently sufficient as a data basis on which model fit was possible, but some of the resulting parameters, especially the negative length of the lag phase λ, were not biologically sensible. In contrast, the corresponding spline fit yielded a positive value for λ with a broad CI. Again in contrast, the OmniLog® software yielded a value near zero for this parameter. The estimate for μ was slightly positive only from the spline fit, whereas the other two methods yielded zero values. The other parameters were rather similarly estimated by the different methods.

**Figure 4:**
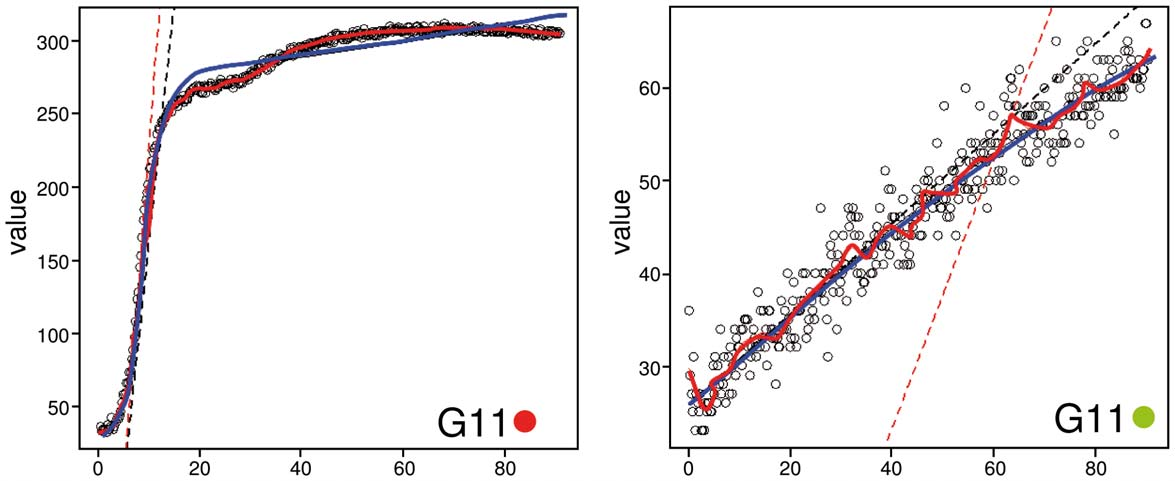
Visualization of parametric fitting and model-free spline fitting for the two special cases from G11, the strains marked red (here left) and green (here right), respectively. The raw colour intensities (black circles), measured over time (x-axis, hours) were fitted by both a parametric model (thick blue line) and a model-free spline (thick red line). The thin dashed lines indicate the maximum slope of each approach (thin dashed black line corresponds to the model fitting approach, the thin dashed red line to the spline, respectively). In the left panel the irregularity is better customized by the spline fit, whereas the model straightens it with the consequence of underestimating the maximum height (A). In case of (almost) no respiration (right panel), the fitted model apparently yielded biologically reasonable parameter estimates for µ but not for λ. In contrast, the spline approach exhibited overfitting and yielded overestimated µ and also overestimated, but biologically meaningful λ. Note the particularly broad CIs for these parameter estimates in Fig. 3.

To further explore the causes of the respective differences and characteristics of parameter estimation in certain data situations, the best-fitting model and the spline fit for the red and green curves from well G11 are shown in Fig. 4. In case of the red curve (Fig. 4, left side), the modeling method found a Gompertz exponential model to describe the data. However, the estimator for the maximum height A from the model approach is much too small, while both the OmniLog® and the spline estimates fit well to the real maximum height of the curve (see Fig. 3, G11). Apparently the model fit is influenced too much by the height of the non-typical interim plateau. In contrast, the spline was able to model the irregularity and hence to represent the curve's behavior more precisely.

The example corresponding to (almost) no respiration (Fig. 4, right side) was somewhat more complicated. Ideally, non-respiration would result in a horizontal line, and, hence, non-convergence for modeling approaches. However, the linearly increasing noise allowed a model to be fitted to the data which apparently resulted in biologically unreasonable parameter estimates *via* extrapolation; i.e., in the model the lag phase was extended to prior the beginning of the measurement (at 0 h). Other parameters such as the slope μ resulted in better estimates. In contrast, although it exhibited overfitting behavior, the spline approach was able to follow the data more precisely, apparently without the need to extrapolate. But whereas λ was estimated with a meaningful numerical result, μ was strongly overestimated. These estimation problems were also indicated by the particularly broad CIs for these parameters if inferred from the spline.

### Detecting significant differences

**Figure 5:**
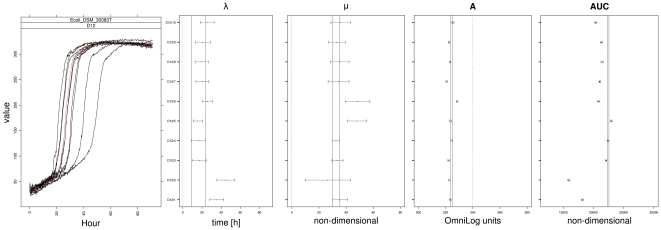
Comparison of curve-parameter point estimates and their 95% CIs for each of the four parameters lag phase (λ), slope (μ), maximum (A) and area under the curve (AUC), estimated for ten technical repetitions of respiration on well D12. Left, a plot of the raw respiration data illustrates their courses individually for each of the ten repetitions. The red curve (D12/4) was used as an exemplar for demonstrating the detection of significant differences via CIs. In the right panel, point estimates and 95% CIs for each of the four parameters from the spline approach are given for each replication. The blue lines highlight the position of the upper and lower limit of CIs from D12/4's parameters. A non-overlap of the CIs of different curves indicates a difference of a statistically detectable amount, and the distance between two intervals provides information about the expected minimum difference.

In Fig. 5 the curves from ten technical repetitions of the reaction on substrate D12 (Minocycline) are compared with their curve parameters and 95% CIs estimated using the spline approach. These curves only differed regarding the beginning of the respiration reaction.

We used the red-colored curve (D12/4) as an exemplar for demonstrating the detection of significant differences *via* CIs, which are indicated by vertical blue lines in the graphic. The two curves D12/1 and D12/2 were different to a statistically detectable degree regarding the length of the lag phase λ with a mean longer λ of 4.6 h and 12 h, respectively. D12/5 and D12/6 exhibited significantly larger slopes μ, differing in mean 15 and 14.5 units, respectively. Due to the very narrow CI for the maximal respiration A, D12/6 was identified as statistically detectable different with on average 3 OmniLog® units more respiration. D12/3, D12/7 and D12/9 had a smaller A with mean differences of 1, 2 and 1.7 units, respectively. Although all differences were statistically detectable, the user had the additional information of the effect sizes and thus was, in principle, able to use background information to decide whether the detected differences were biologically relevant. The integrals describing the areas under the curves resulted in very small CIs and thus all curves, except D12/3, were differing significantly.

**Figure 6:**
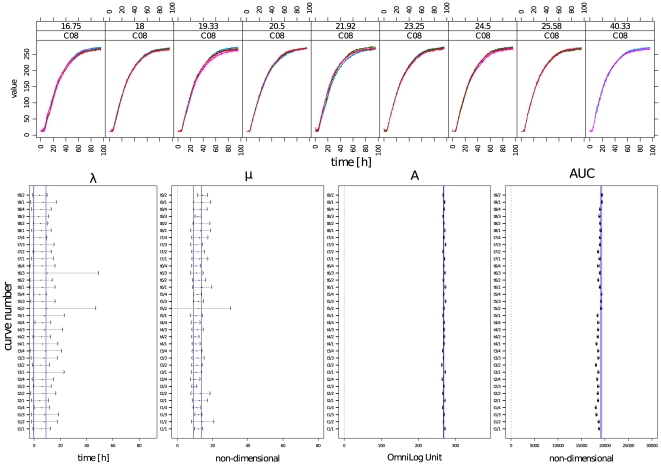
Comparison of point estimates and their 95% CIs for each of the four parameters lag phase (λ), slope (μ), maximum (A) and area under the curve (AUC), estimated for four technical replicates of respiration on well C08, in which the cells were additionally subjected to distinct pretreatments (cultivation times). The upper panels show the plot of the respiration curves of *E. coli* DSM 18039 = K12 on well C08 when grown on solid LB medium for 16.75 h (t_1_), 18 h (t_2_), 19.33 (t_3_), 20.5 (t_4_), 21.92 (t_5_), 23.25 h (t_6_), 24.5 h (t_7_), 25.58 h (t_8_) or 40.33 h (t_9_), respectively, and subsequently measured on GEN III microplates™ in the PM modus over 91 h. The lower panels shows point estimates and 95% CIs for each of the four parameters from the spline approach. The blue lines highlight the position of the upper and lower limits of the CIs from repetition no. 4 at t_5_. A non-overlap of the CIs of different curves indicates a difference of a statistically detectable amount, and the distance between two intervals provides information about the expected minimum difference.

The results from the time series approach in the third dataset are shown in Fig. 6 for substrate C08 (L-Rhamnose). Curve 20, the fourth repetition from time point 21.92 h (t_5_) was chosen as an example and the corresponding CI limits highlighted. For both λ and μ, all other CIs overlapped with that from curve 20, indicating no detectable differences between the curves. Considering the maximal respiration A and the integral AUC, several CIs did not overlap with that from curve 20, but the effect size for the maximal respiration is at most 4 OmniLog® units ( = 1.5%) for A and 978 units ( = 5%) for AUC. Again, the user was now free to decide whether these differences should be regarded as biologically relevant.

**Figure 7:**
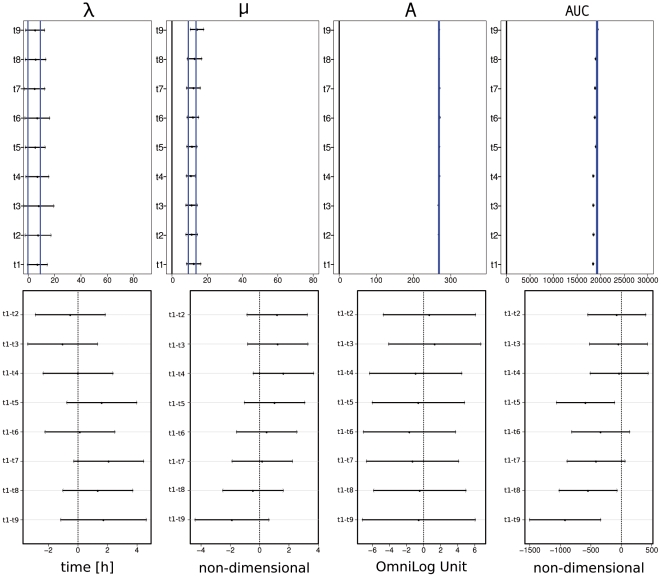
Visualization of group-wise representations of the four curve parameters lag phase (λ), slope (μ), maximum (A) and area under the curve (AUC). The upper panels show the results from the preliminary calculation, a simple calculation of group means of confidence limits and point estimators. The groups, here the distinct pretreatments (cultivation times t1 to t9), are given on the y-axis. For orientation, the blue lines highlight the position of upper and lower limit of CIs from repetition no. 4 at t_5_, in analogy to Fig. 6. In the lower panels the 95% CIs for the differences of group means are represented. The set of user-defined comparisons was calculated for the point estimators of each of the four parameters lag phase (λ), slope (μ), maximum (A) and area under the curve (AUC). Since these are CIs for the differences between the means, a non-overlap with zero indicates a statistically detectable difference between the considered group means of the examined curve parameters.

Regarding the comparison of group means, Fig. 7 shows both the preliminary visualization using the mean CIs calculated over the groups (upper part) and the CIs for the differences between the means resulting from the simultaneous comparison procedure (lower part). The mean CIs can be used analogously to the strategy described above: overlapping CIs indicate no detectable difference between the groups, while non-overlapping ones indicate such differences.

The multiple-mean comparison testing procedure also provides 95% CIs, but for the differences between the group means (here: the considered parameter estimators), thus yielding precise information about the significance of the differences between the groups regarding the considered parameter(s).

To examine whether it is valid to subtract the negative controls (A01) from the measurements from all other wells before estimating curve parameters, we compared the parameter values for maximum height (A) from the A01 with that from selected wells with a negative reaction. Our findings suggest that the negative control might display a reproducible, strain-specific growth-like behavior, and even though these curves are shallower than unambiguously positive reactions, their maximum height can well be larger than that of typical negative reactions on the same plate. This makes it impossible to regard it as an approximation of an error term to be subtracted from the measurements from each other well. These findings are described in detail in File S9.

### Which, and how many, classes of curve shapes?

**Figure 8:**
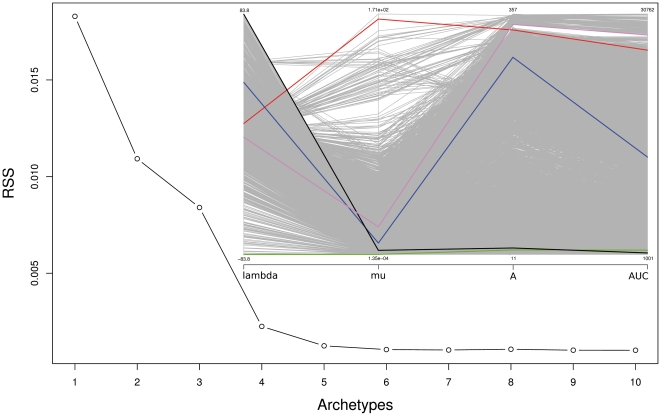
Results from an archetype analysis of the four parameters estimated from the PM curves obtained from the 1^st^ and the 2^nd^ dataset using the smoothing splines. The outer figure is a scree plot in which the residual sums of squares (RSS, y-axis) are plotted against the corresponding predefined numbers of archetypes (x-axis). Apparently either four or five archetypes are optimal according to the “elbow criterion”. The insert (upper right) is a parallel coordinates plot showing the original measurements (gray lines) as well as the optimal archetypes (green, black, blue, violet and red lines) obtained if five archetypes are requested. On the x-axis, the names of the curve parameters are indicated. The minima and maxima of the four y-axes are also indicated. For an interpretation of the archetypes, see the main text.

Analysis of archetypes (Fig. 8) indicated that either four or five archetypes are optimal. For five as predefined number, the resulting curve archetypes (insert in Fig. 8) could be interpreted as follows: non-reaction with negative λ (an artifact, see above) (green line); non-reaction without such an artifact (black line); curves with a delayed start, i.e. reactions with a long lag phase λ, a relatively low μ and, thus, a rather low AUC/A ratio (blue line); early starting curves with a low λ, a moderate μ but nevertheless both high A and AUC (violet line); and, finally, rapidly accelerating curves with a moderate λ but a high μ, which reach an almost as high A and AUC (red line). These rapid accelerators had approximately the same A/AUC ratio as the early starters, but occurred much more seldom in the datasets (Fig. 8).

## Discussion

### Visualization of PM raw data

When facing huge and complicatedly structured datasets such as the PM ones discussed here or that commonly occurring in other -omics analyses, the only way to get a comprehensive insight into the experimental results is a suitable graphical raw data representation. Such exploratory graphics have to be comprehensible in short time but also be highly informative [71]. The convenience of an exploratory graphical representation depends mainly on its flexibility. Hence, the graphics should be easily adjustable to individual users' requirements to enable them to discover potentially all interesting and important features of the data. In contrast to the severely limited options for comparing different strains on the same substrate in a single pre-specified plot, as provided by the OmniLog® PM software [34], the user needs to compose the data unaffiliated.

In this study we explored an open-source solution for these specifications and showed that curve kinetics offer a more powerful data visualization than level plots [37]. By using the function *xyplot()* from the *lattice* package [60] highly structured graphics can easily be produced while retaining flexibility by systematically decoupling the various elements of a display. Itemization by substrate, tested strain or even repetition number was quite simple and constraints regarding the number of displayed curves or the position of the subpanel were not imposed at all. We thus recommend this or equivalent visualization approaches for PM data. A potential improvement compared to Fig. 2 is the inclusion of the names of the substrates in addition to or instead of the mere coordinates of the wells.

### Parameter estimation from respiration curves

The information content of the longitudinal PM raw data is a multiple of what an endpoint measurement could ever provide. A suitable analysis strategy thus has to be able to summarize this information and eliminate noise. These requirements can be met by model-fitting and spline-fitting approaches aiming on both dimension reduction and noise reduction [72]–[73]. With *grofit*, the result is a set of four parameters sufficient for comprehensively describing the curves' shape. The main goals of a subsequent data evaluation would be the determination of the influence of different substrates, organisms investigated, or pretreatments, *via* the comparative characterization of respiration over time.

Although the OmniLog® PM software [34] is, in principle, able to provide a compilation of parameters, their computation is based only on few data points, potentially leading to the neglect of relevant data features. Here, we applied two alternative methods for extracting the four curve parameters λ, μ, A and AUC. The aim was to find a reliable estimation method that was able to deal adequately with curves' potential deviations from the common sigmoid shape [42].

Our results indicate, however, that the parameter estimation procedures perform best if applied to curves that follow the typical sigmoidal shape. But the parameters A (maximum height) and AUC (area under the curve) are less influenced by possibly uncommon shapes than the lag phase (λ) and the slope (μ). When comparing the two main approaches for curve description, it turned out that the spline smoother is flexible enough to follow even extreme curve shapes and is therefore superior for general parameter estimation, while the model-fitting approach appeared to be more constrained by the underlying model equations and straightened the curves to much. While 14% to 28% of the estimates for λ were biologically unreasonable in a strict sense (negative), most of these were only slightly negative and could safely be regarded as minor mis-estimates for 0.0. Also, the interpretability of the other parameters was not affected in these cases, and extremely negative λ can still be qualitatively interpreted as indicative of overall negative reactions.

The high amount of negative estimates for λ suggests that there is still space for algorithmic improvement. In this study, the default parameters for the smoothing spline and the number of knots were used, since the evaluation of best-performing parameters was beyond the scope of this study. However, the selection of these two kinds of parameters is the critical step in this method [42]. Also, other spline families and generalized additive model frameworks would exhibit interesting features for curve fitting by imposing monotonicity constraints on smooth effects and on ordinal, categorical variables [74]. We cannot exclude that as yet unimplemented models would outperform the ones considered here or even the spline fit, but in the current situation we regard the use of splines as the best recommendation that can be provided to users interested in fitting PM curves with R.

Compared to both model and spline methods, the slope estimates from the OmniLog® PM software [34] tend to be lower if the underlying curve is not perfectly sigmoid-shaped or the respiration reaction is finished long before the measurement is stopped (G11 in Fig. 3, red and black curve, respectively). Since the OmniLog® PM software assumes that any reaction is symmetric around the inflection point, the slope is underestimated in the case of a secondary increase, which extends the distance between the time of inflection and the one of the maximum height. In contrast, the AUC estimates by the OmniLog® PM software [34] are slightly larger than those by the spline and model approaches, particularly for steep curves (Fig. 3). As the native PM software represents the curves as series of rectangles, this deviation is most likely an overestimation and is expected to increase if more steep curves are encountered. Based on these results we favour the spline-based approach to parameter estimation over the native PM software not only because it provides CIs but also because its point estimates are less prone to bias due to the described irregularities in curve shapes.

However, the spline-based approach exhibits overfitting behavior in the case of certain curves that strongly deviate from a sigmoidal curve shape. This appears to occur especially when almost no reaction takes place, as shown in Fig. 4 (left panel). Although the default smoothing parameters obviously allow for very flexibly bent curves resulting in that overfitting behavior, only the parameters λ and μ are affected and result in broad CIs, while A and AUC are hardly affected. One way out could be the selection of more suitable smoothing parameters. Alternatively, the methods for the extraction of the parameters from the spline could be revised. Especially the estimation of μ and λ, which is currently based on a a single value from the fitted spline, offers potential for improvement.

It is well known that phenomena such as autocorrelation (which is usual for growth curves) and non-homoscedasticity of the residuals violate the underlying assumptions of model- and spline-fitting [42], [43]. When dealing with high-throughput datasets such as the PM ones, however, the detailed assessment of a potential violation of the assumptions made when fitting each curve is not practicable. Moreover, while for instance the spline might overfit the data in such situations, it is here only used for smoothing each curve before extracting the four abstract parameters of interest. It is thus unlikely that potential violations of the underlying assumptions of the fit adversely affect the unbiasedness of the parameter estimates. This might explain why the spline appears more robust than the other methods if applied to PM data. While the assumptions of *ad hoc* approaches such as those implemented in [34] are, in general, less explicit, it is nevertheless apparent that they are frequently violated, too (Figs. 3, 4).

### Detecting significant differences

To enable the user to extract all necessary information, we provided a feasible graphical solution displaying the point estimator together with its CI limits. The function *xyplot()* from the package *lattice* [60] already provides the here presented outputs; only little adaption of the input data is necessary (but see below). The straightforward assembly of different curves' characteristics in a single overview facilitates the interpretation and comparison of user-defined data subsets arranged according to technical and/or biological repetitions or other aspects of the experimental design.

With two exemplars (Figure 5 and 6) we familiarized the reader with the application of CIs to PM data for detecting (in-)significant differences. The demonstrated tool yielded valuable information about the range of variability of each point estimator on the corresponding scale. Thus, the user was enabled to recognize statistically detectable differences which he could further interpret regarding the specific biological relevance in each individual question. With the example in Fig. 6 we demonstrated a further important approach. If, conversely, the experimenter wants to corroborate that a difference between the curves is not detectable (by defining a threshold *a priori* as the maximum allowed difference between the respective parameter estimates), the CIs provide a comprehensible solution (by allowing one to assess whether the expected mean difference is significantly larger than the threshold). This allows one to assess whether reproducibility is given or whether the experimental procedure needs improvement, which is important for industrial applications or for research questions aiming at the identification of strains according to their metabolic features. For instance, Fig. 6 shows that the dependency on the time of growth is negligible for this specific combination of organism and well and, hence, the protocol needs not be further standardized regarding the duration of growth.

It may often be of interest not to compare single curves but distinct groups of curves. In Fig. 7 an example for the comparison of experimental group means, which is the method of choice in data evaluation for most biological questions, was shown. Starting with the preliminary method of calculating mean CIs and their graphical representations, the user is encouraged to uncover interesting data features based on impartial calculations. But this approach can only yield preliminary information as it is not a valid testing procedure.

Using the more sophisticated simultaneous calculation of differences of user-defined means in combination with the visualization of their CIs, the experimenter is empowered to investigate the data set more specifically regarding the biological hypotheses. The advantages of simultaneous CIs for drawing testing decisions are that the significance, relevance, and direction (increase or decrease) of the effect of interest, as well as the uncertainty concerning the estimates, can be interpreted in a scale close to that of the measured variable, which is often easier than interpreting p values in the scale of probability [75].

Since the width of a CI is a critical measure in the interpretation of testing decisions, a common spline fit for a bundle of repetitions in combination with the above mentioned improvements of spline fitting itself would be of interest for further method developments. On the other hand, the estimation methods for the curve descriptive parameters should be regarded as an interesting point for improvement. As mentioned above, μ and λ are sensitive to uncommon curve shapes, at least partly because of their estimation procedure.

Beyond the here proposed strategies for testing local hypotheses, global-hypothesis frameworks, as they are known, e.g., from the already well explored gene-expression microarray analyses, should be considered. For example, comparisons between complete plates measured from distinct strains or treatments could be managed by a difference-of-means approach. To get the results from the distinct wells comparable to each other, they would need to be normalized by, e.g., dividing by the well-specific means calculated over all plates. The thus normalized parameter estimates could regarded as one sample per plate or groups of plates and accordingly compared against each other.

### Which, and how many shape classes of curve shapes?

The conducted archetype analysis [63] indicated that assuming only two classes of curve shapes is suboptimal, even if one corrects for the fact that at minimum two classes are necessary to represent the non-reactions alone due to the negative estimates for λ. Two to four archetypes were necessary for optimally representing the positive reactions, apparently because of fundamental differences in curve shape with a rather straightforward interpretation (Fig. 8). Since the number of necessary archetypes depends on the analyzed dataset, it is currently hard to recommend a predefined number of classes or even a rule of thumb. Larger datasets with even more distinct curve shapes might require more archetypes according to the RSS criterion, whereas biological background information might indicate even distinct numbers of categories. Anyway, the application of archetypes presented here already shows that a biologically meaningful post-processing of PM measurements *via* the parameter estimates is possible. Of course, other classification algorithms could also be applied such as k-means partitioning or even hierarchical clustering [76]. Even if only the discrimination between positive and negative reactions was of interest, automatically classifying the observed curve shapes by assigning them to predefined clusters of curves or “typical” curves would be necessary for high-throughput processing of the Phenotype MicroArray data.

### Conclusions and outlook

With the here presented approach to OmniLog® PM data analysis highly structured graphics can easily be produced while retaining the flexibility of systematically decoupling the various elements of a display. Itemization by substrate, tested strain or even repetition number is quite simple and no constraints about the number of displayed curves or the position of the subpanel are imposed at all.

The smoothing-spline method for dimension and noise reduction appears to prevalently result in more meaningful parameter estimates than parametric model fitting when applied to PM data. *Via* curve-fitting the user can extract more information from the same experimental data than with any of the previously established techniques, particularly those reducing the data to binary states (positive vs. negative reactions). The inferred parameters can be used to classify the curves, and with our dataset more than just the categories positive/negative were optimal, even though the resulting archetypes could be easily interpreted. Dimension reduction of the curves followed by automated classification and identification seems to be of high future potential, particularly if combined with CIs, for the computational high-throughput processing of the raw data. This kind of data treatment will most probably also enhance the usefulness of high-throughput phenotyping for data modeling in microbial pathway genomics [77].

In conjunction with the proposed parameter estimation using models or preferably splines, the experimenter is free to define limits within which statistically detectable differences are not considered biologically relevant, which makes this method easily adaptable and more powerful than conventional mean comparison procedures [35], [78]. The proposed method intentionally resigns any multiplicity adjustment, because the analyses are intended to find preferably all interesting phenomena.

Although the strategy introduced here depends on CIs calculated on the basis of single curves, the approach could be easily extended to include the calculation of mean curves and corresponding intervals [47], or to summarize the parameters from associated curves and perform CI computations and comparisons of multiple means with the resulting values [78]. Considering the very low sample size in hitherto published PM experiments, the chance to apply the hereby acquired information in sample size calculation should be emphasized. Usually one aim of statistical analyzes is to find a detectable difference regarding an *a priori* chosen alpha [78]. Since statistical testing provides primarily this detection of statistical significance, researchers frequently interpret only this information, irrespective of the size of detected mean differences, i.e. the effect size [36], [62]. However, for the majority of experimental investigations, especially in physiology [79]–[81], often a minimum effect size is known for an effect to be biologically relevant. Our approach can extract information from preliminary experiments that can be used to compute the specific sample size required for the detection of biologically relevant differences with a sufficiently high confidence level in subsequent experiments. Thus, experimenters are enabled to improve their experimental design for satisfying their specific constraints and requirements more thoroughly.

As demonstrated here, for a comprehensive comparison of the curves several parameters have to be considered to come to a meaningful decision. This is connected to fundamental ideas from multivariate data analysis [82], where several features of one object are recorded and analyzed together. One alternative to avoid the application of such more sophisticated methods could be the combination of several parameters into one, as proposed by Wang et al. [83], who multiplied slope and area under the curve. As shown here, the curve parameters (among them AUC and μ) can be strongly correlated. We explored the results for the product of AUC and μ using simulated datasets constructed by (i) using the empirical values for both parameters estimated in the course of the study and (ii) generating all possible combinations (irrespective of whether they occurred in the real data). In these data, wee found very similar numerical values for the cases “high AUC × low μ” and “low AUC × high μ” although they would originate from totally different curve shapes (data not shown). We would thus caution against using simple ratios or products for the combination of parameters, even though we cannot exclude that more complex algorithms were more successful. The AUC is expected to be affected by all other parameters and could well be used for summarizing the curves, but some information loss is expected to always occur if the four parameters are to be represented by a single one.

Even though the analysis of the biological causes for the respiration behaviors of the here tested strains is beyond the scope of the study, a few remarks on the study design and implementation of controls should be placed. On the GEN III plates the A01 well is defined as the control well, containing no substrate. By construction no reaction should occur on this well unless some kind of artifact was involved. The vendor's recommendation is, understandably, to adjust the experimental procedure until this point is met [34]. However, further the user is instructed to subtract the, hopefully low, A01 curve from all other curves before proceeding with data analysis. Considering the fact that growth curves are seldom strictly additive or multiplicative in a biologically meaningful sense [84], this approach raises several concerns regarding the impact on the shapes of the resulting curves and the character of thereby introduced biases. From our point of view, the experimental conditions should be first tried to be customized until there is no detectable positive reaction in A01 anymore [23]. We strongly encourage users to use the raw data for further analysis without subtraction of A01 from all other curves. We believe that the curve from A01 and its parameters are of higher benefit when used as thresholds for the dichotomization of experimental outcomes. The only exception would be a scenario in which the values in the negative control could be regarded as some kind of background noise which actually behaves additively with respect to the signal from the curves, if any. Our observations disagree with this scenario, however, as many intrinsically negative reactions in other wells were shallower (i.e., showed lower values of A) than those in A01 (see Files S4 to S6). Surprisingly, the shapes of the curves were strain-specific, and for *Escherichia coli* DSM 30083^T^, if pooled over all replicates, the values of A in well A01 turned out to be significantly larger than those in, e.g., well D03 (see File S9).

In our assessments of the PM technique we observed a series of other experimental sources of errors. One of them is a false-positive color development due to some chemical conversion of the redox dye, actually not caused by respiration (data not shown). Especially pentose sugars such as L-arabinose or xylose might be susceptible to these reactions (B. Bochner, pers. comm.). Thus, we recommend to measure one plate inoculated with only the inoculation fluid but no cell material and to check if such false-positive reactions occur. Wells with such reactions should be excluded from further analysis, since their color development cannot safely be attributed to a physiological reaction.

To conclude, we believe to have demonstrated that tools provided in the free statistical software environment R can be successfully applied to PM data. These tools allow the user to visualize the kinetics in several meaningful respects, to conduct parameter estimation and, hence, dimension and noise reduction with the measurements and to detect statistically significant differences between the curves. All of these techniques can be conducted after selecting and rearranging the data in a sensible way depending on the respective scientific questions of interest. The outcome can even be used to improve the experimental setup itself as, e.g., by determining the necessary minimum number of replications. Additional work is necessary, however, to optimize details of the parameter estimation procedure (see above), and particularly to integrate all mentioned tools together with data input and output, and addition of metadata, in an easy-to-use R package. We recently released the first version of such a package, ‘opm’, by making it available at the comprehensive R archive network CRAN (http://cran.r-project.org/web/packages/opm/index.html).

The application of more sophisticated spline estimation methods might solve the remaining difficulties. One main issue is the selection of a suitable degree of smoothness. Approaches such as cross-validation [42], generalized cross-validation [85] and the application of information criteria like AIC or BIC [42] into the fitting procedure could be assessed. In this context a common spline fit over several (technical and/or biological) repetitions provides an interesting starting point for an improvement of the testing framework, as it would automatically take the various sources of variation into account.

Alternatively or additionally the methods for parameter extraction from the spline could be critically revised. A more sophisticated estimation method for the length of the maximum slope μ and the lag-phase λ, possibly making use of information from the second spline derivative, would probably be able to deal with the so far frequently suboptimal spline fits for these parameters in the case of intrinsically negative reactions.

With the here established strategies for data processing and analysis, the results from Phenotype Microarray experiments are commuted in a framework similar to that for the thoroughly acquainted gene-expression microarray analysis. Thus, the next steps leading to functional data analysis would be to test the applicability of statistical analysis tools such as global multiple testing procedures [86], pathway analyses or model-building procedures [87].

## Supporting Information

File S1 Raw measurements from dataset 1 in CSV format.
(CSV)

File S2 Raw measurements from dataset 2 in CSV format.
(CSV)

File S3 Raw measurements from dataset 3 in CSV format.
(CSV)

File S4 Plots of all respiration curves from dataset 1 in PDF format.
(PDF)

File S5 Plots of all respiration curves from dataset 2 in PDF format.
(PDF)

File S6 Plots of all respiration curves from dataset 3 in PDF format.
(PDF)

File S7 Parameter estimates from all respiration curves and their CI limits in CSV format.
(CSV)

File S8 Parameter estimates from all respiration curves and their CI limits as R code.
(TXT)

File S9 Behavior of the negative controls compared to negative reactions in other wells.
(PDF)

File S10 All-against-all correlation plots of the parameter estimates.
(PDF)
