# Supplementary figures and images for: Visualization and Curve-Parameter Estimation Strategies for Efficient Exploration of Phenotype Microarray Kinetics
Source: PLoS One. 2012 Apr 20;7(4):e34846. doi: 10.1371/journal.pone.0034846 (PMC3334903; doi:10.1371/journal.pone.0034846)

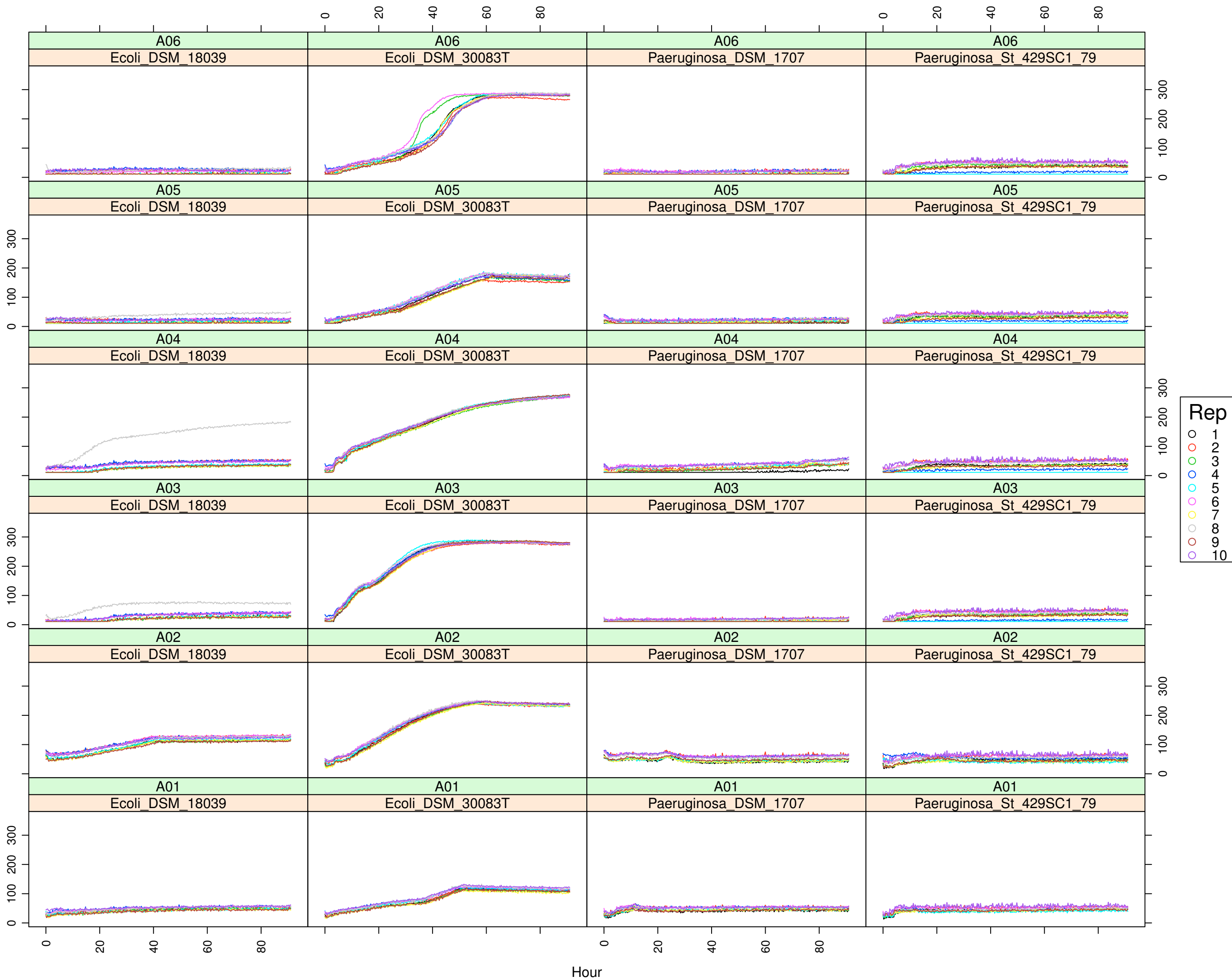

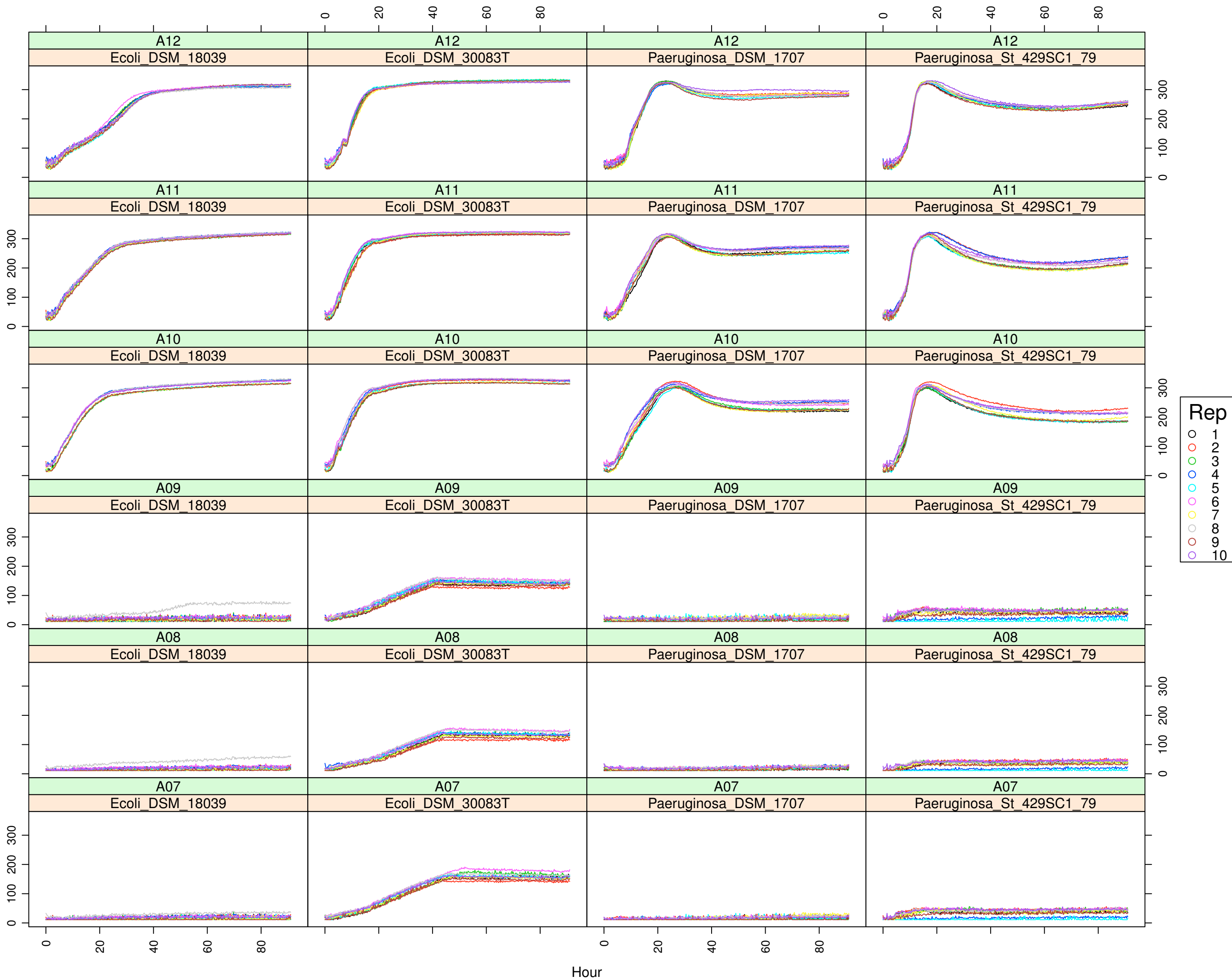

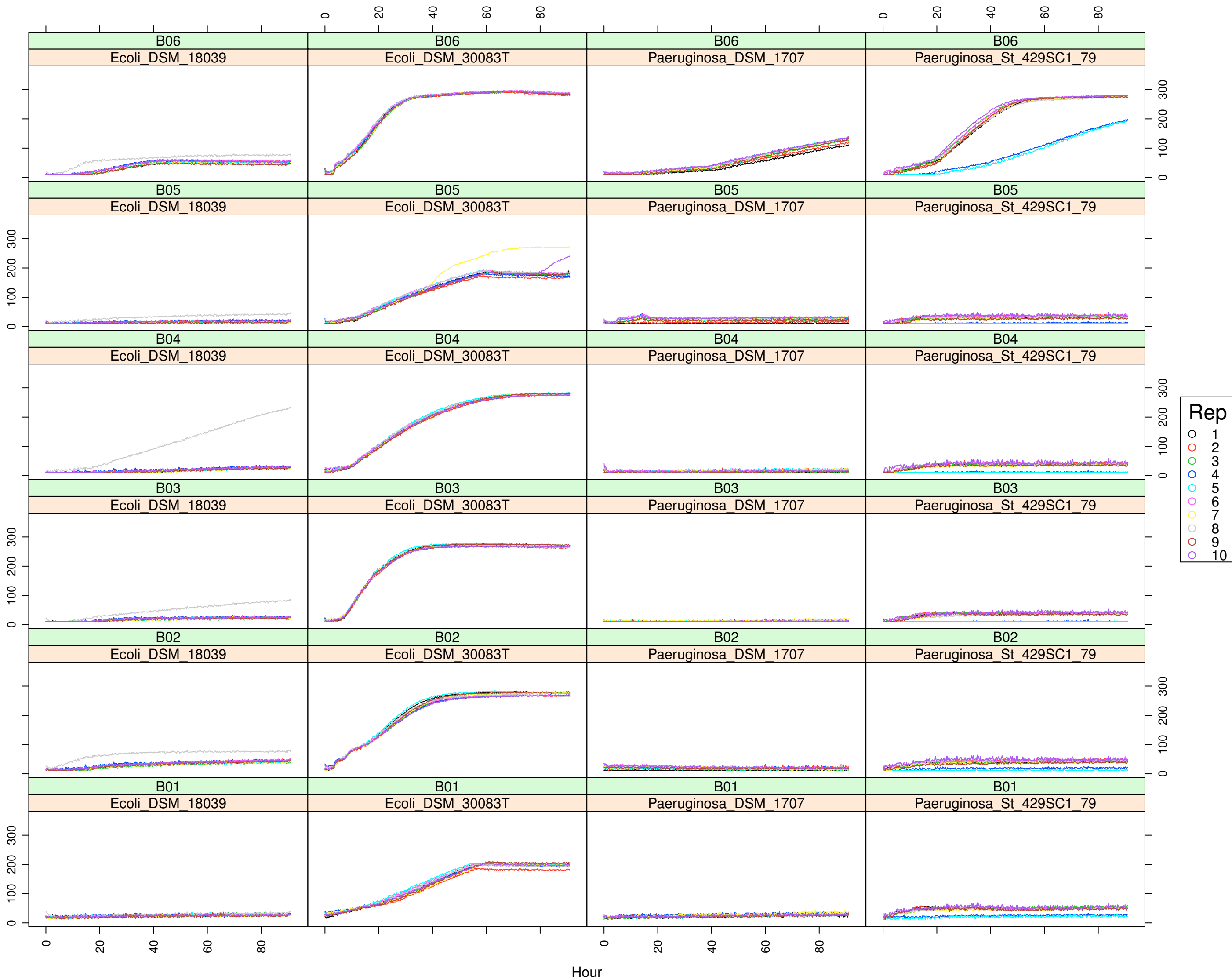

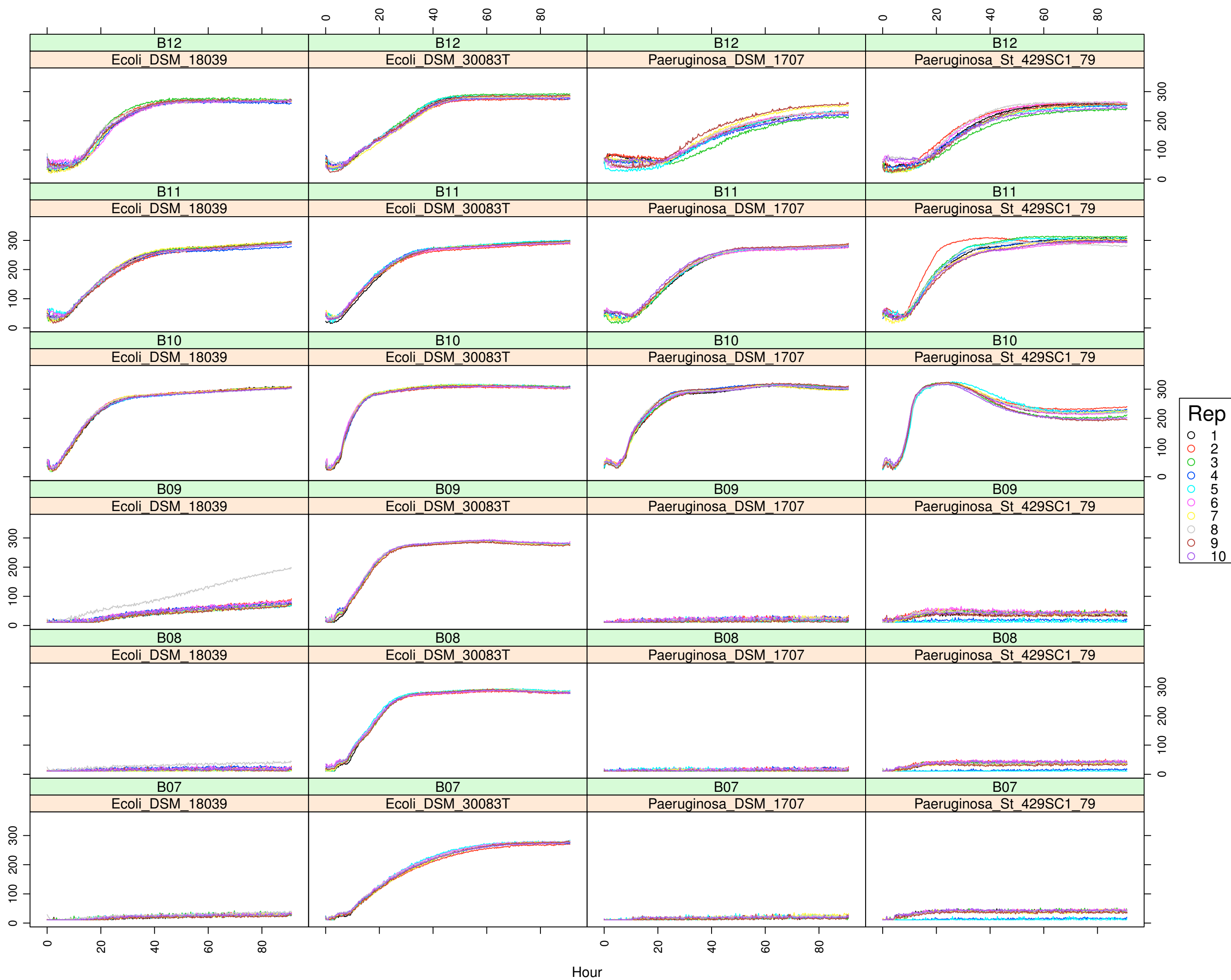

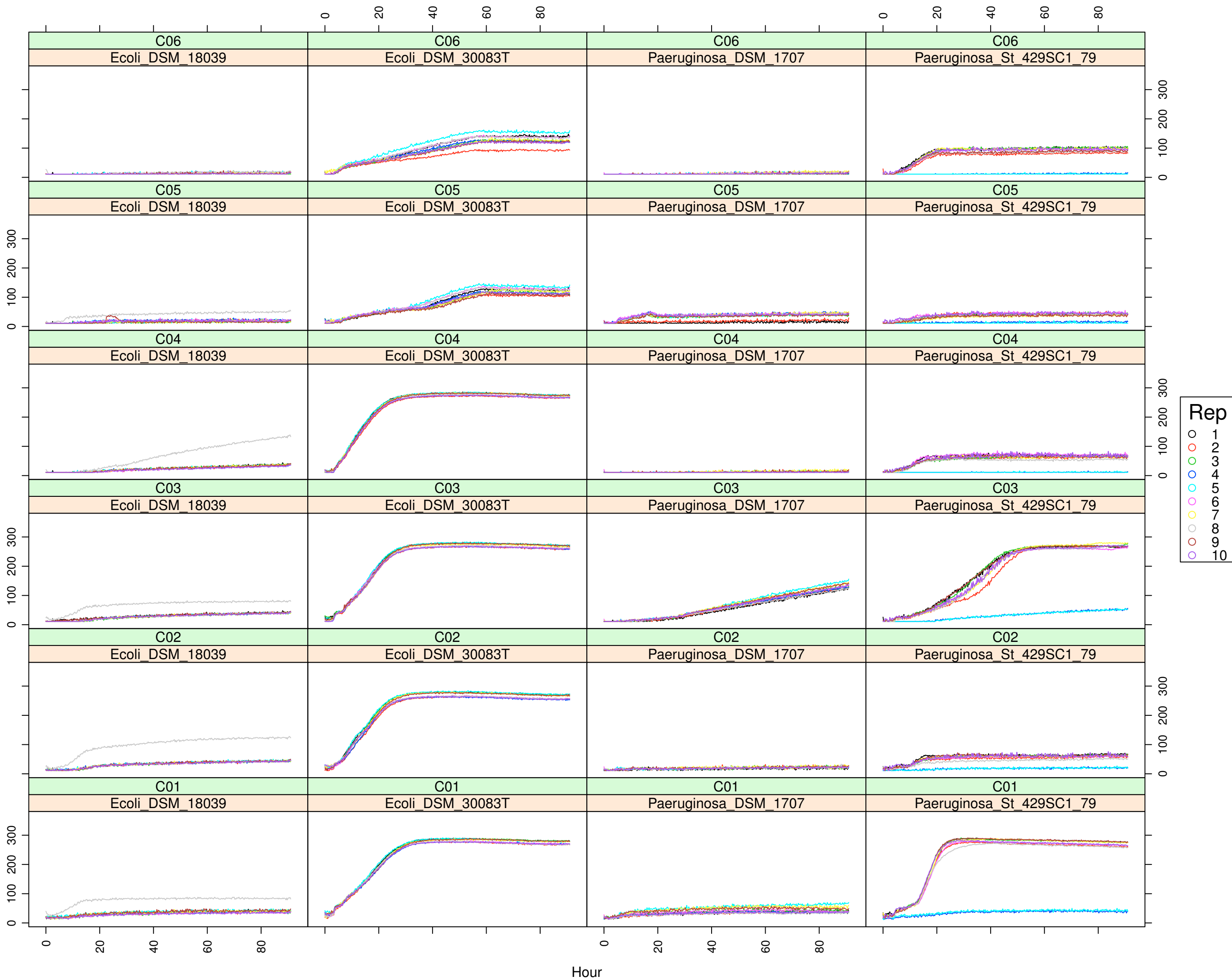

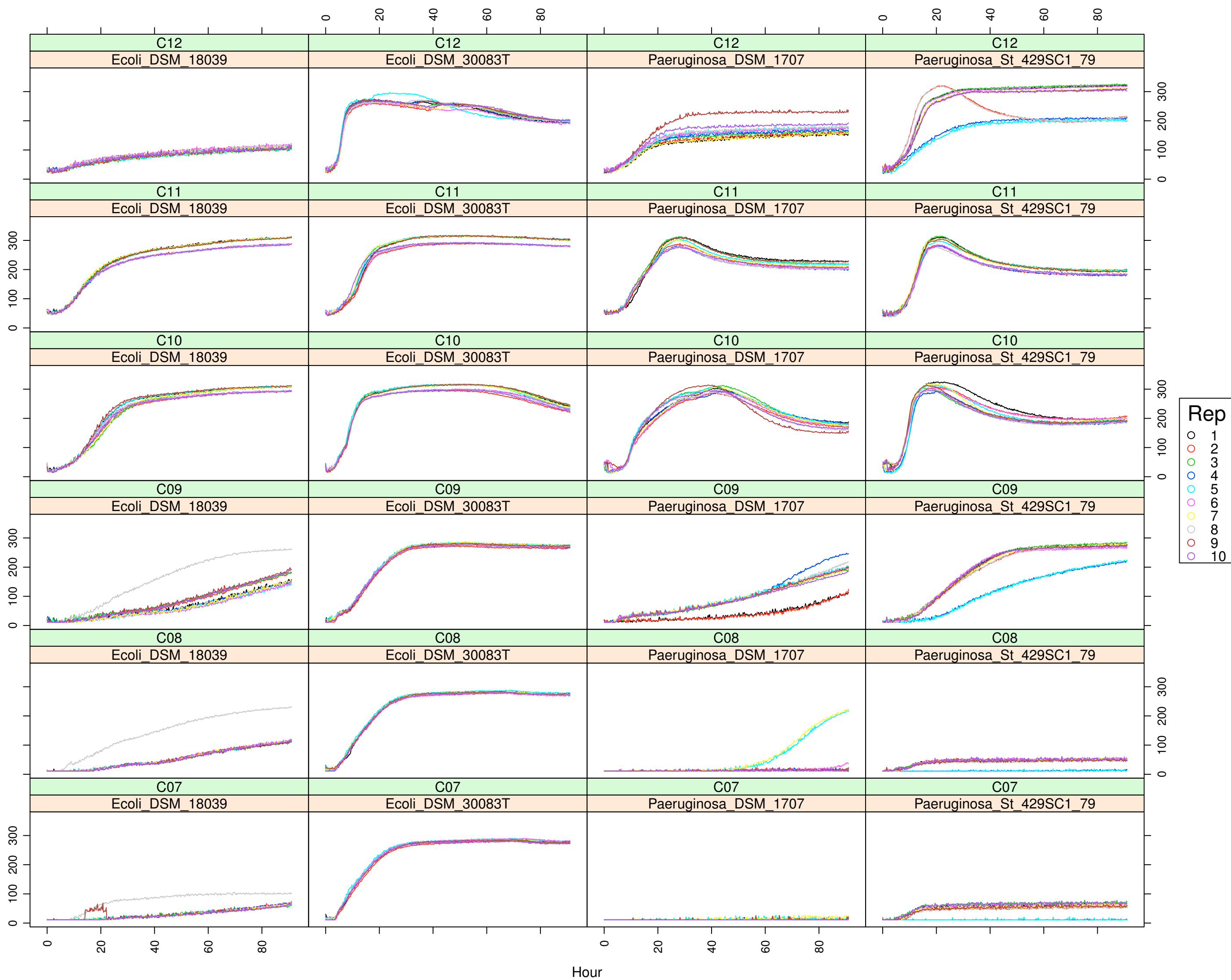

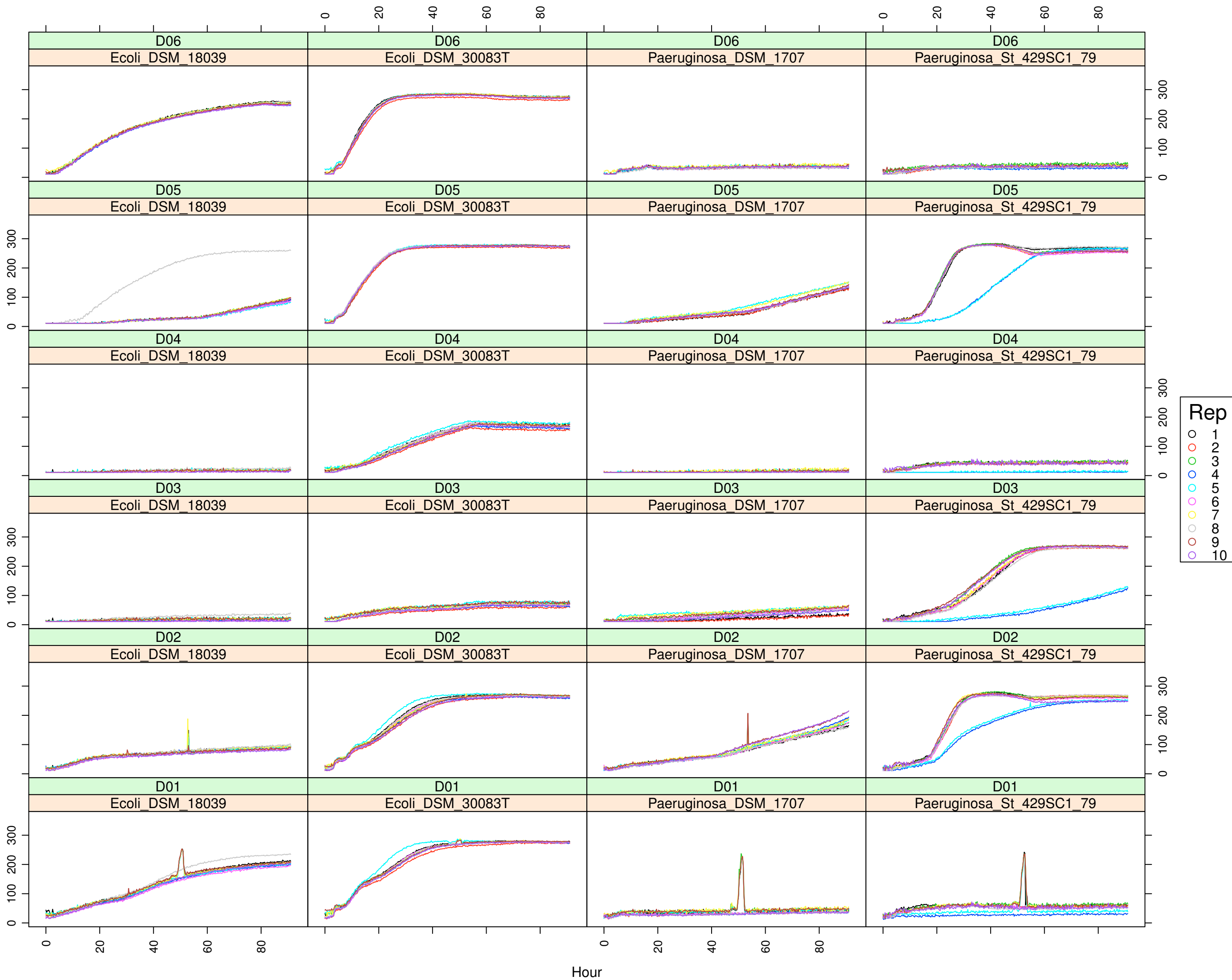

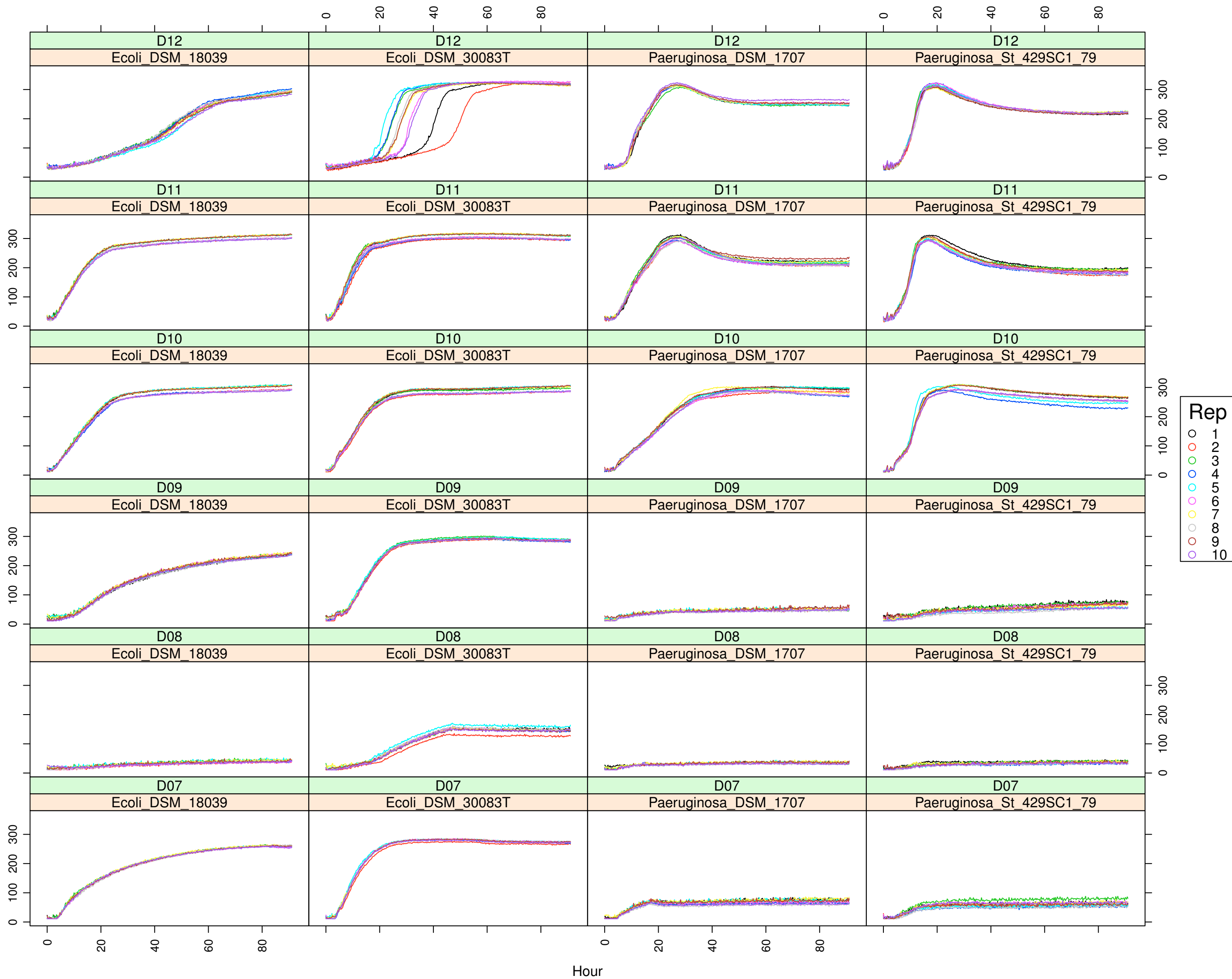

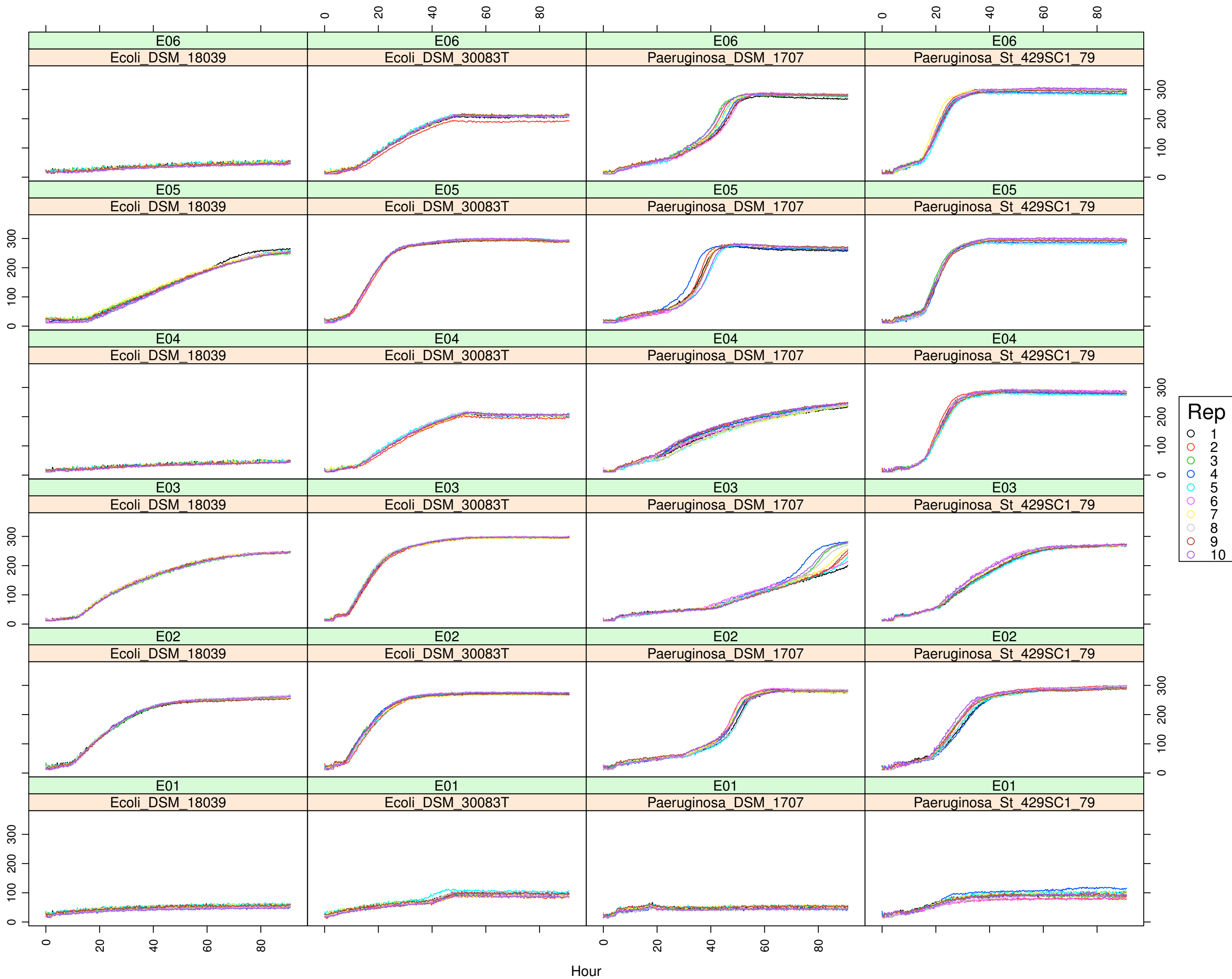

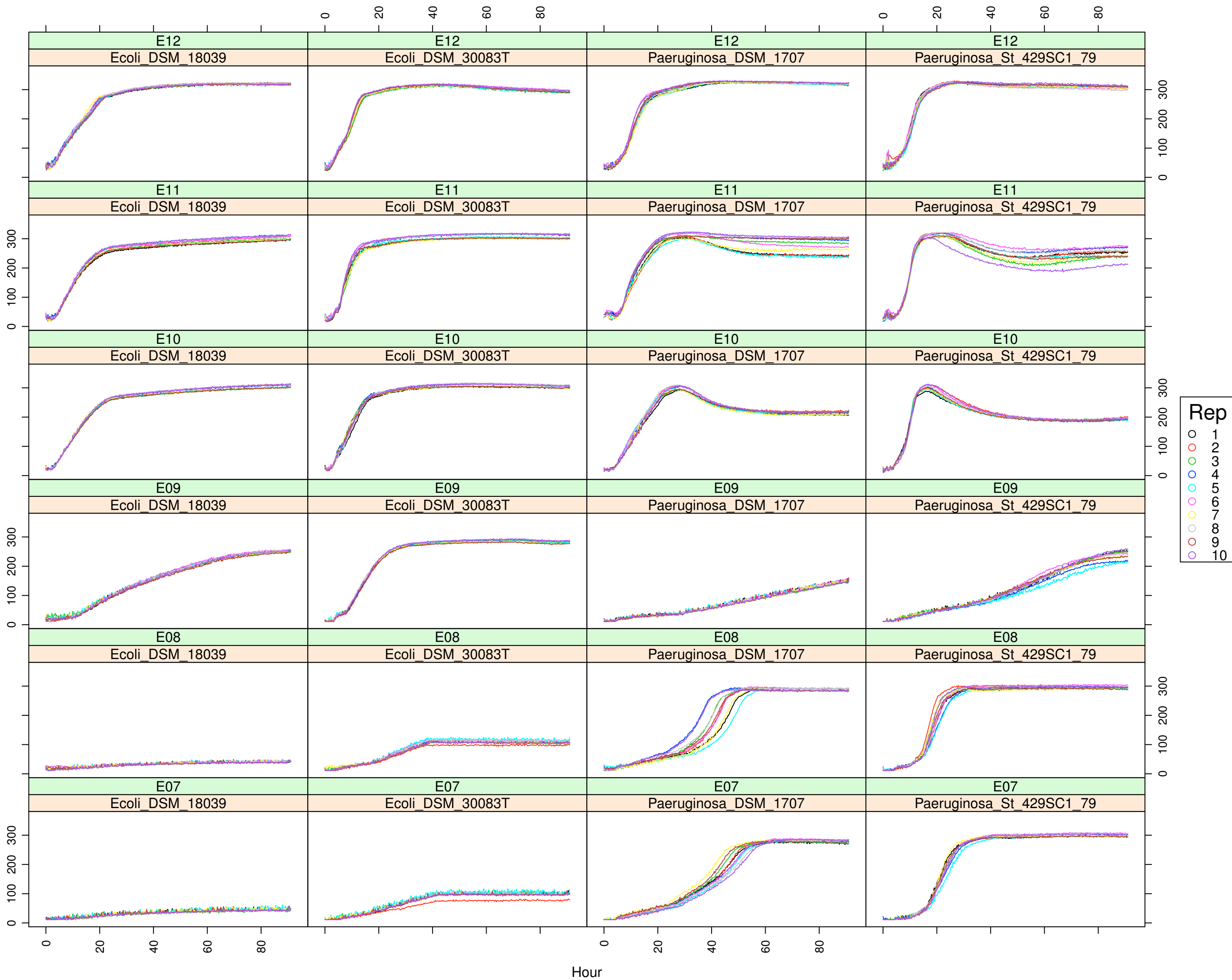

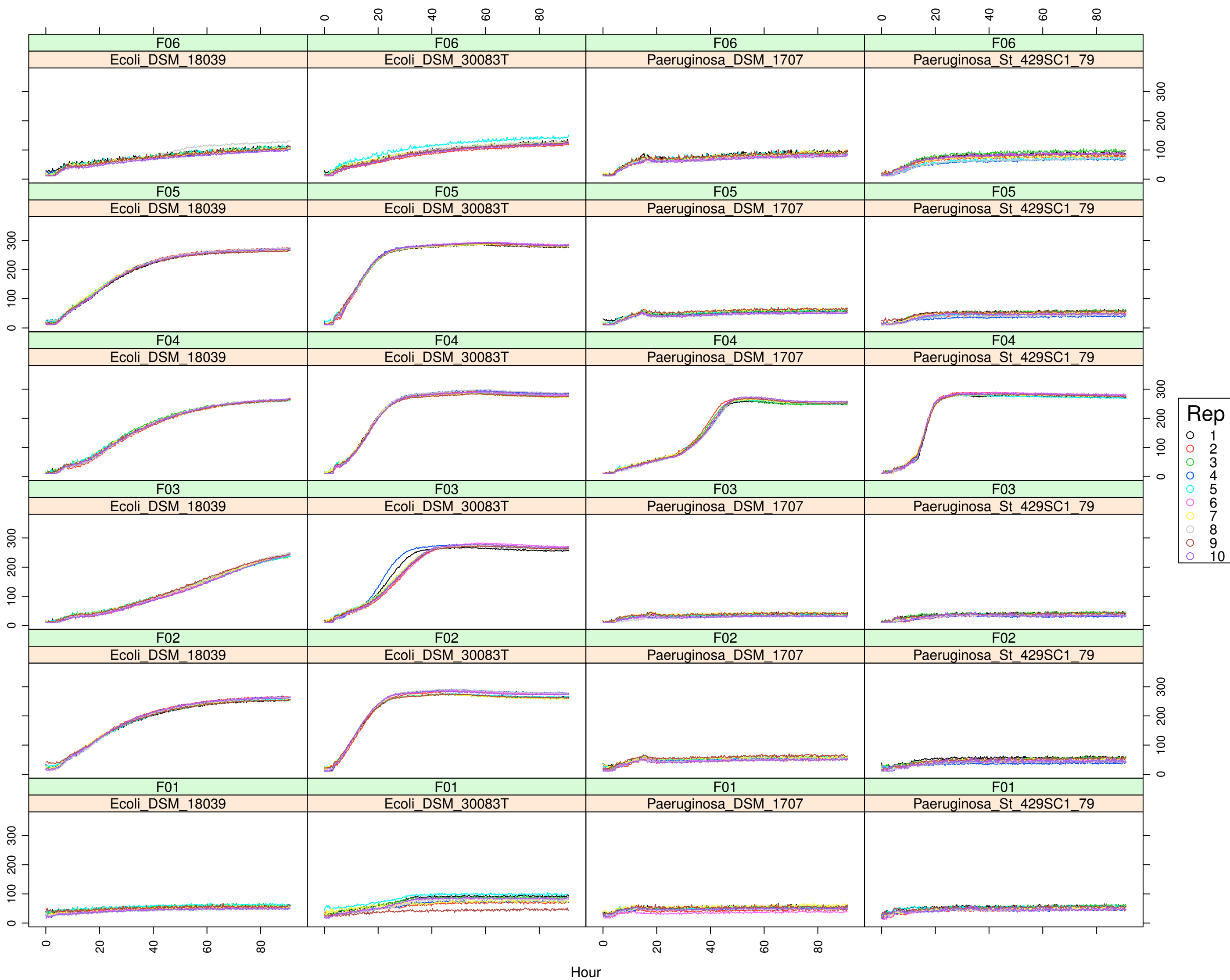

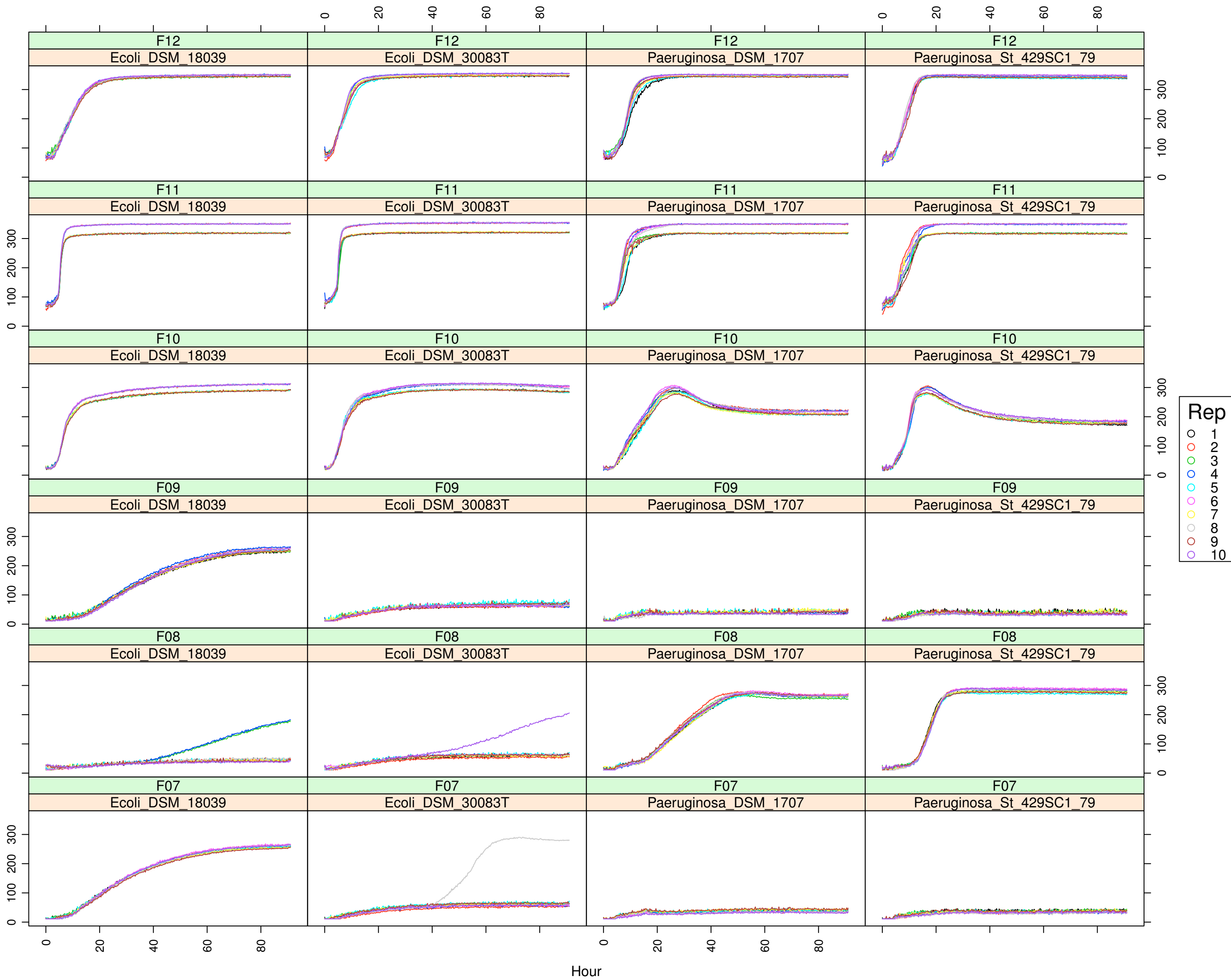

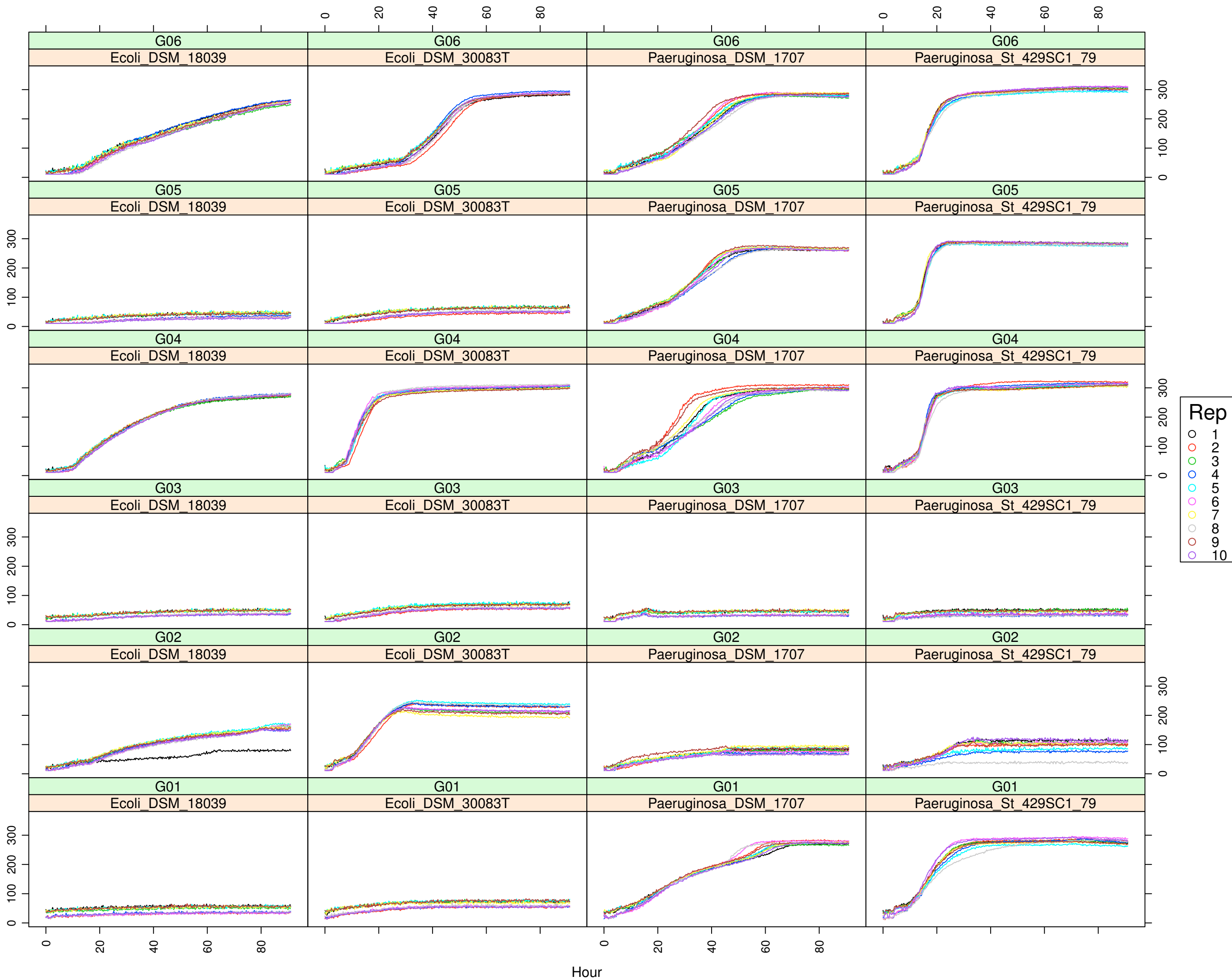

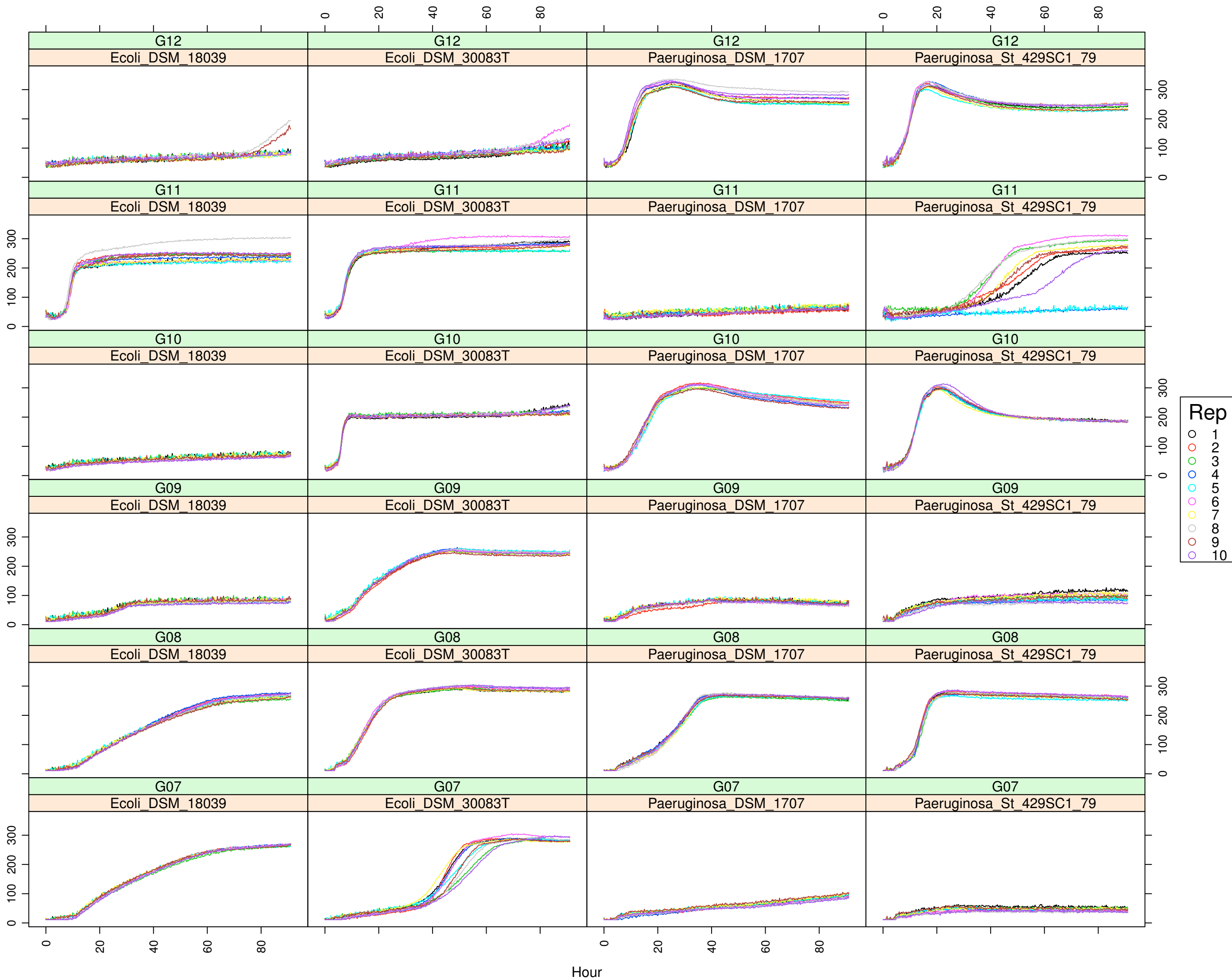

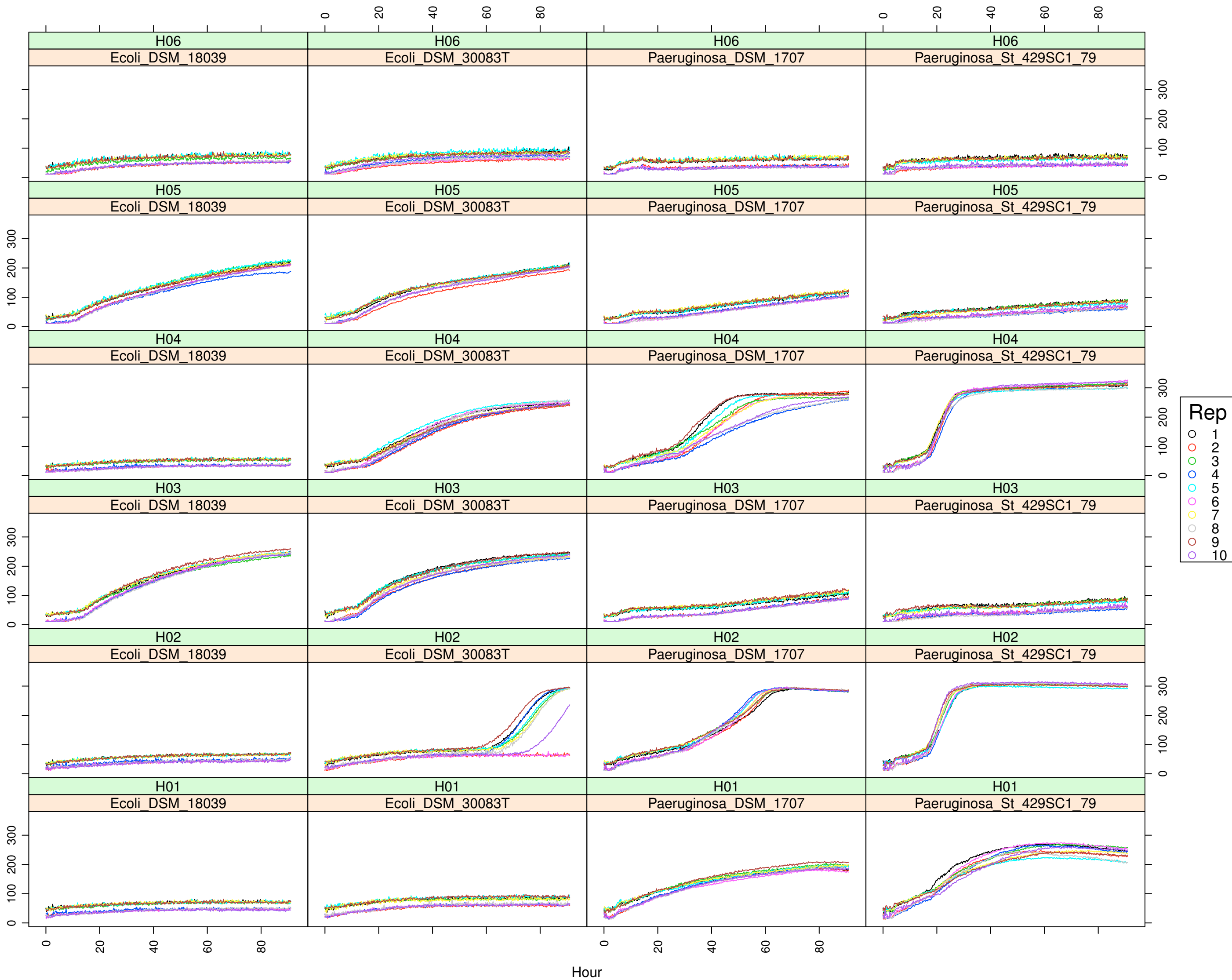

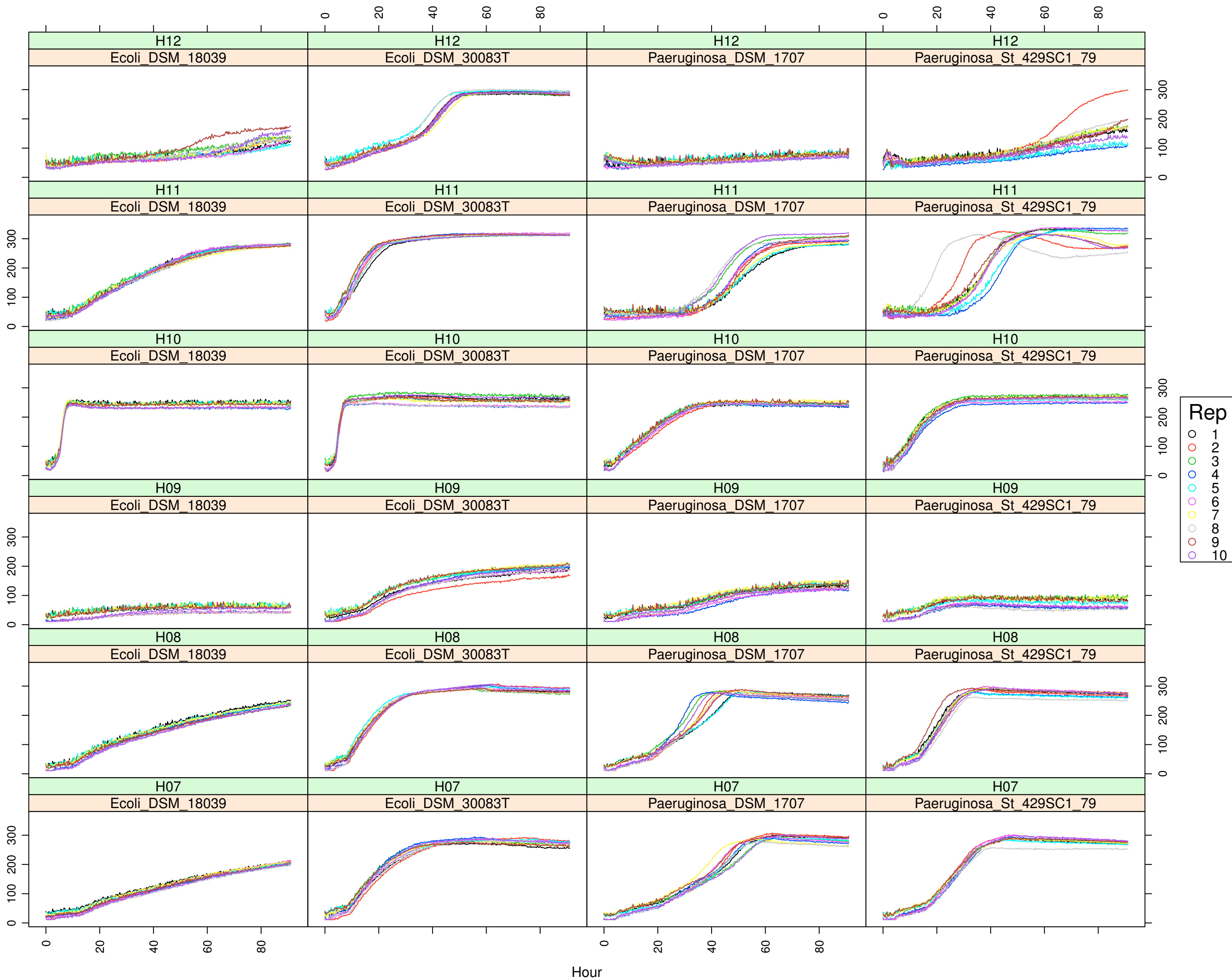

Supplement: File S4 — Plots of all respiration curves from dataset 1 in PDF format. (PDF) [file pone.0034846.s004.pdf]

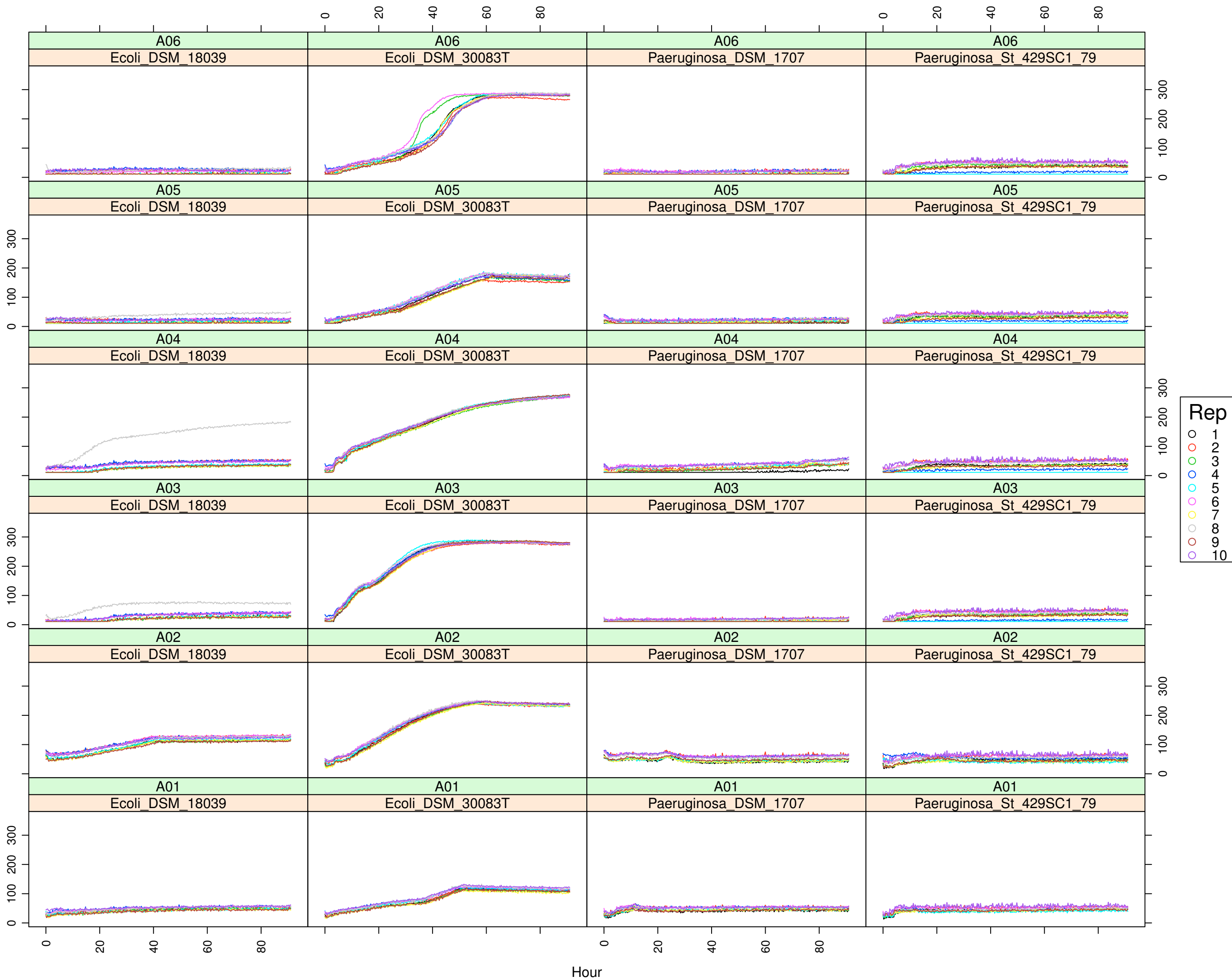

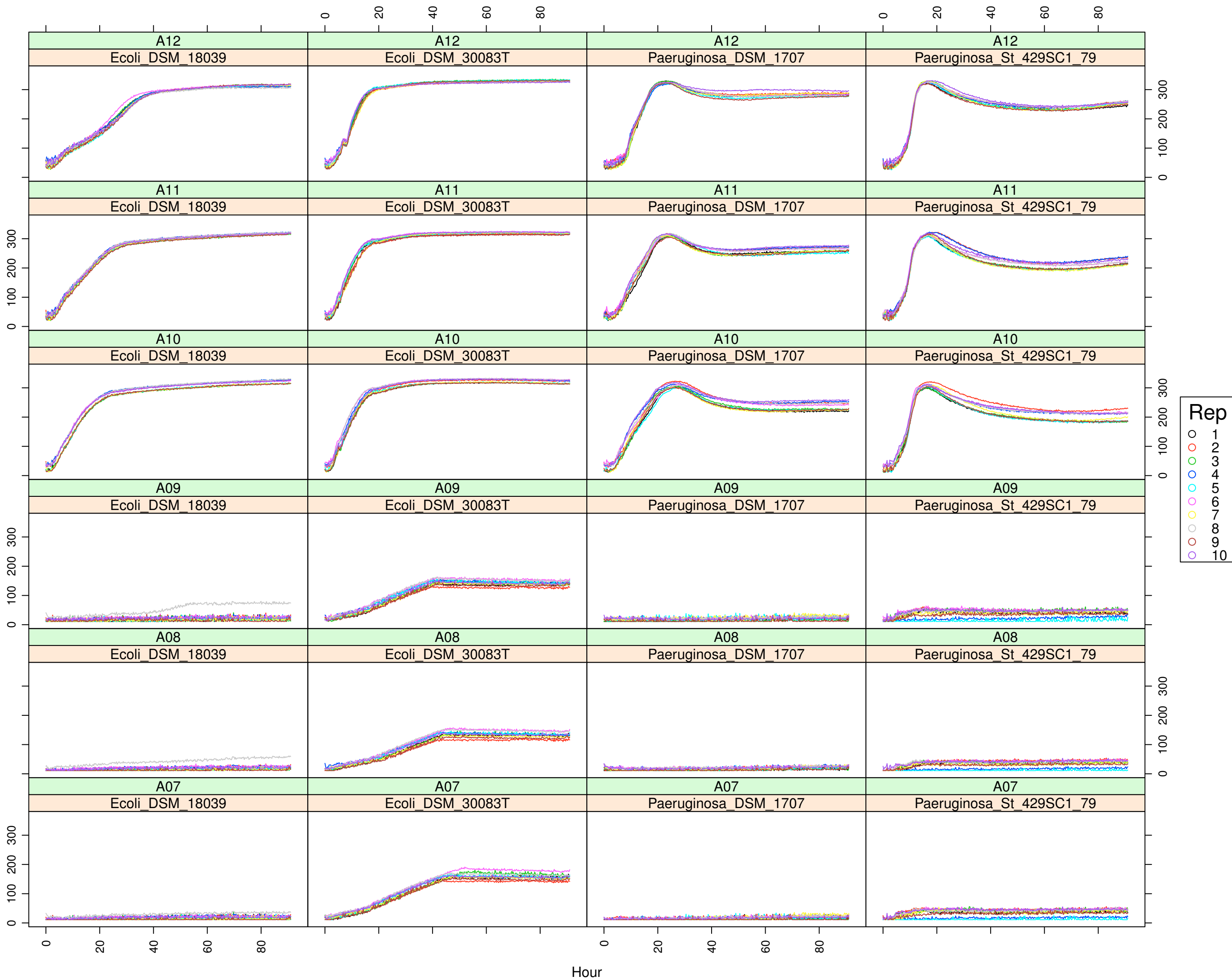

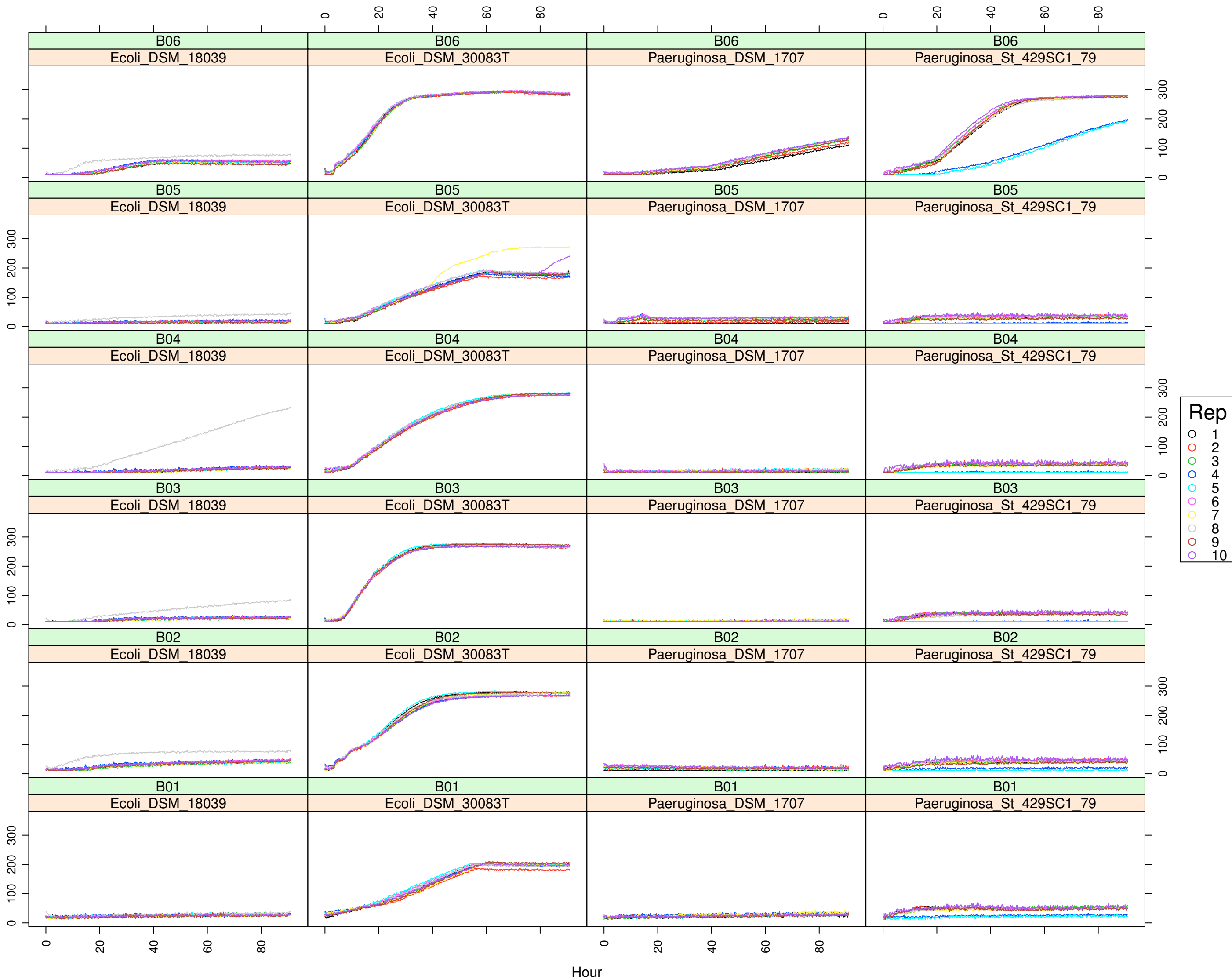

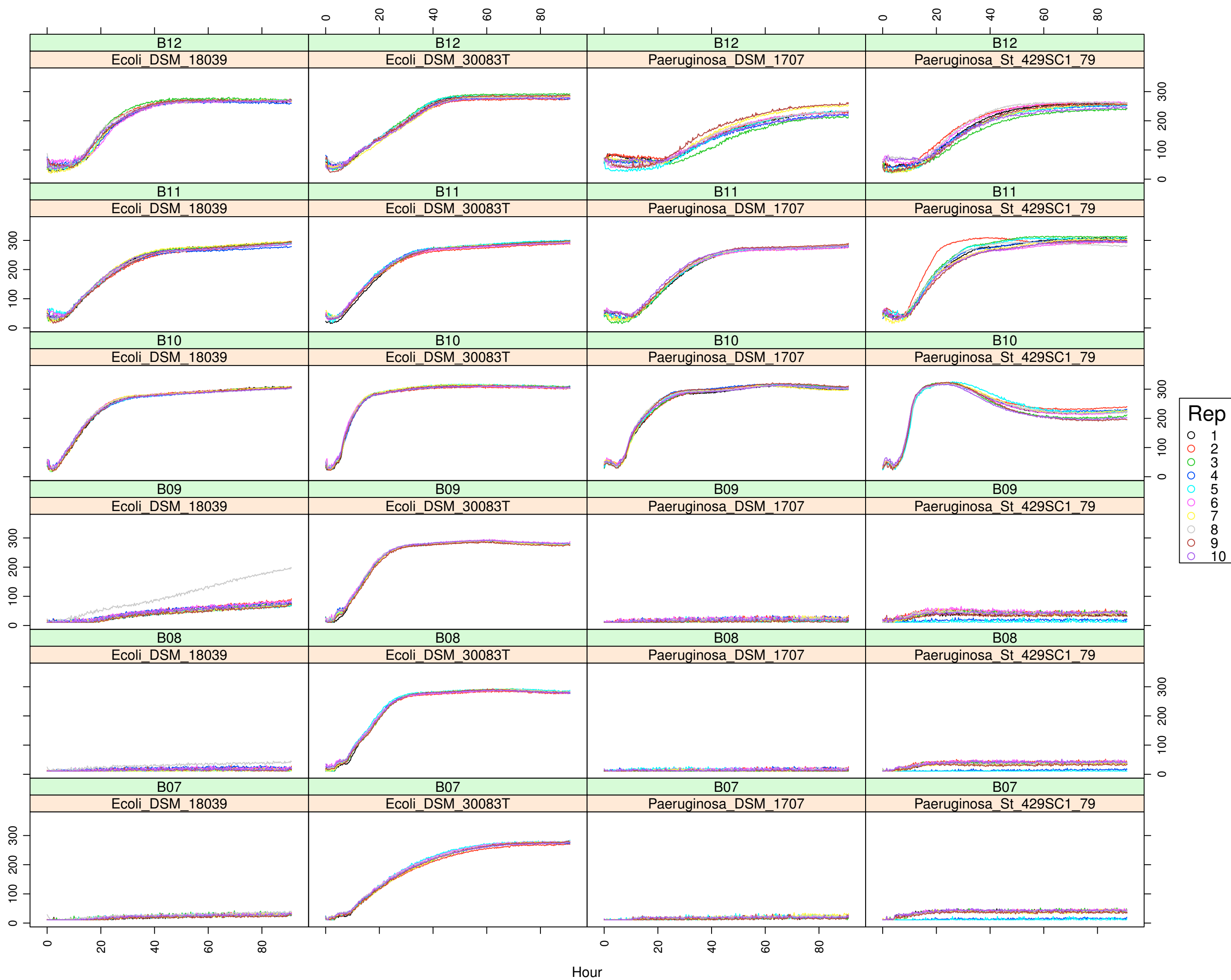

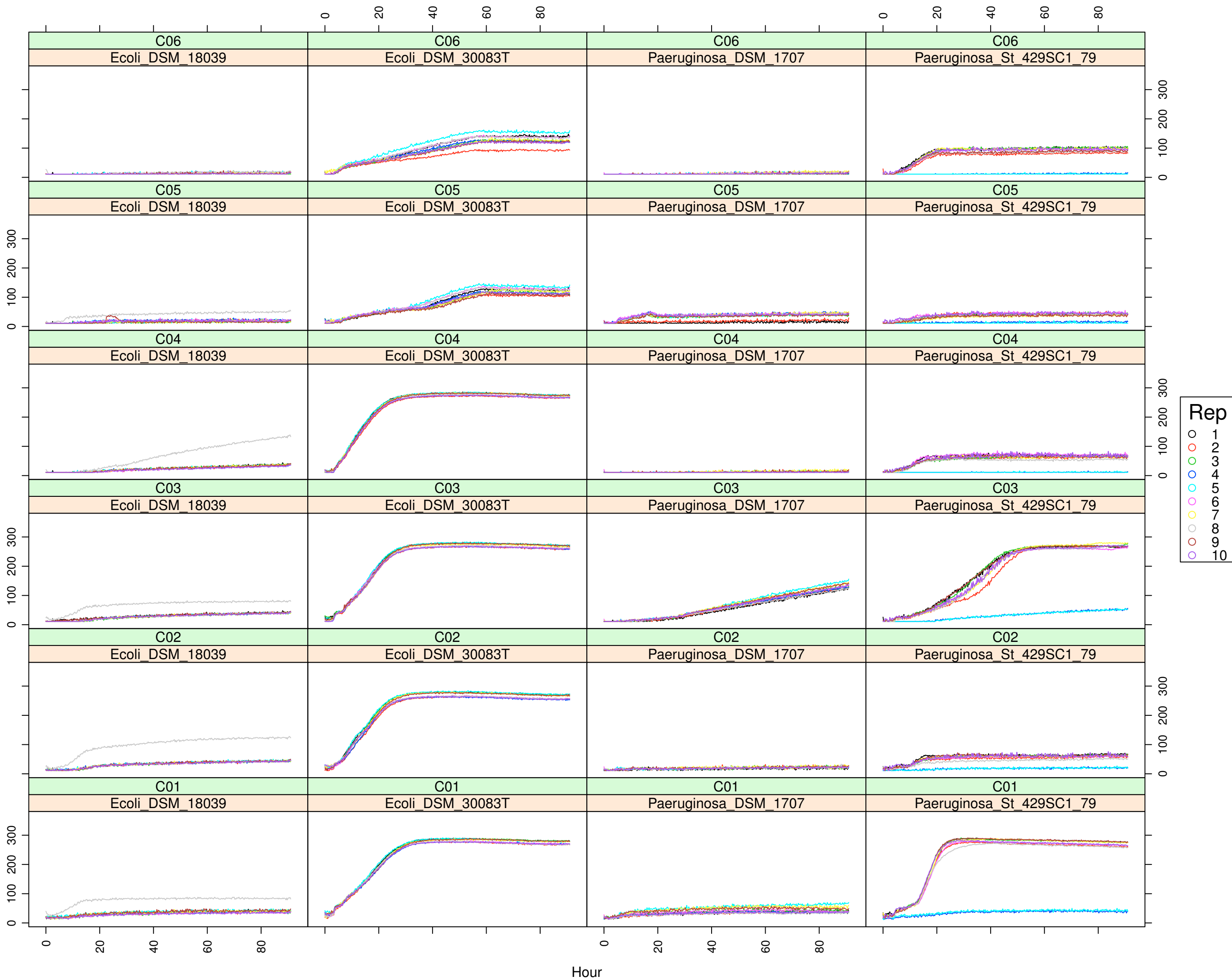

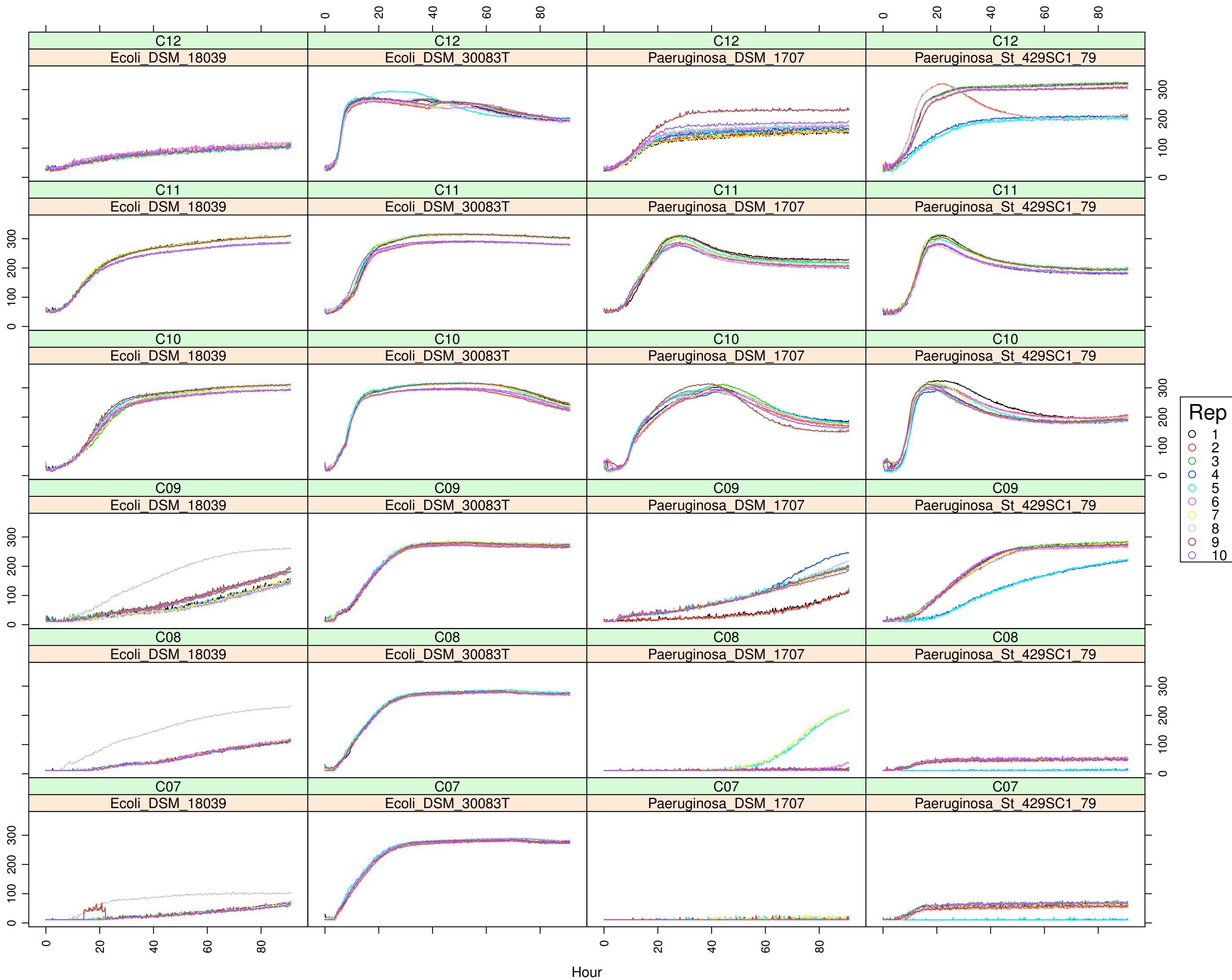

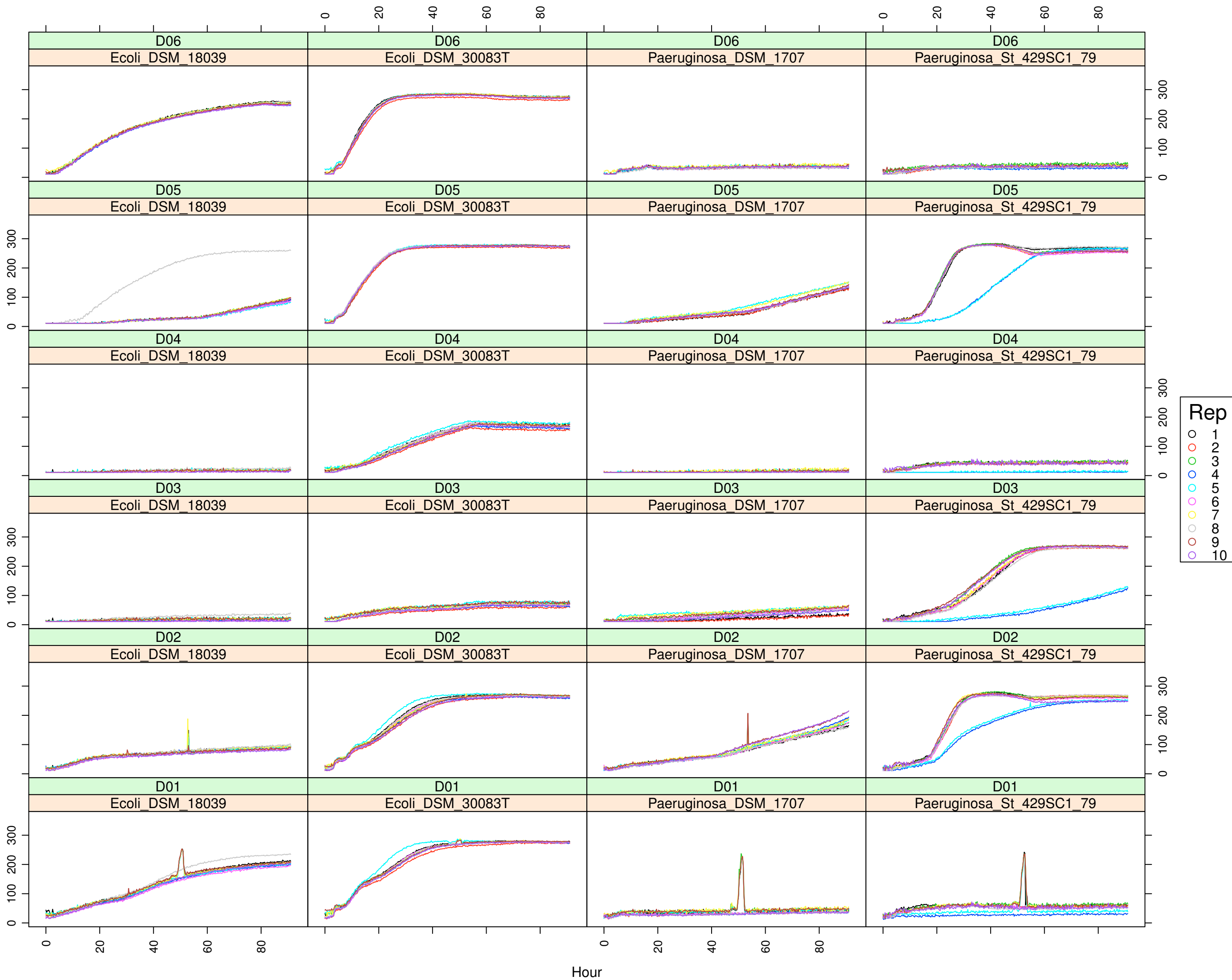

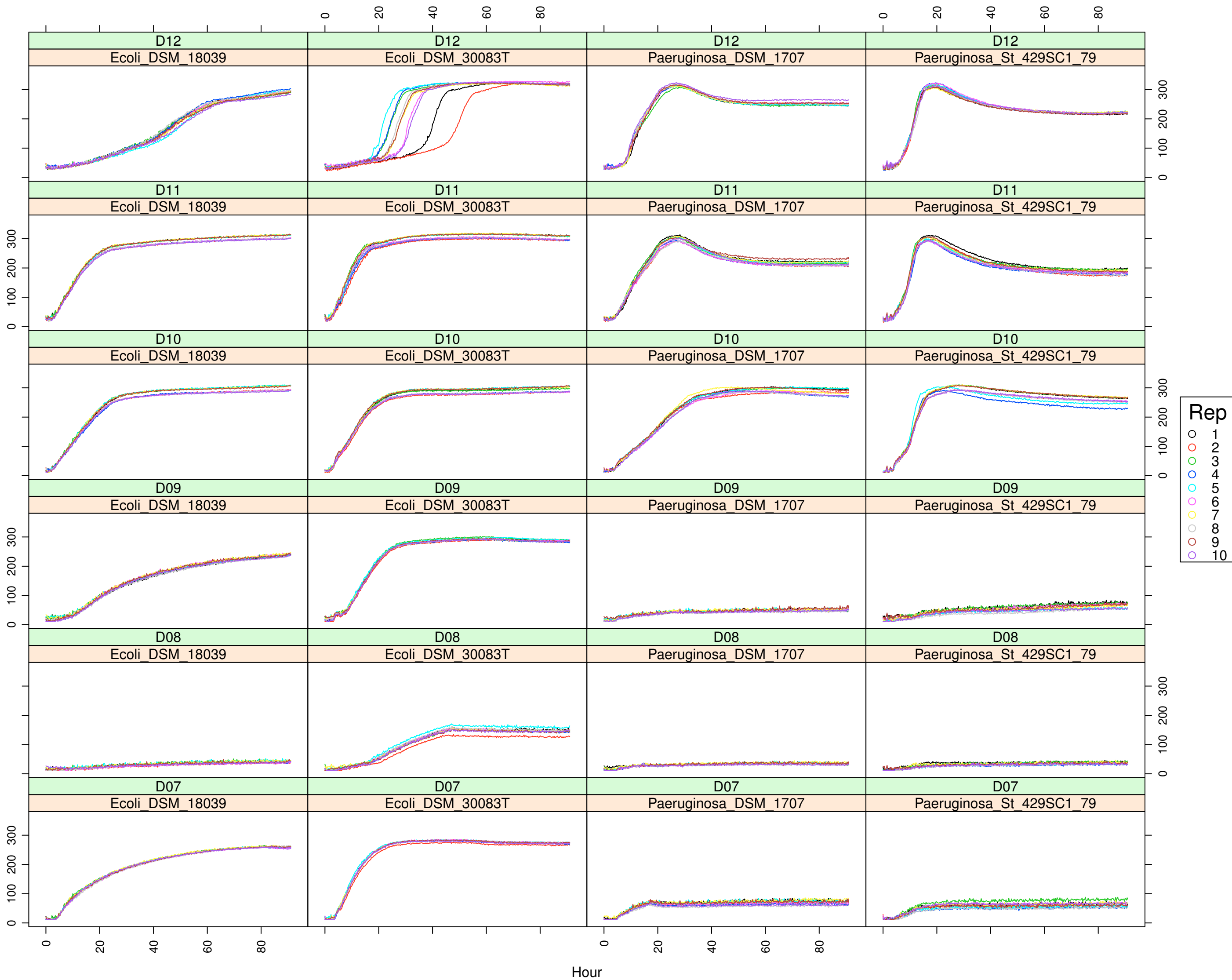

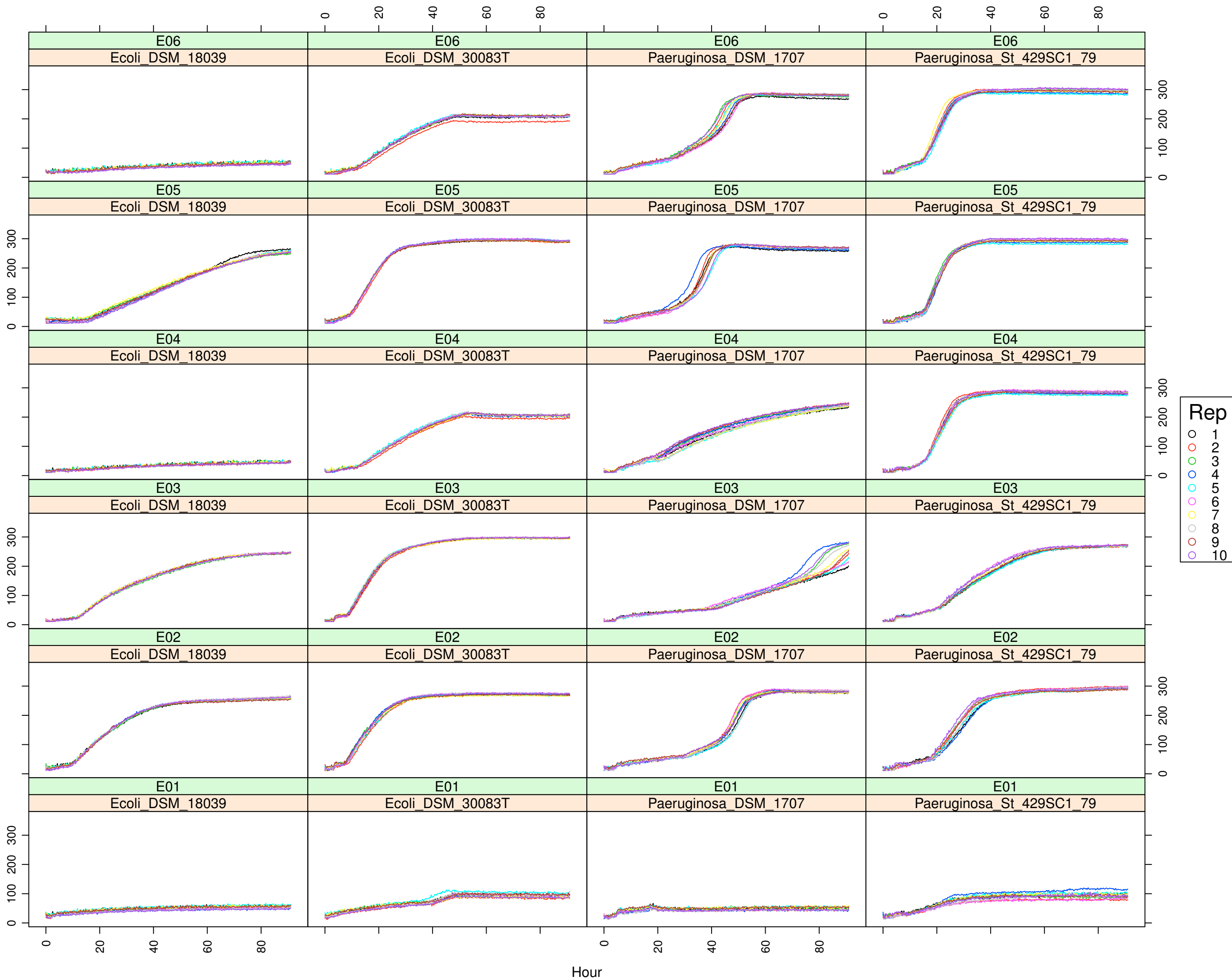

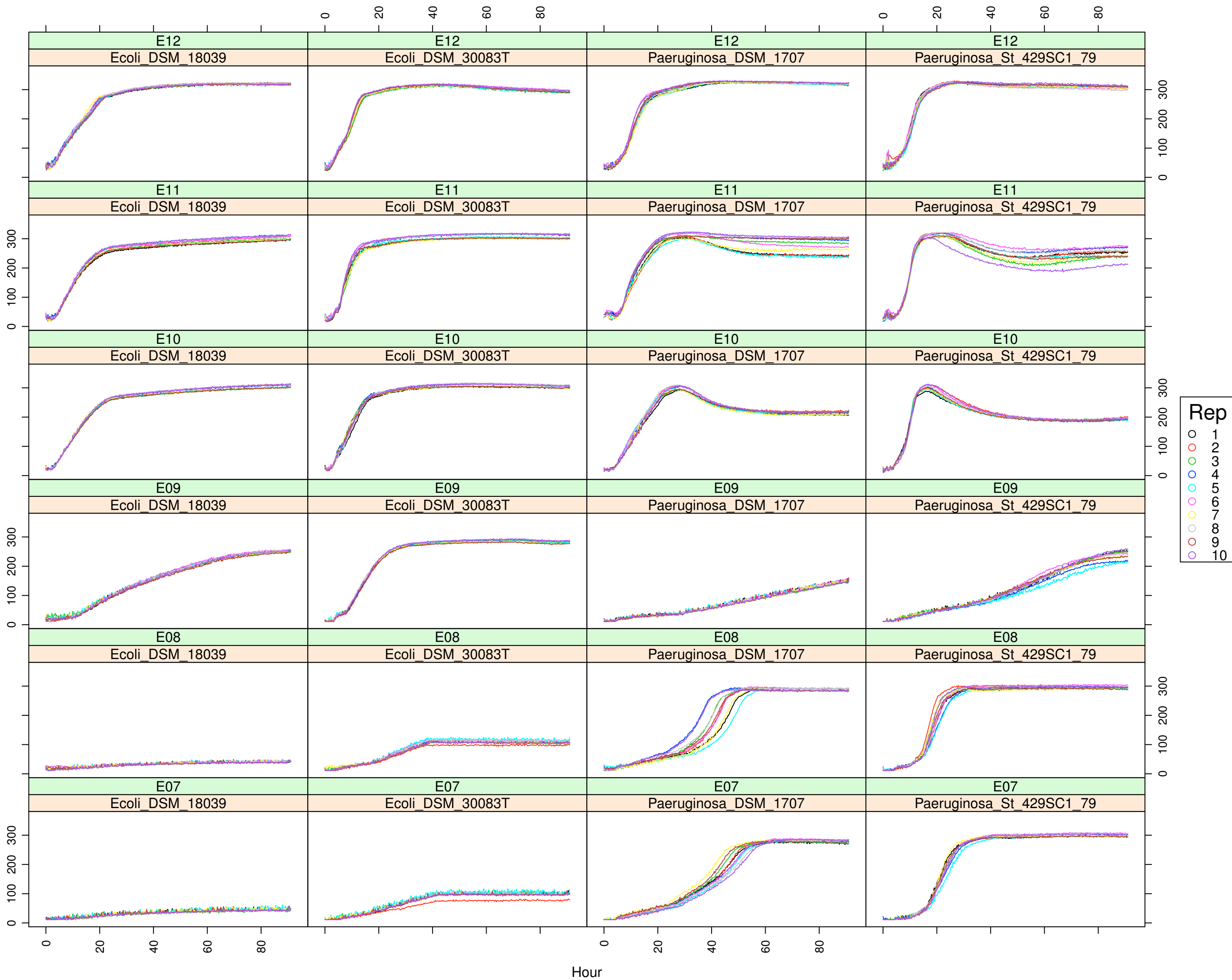

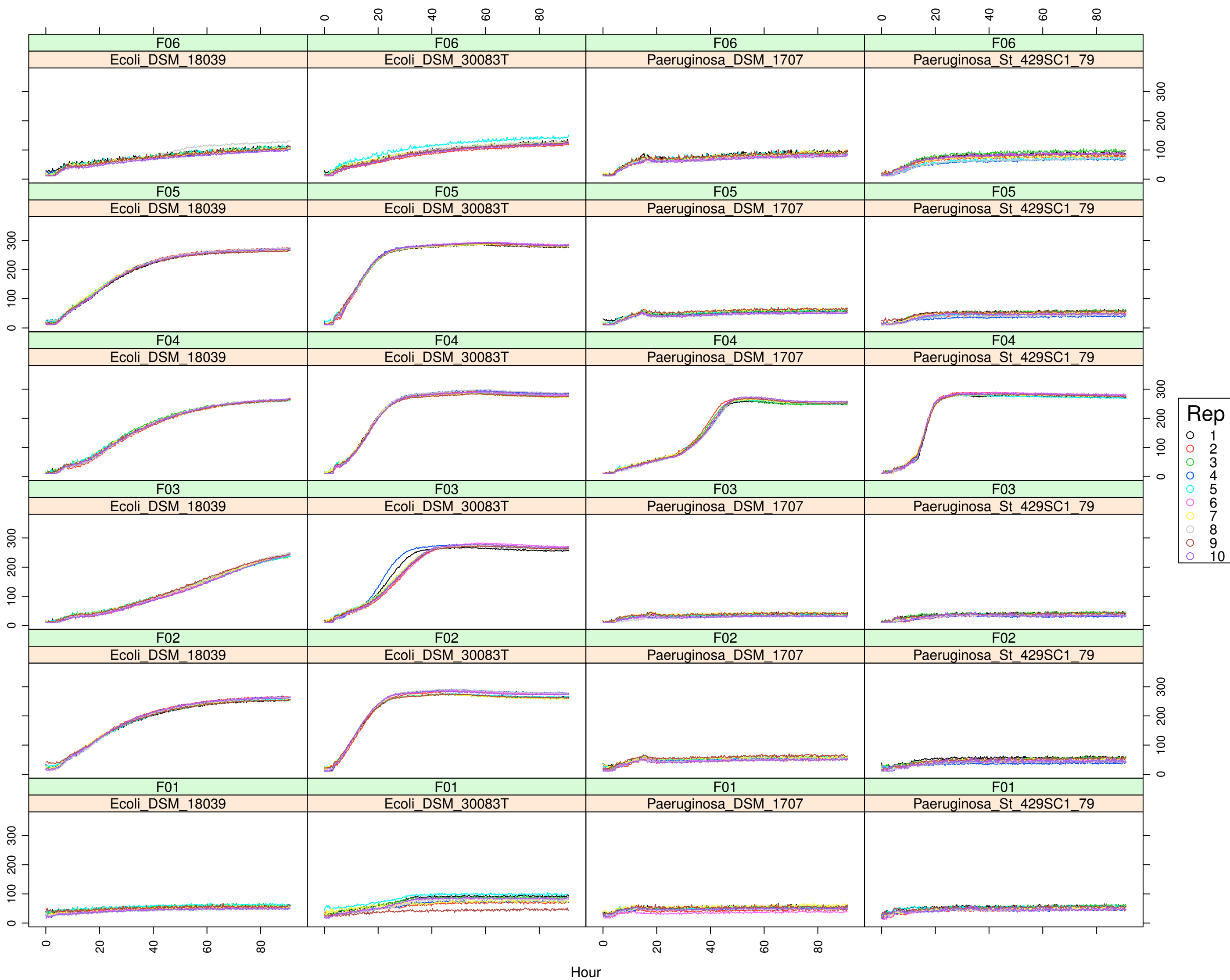

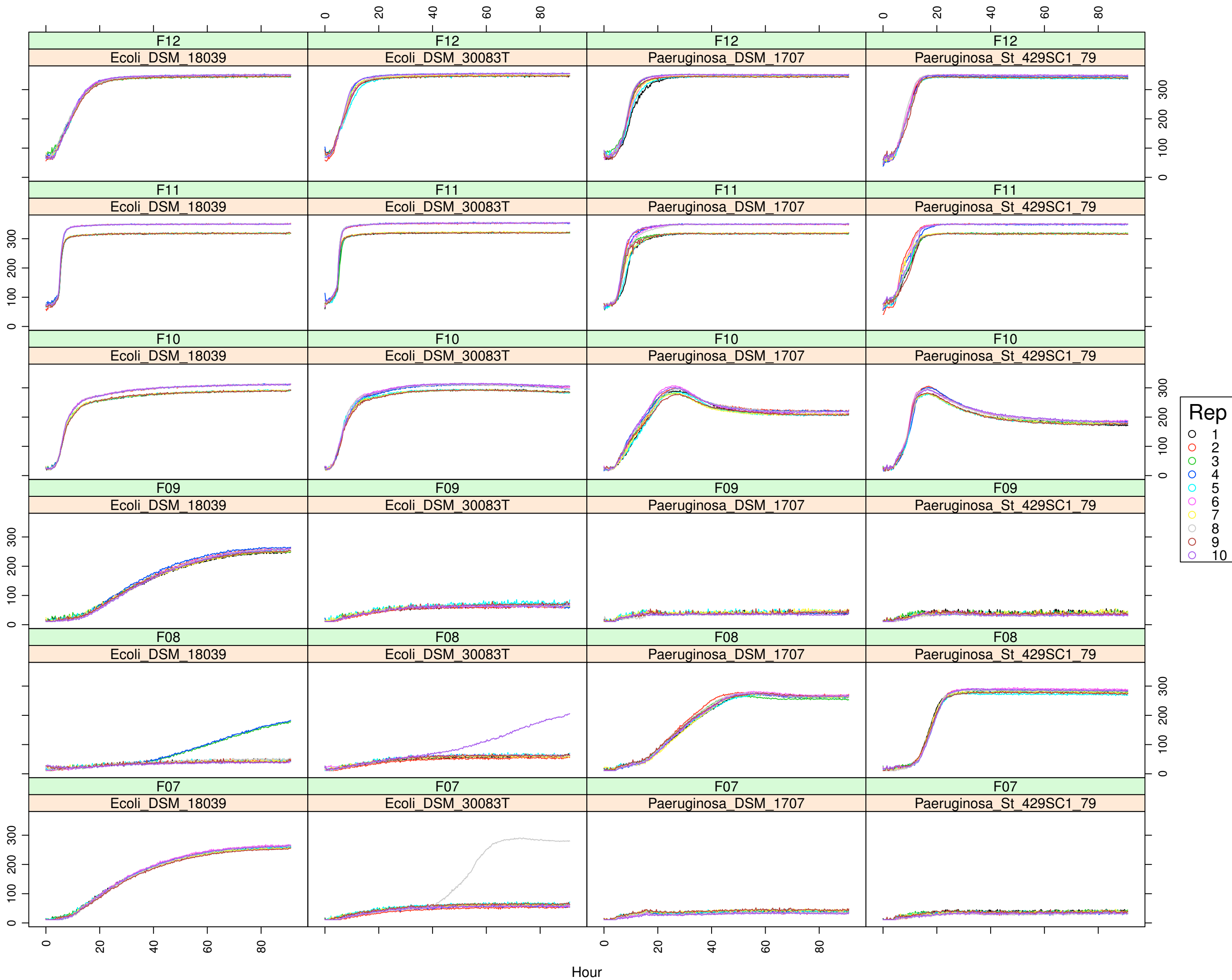

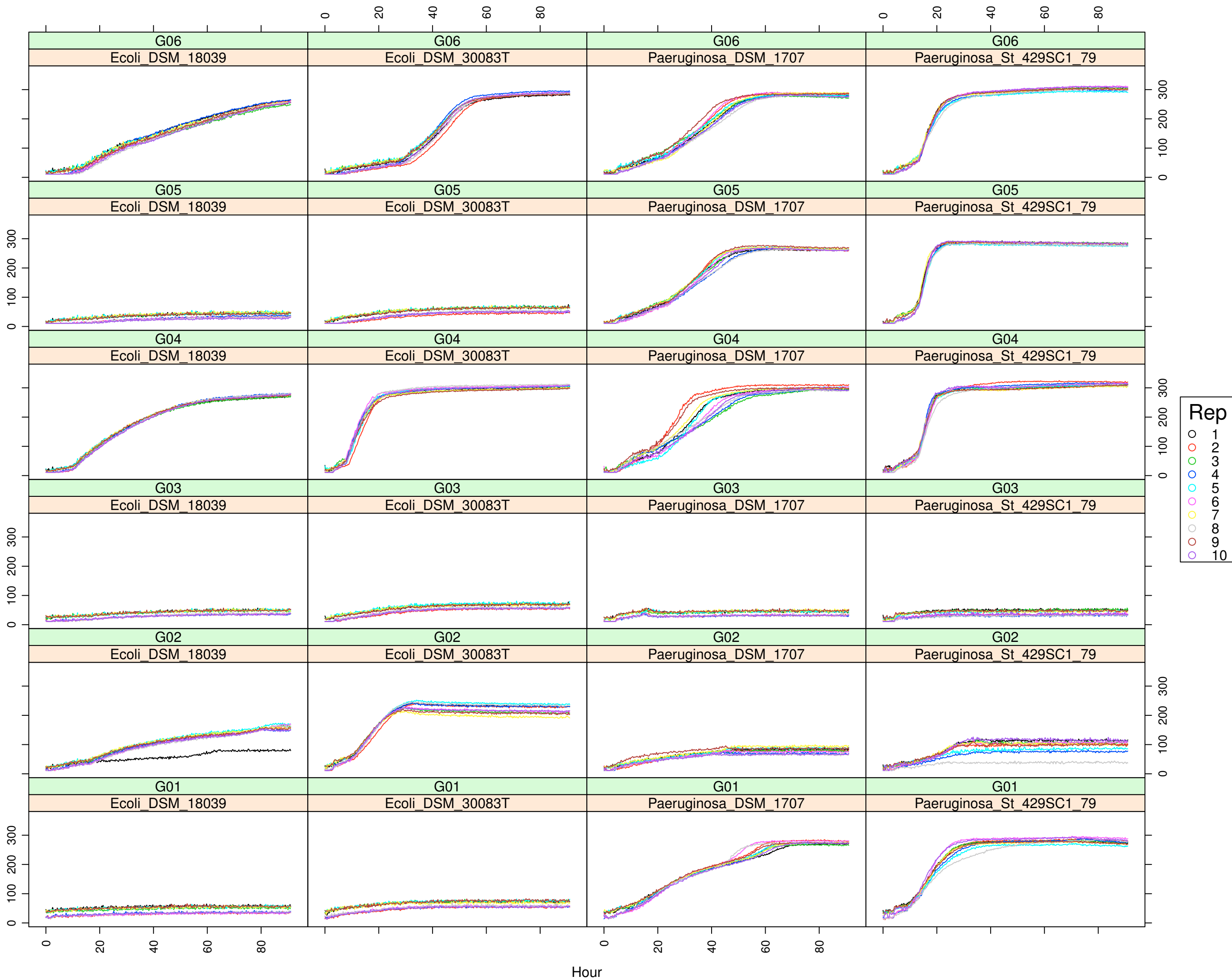

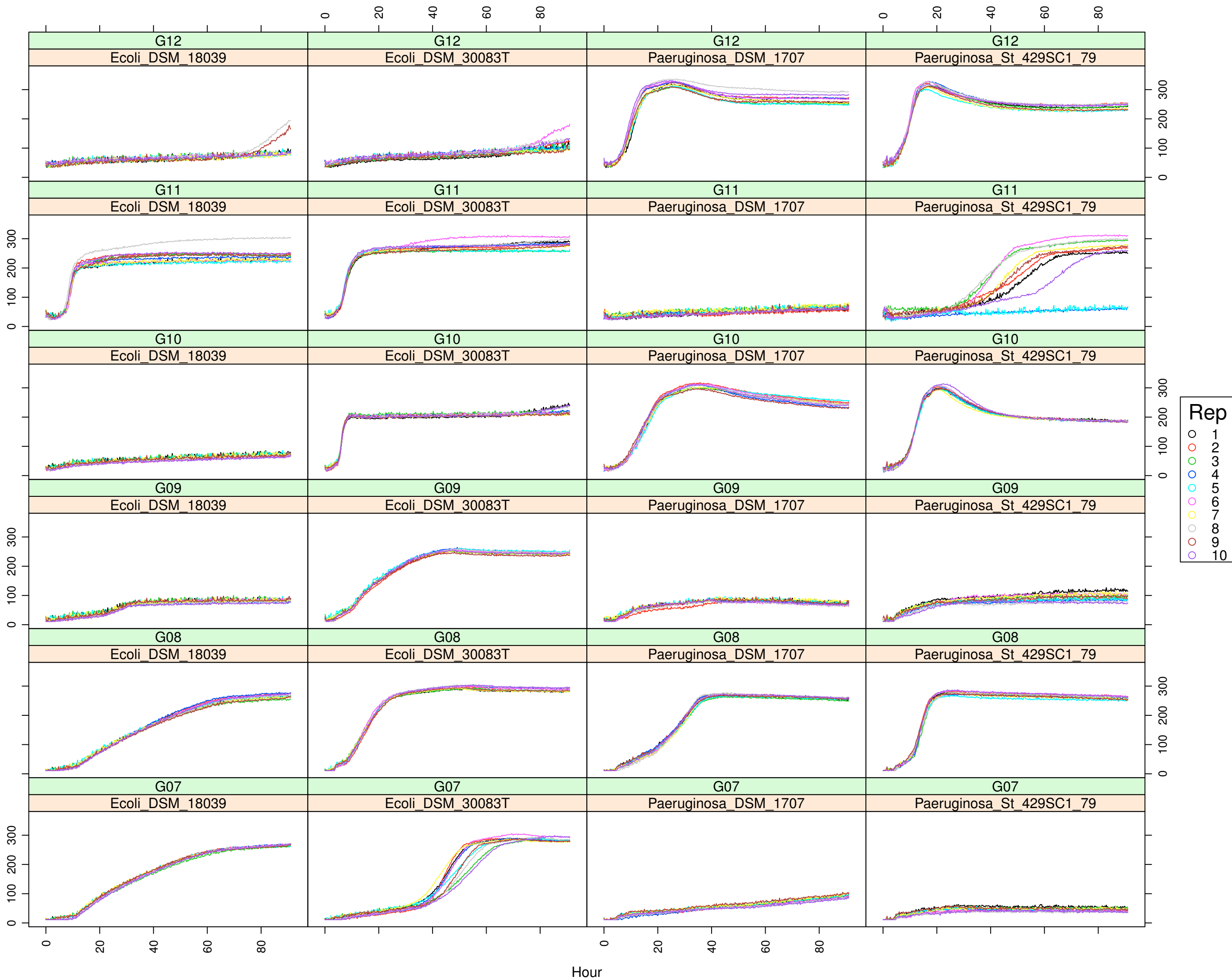

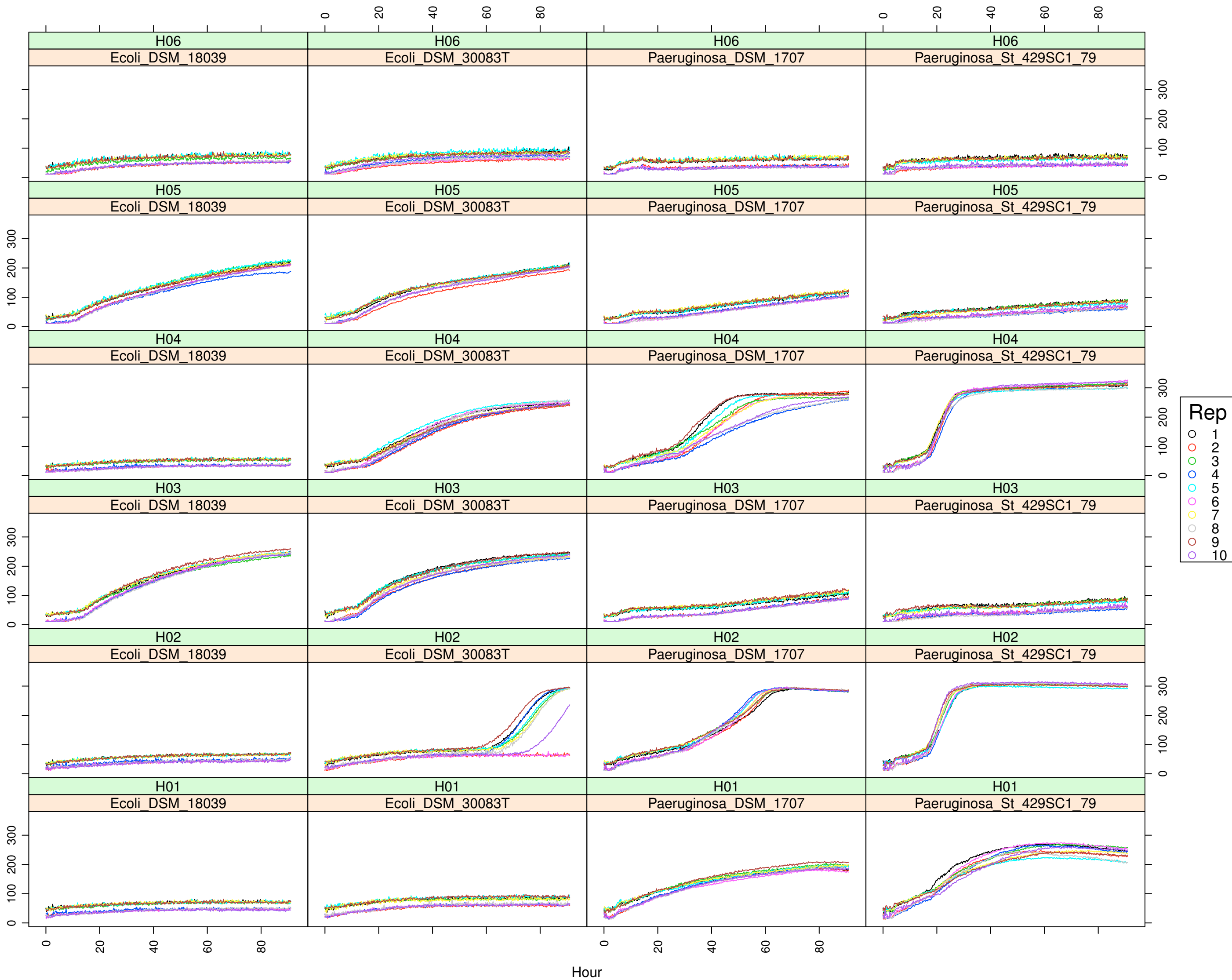

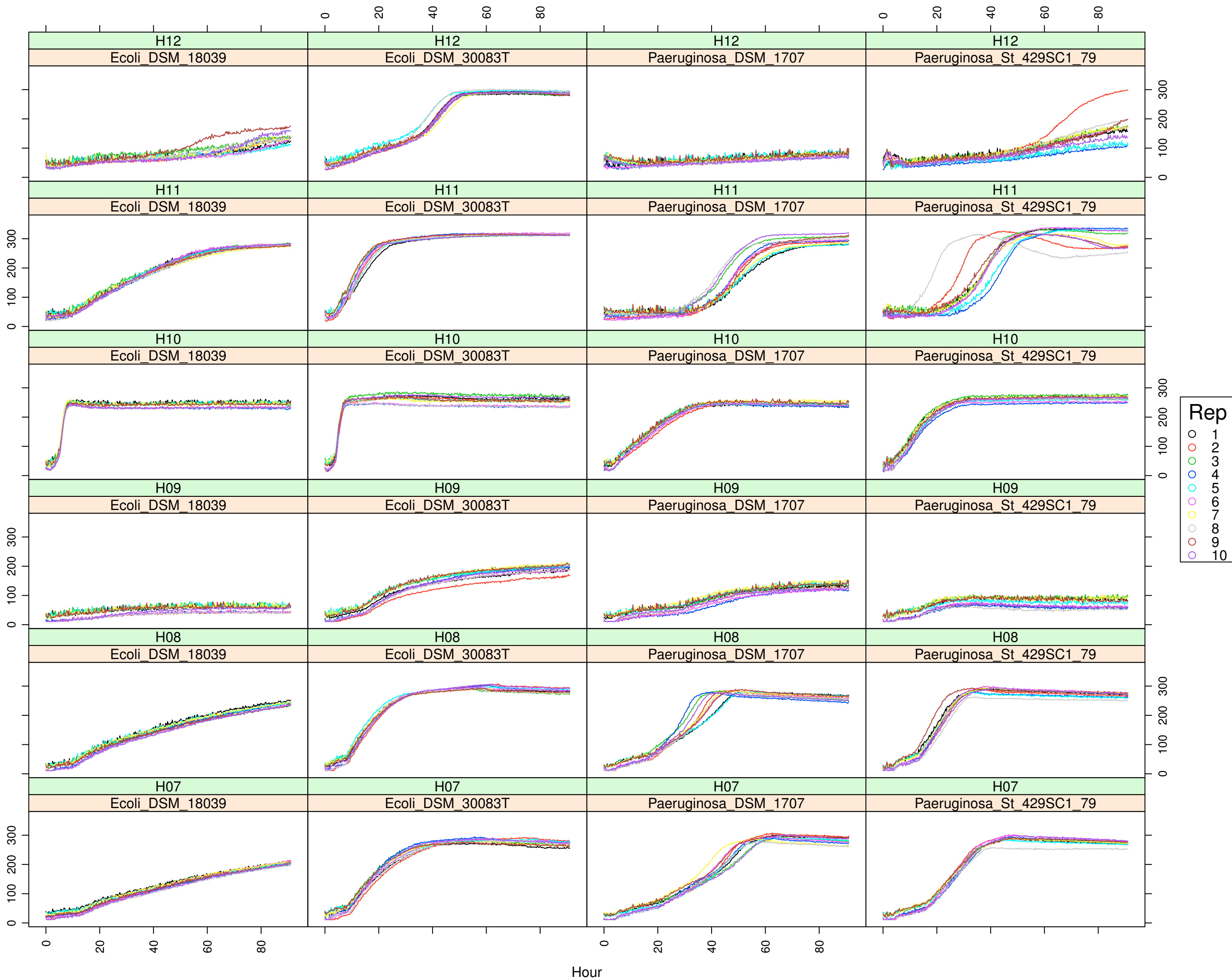

Supplement: File S5 — Plots of all respiration curves from dataset 2 in PDF format. (PDF) [file pone.0034846.s005.pdf]

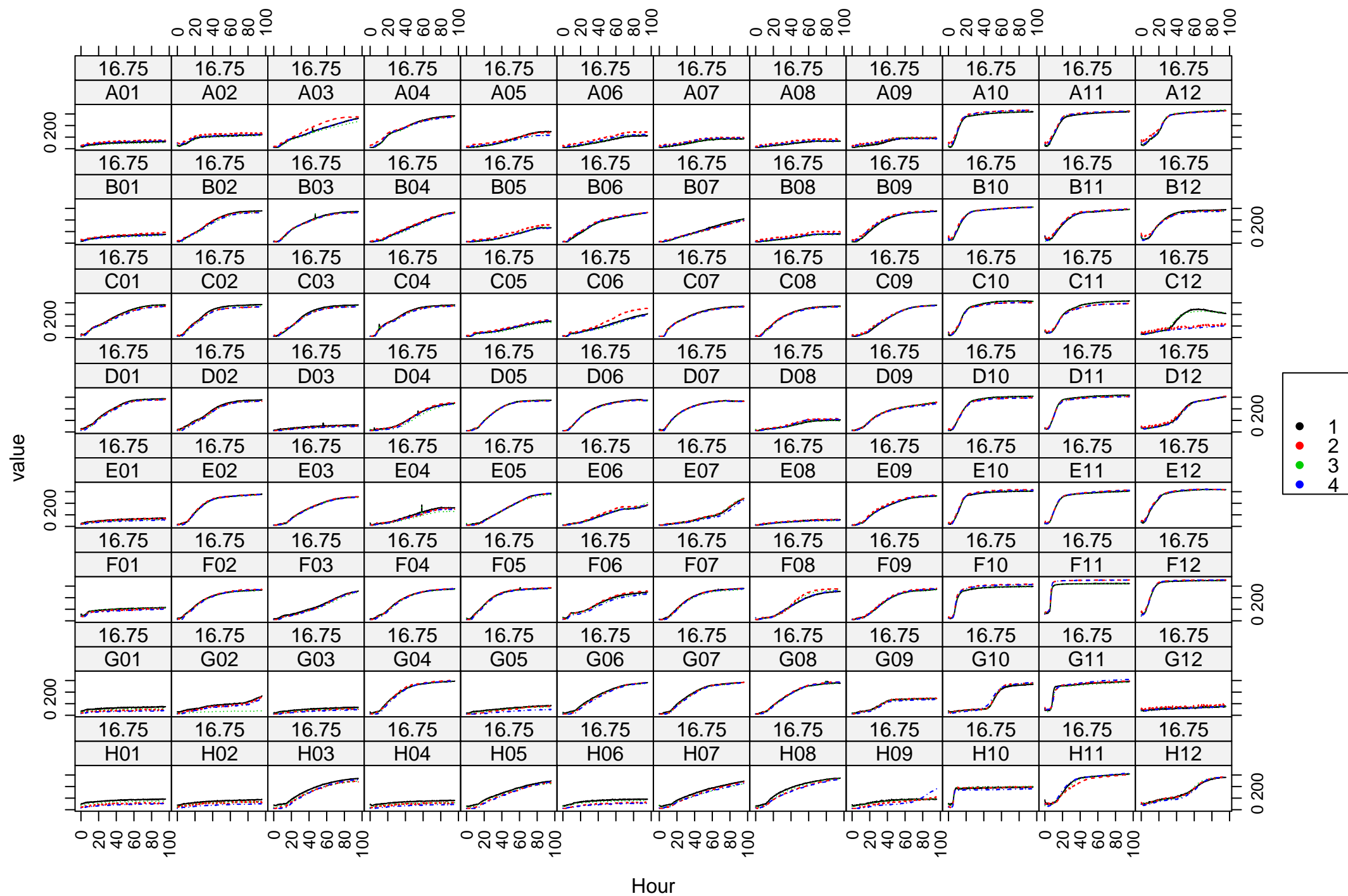

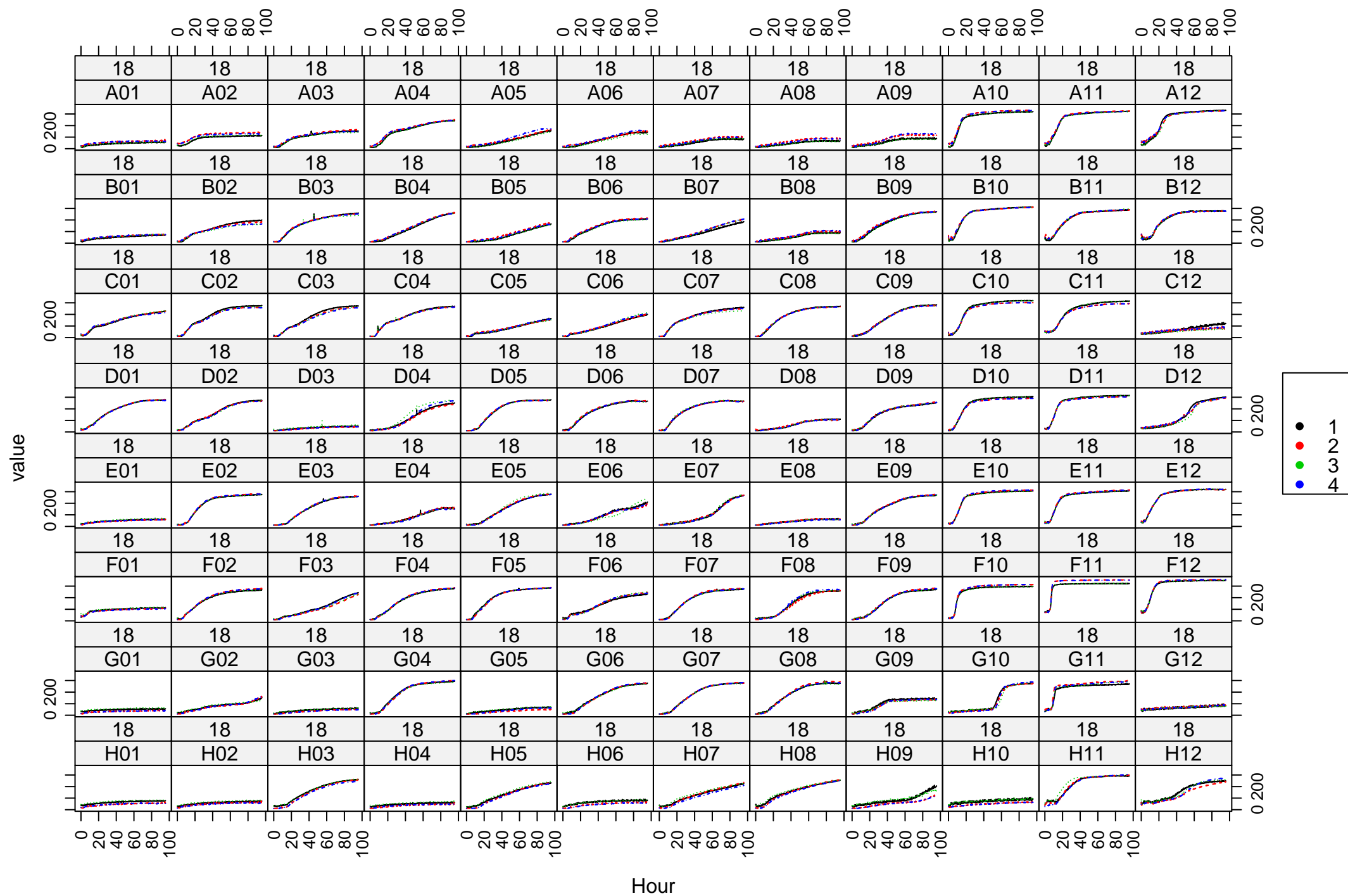

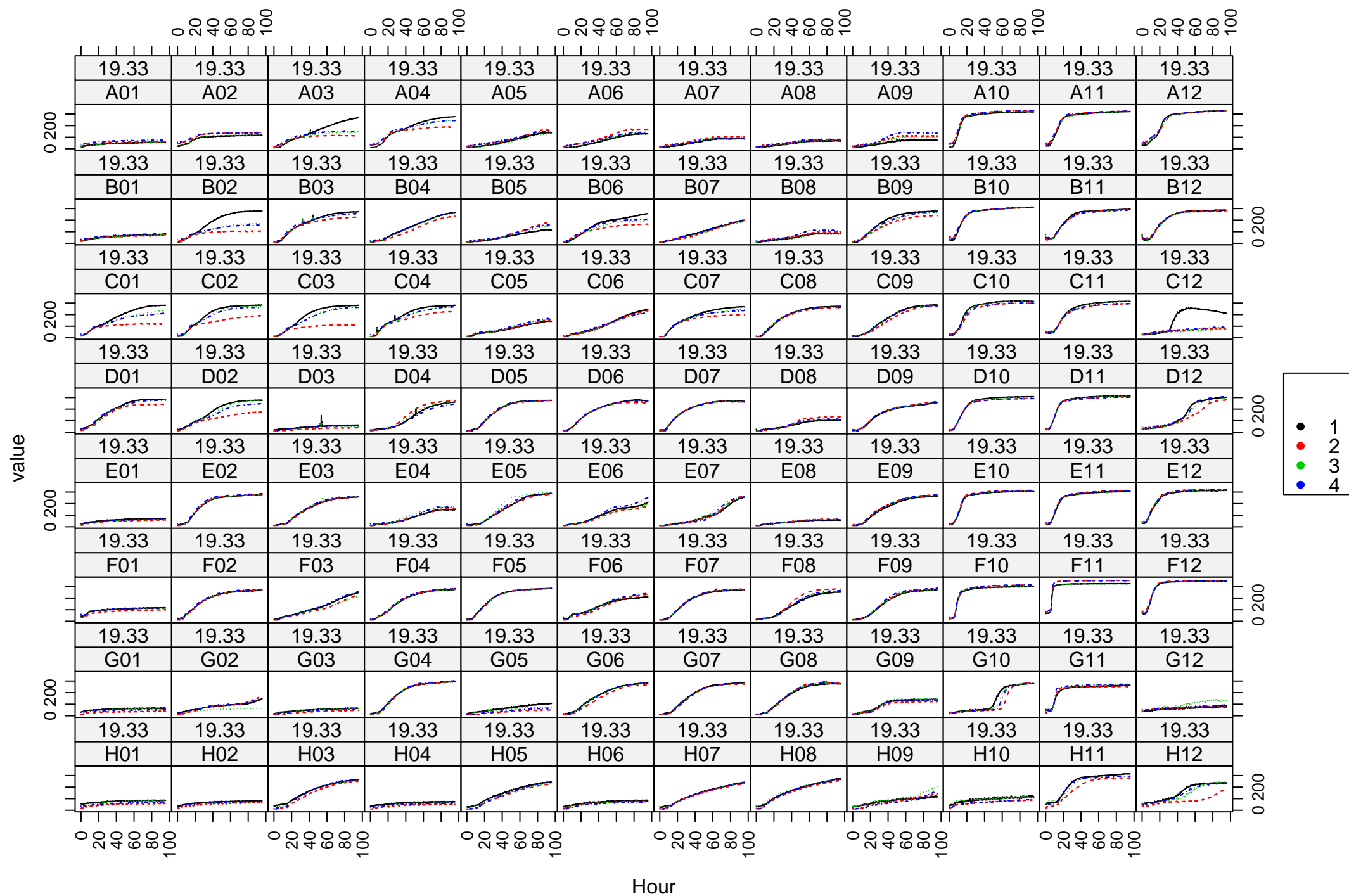

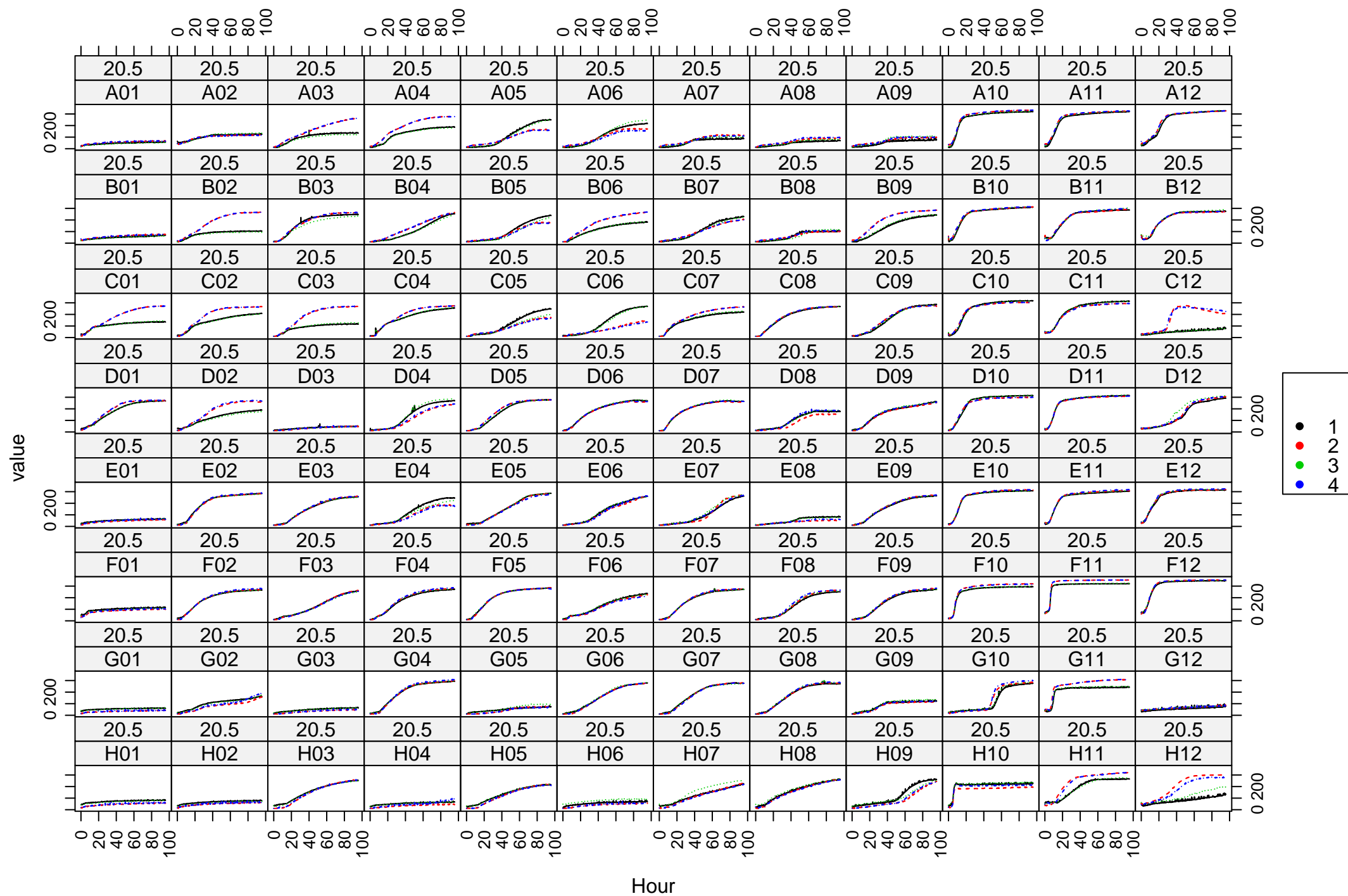

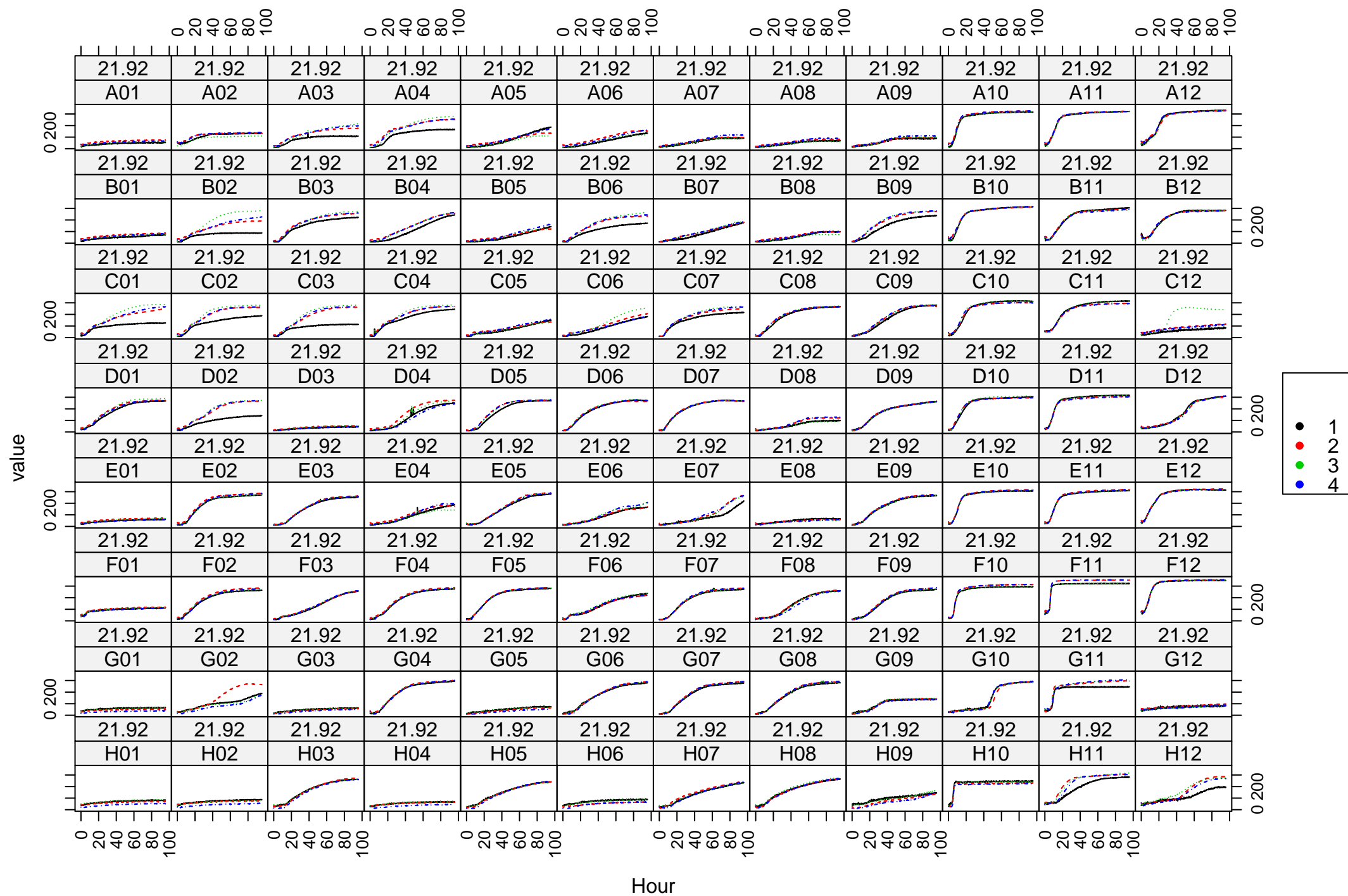

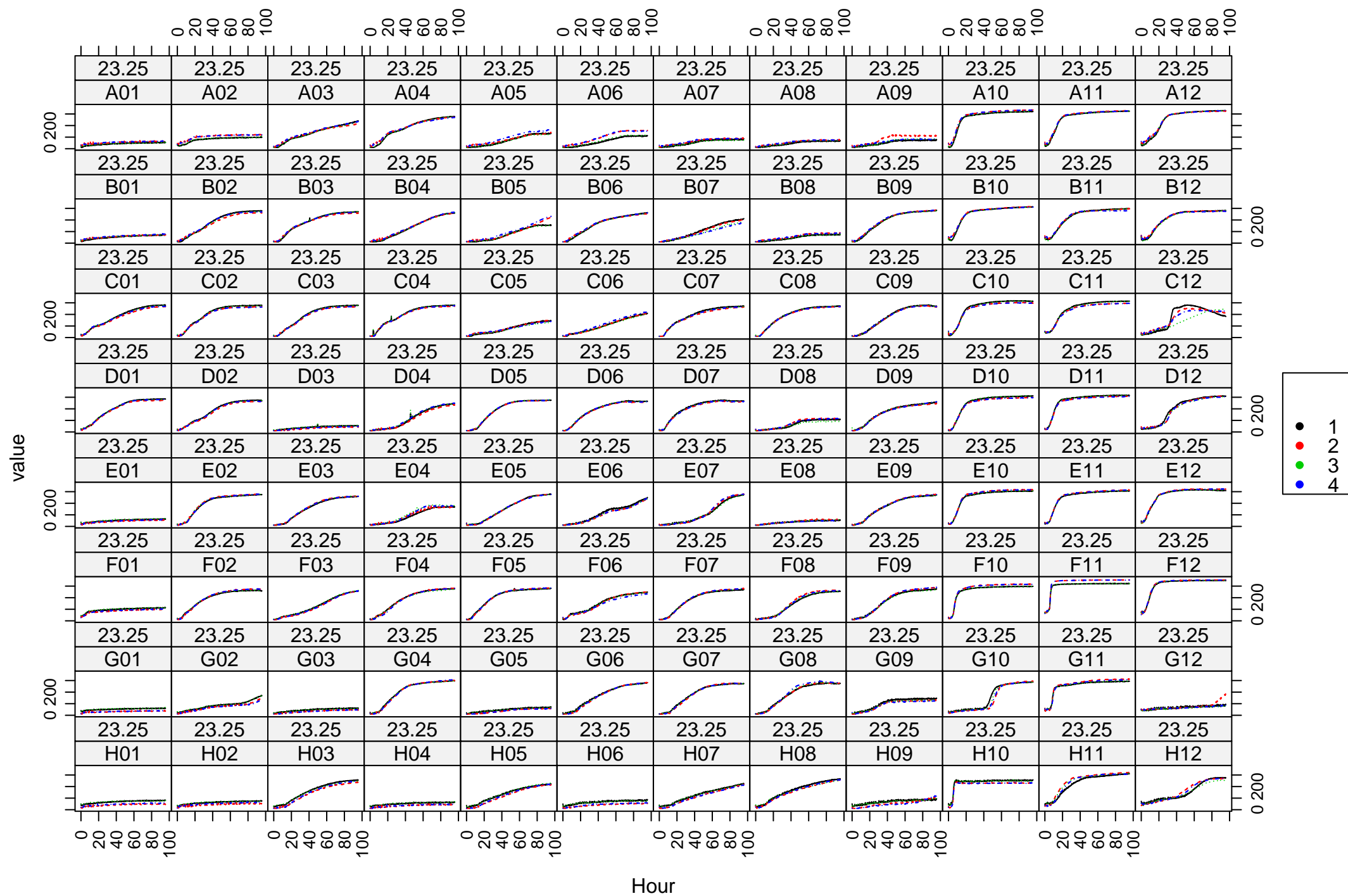

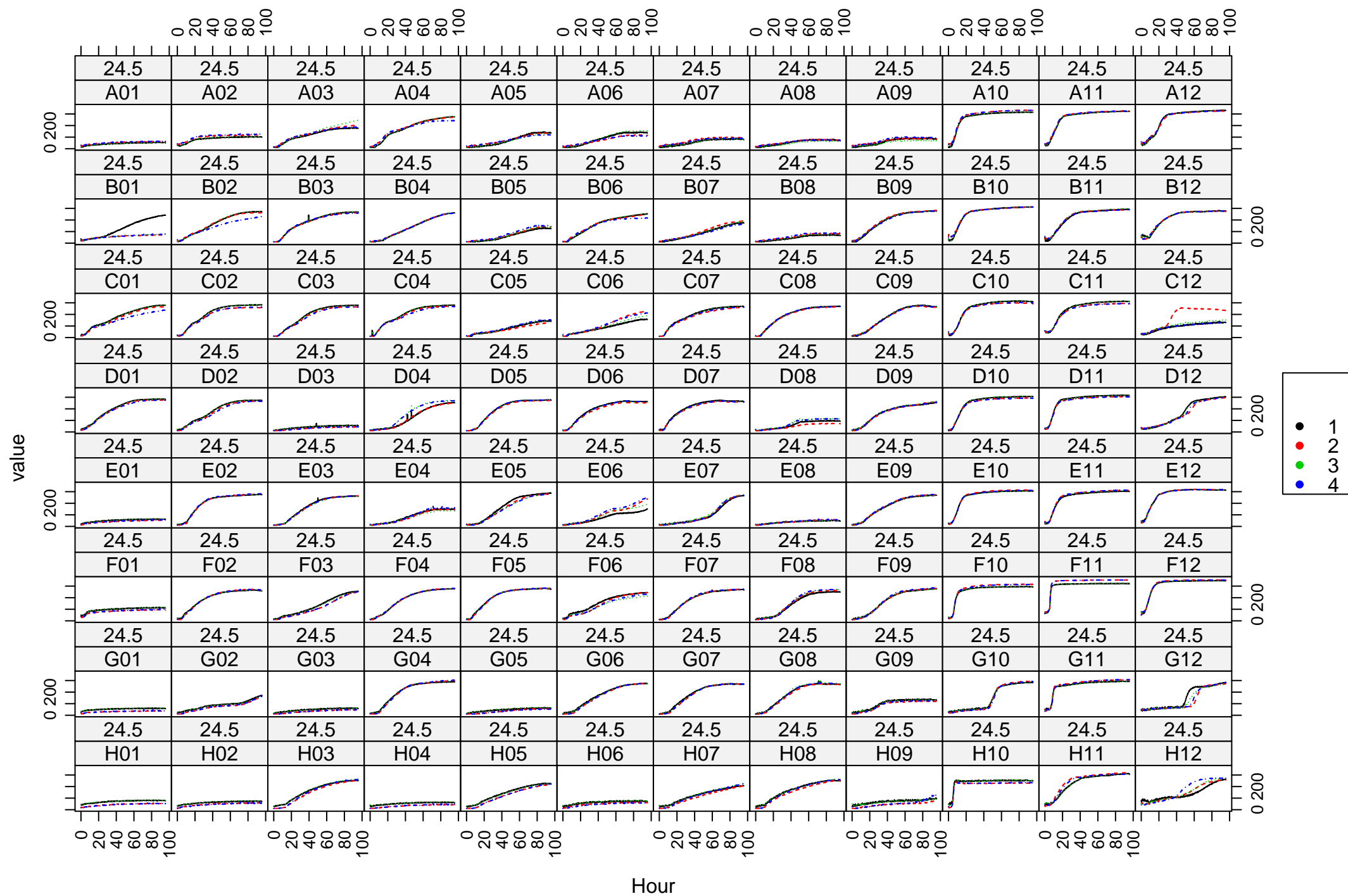

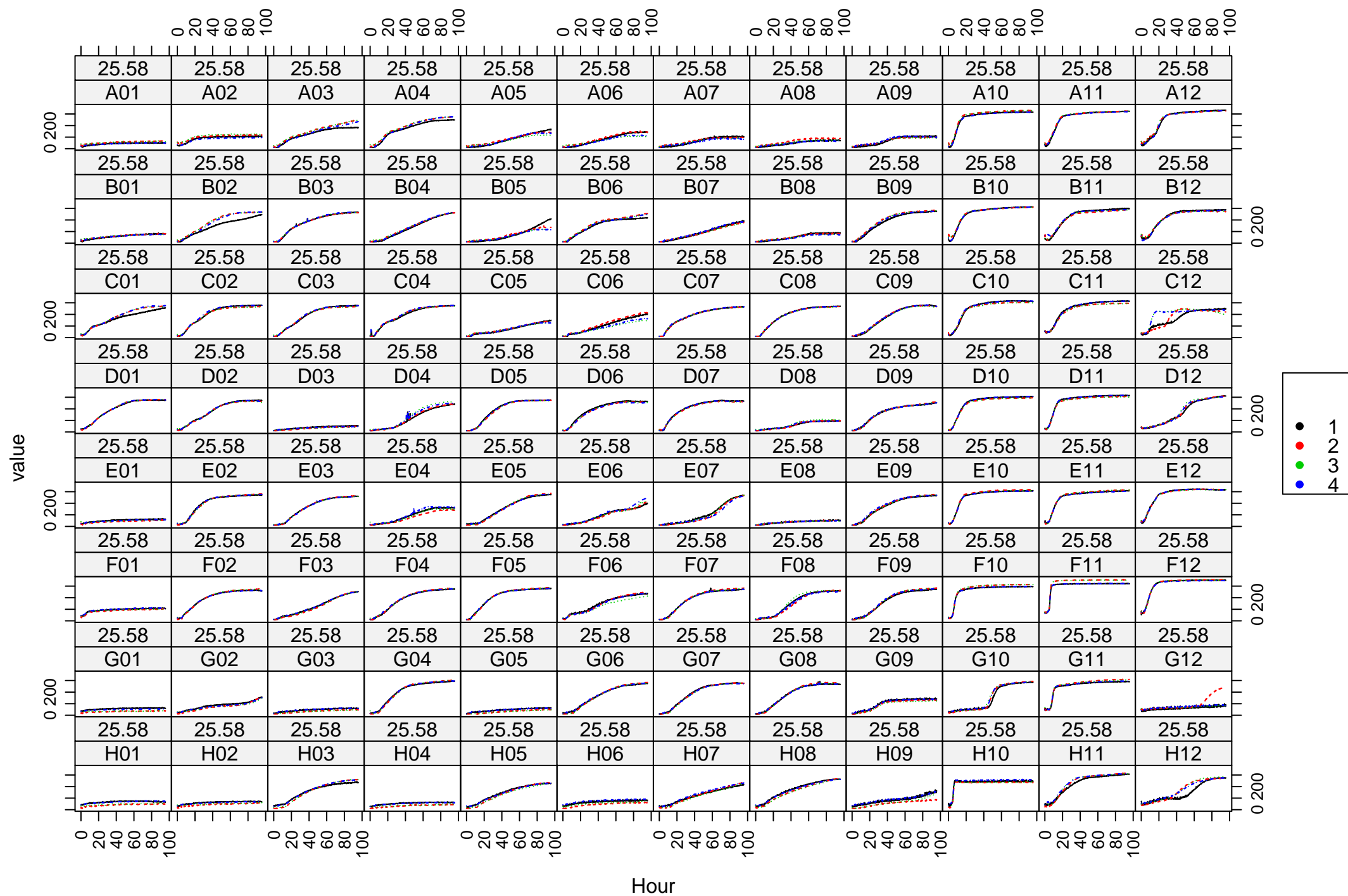

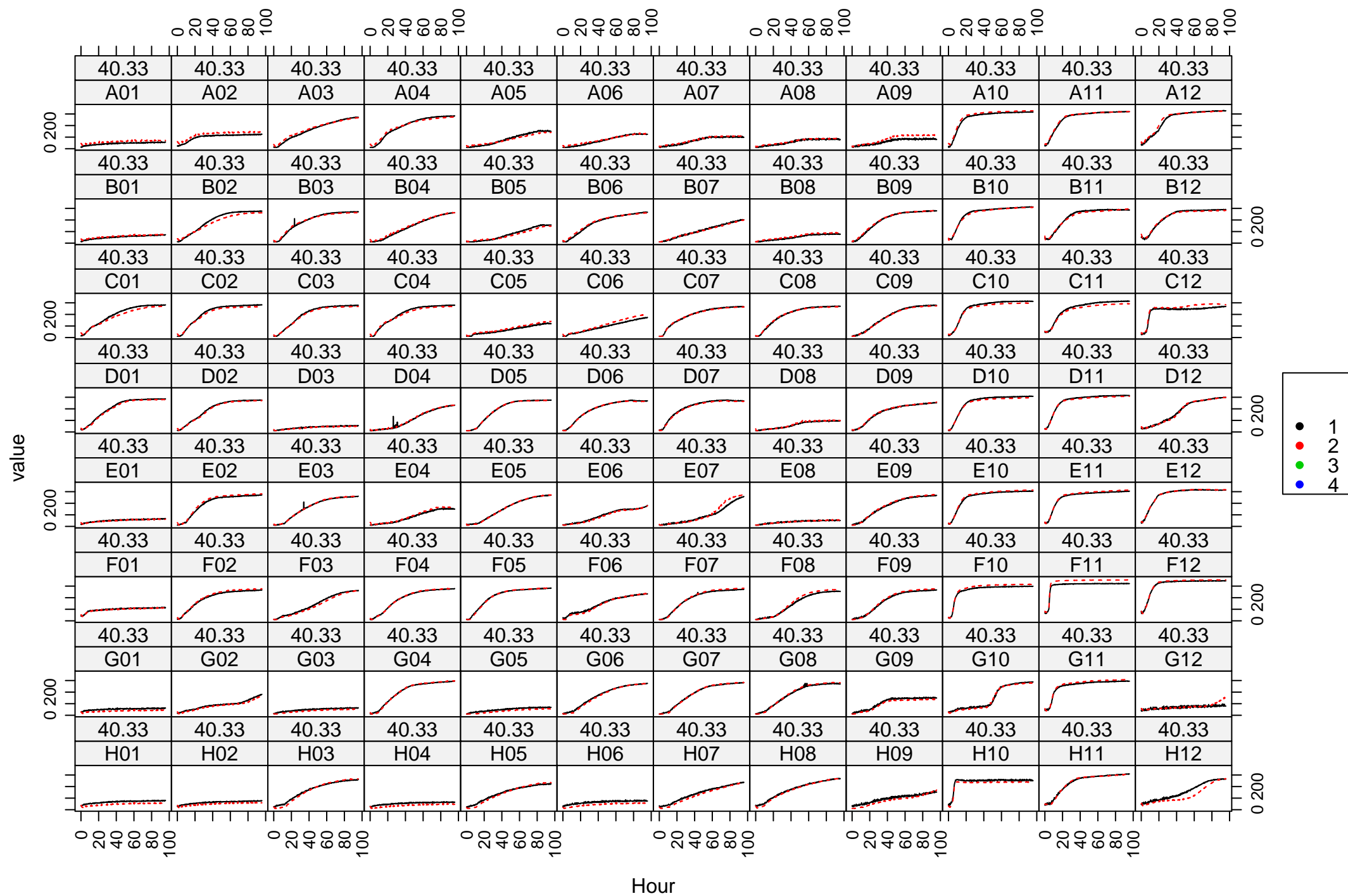

Supplement: File S6 — Plots of all respiration curves from dataset 3 in PDF format. (PDF) [file pone.0034846.s006.pdf]

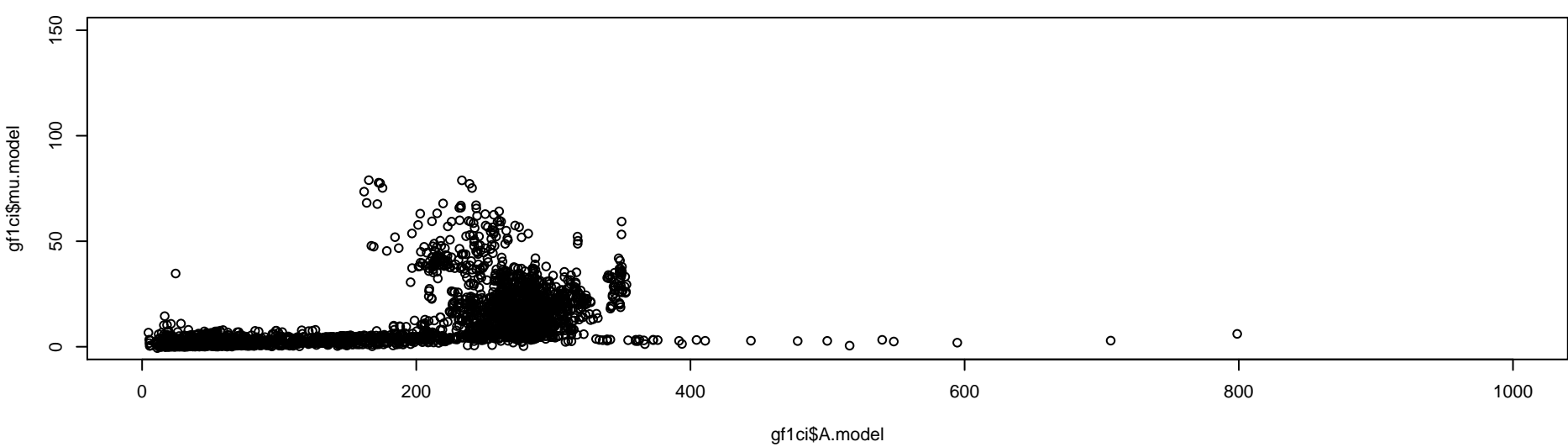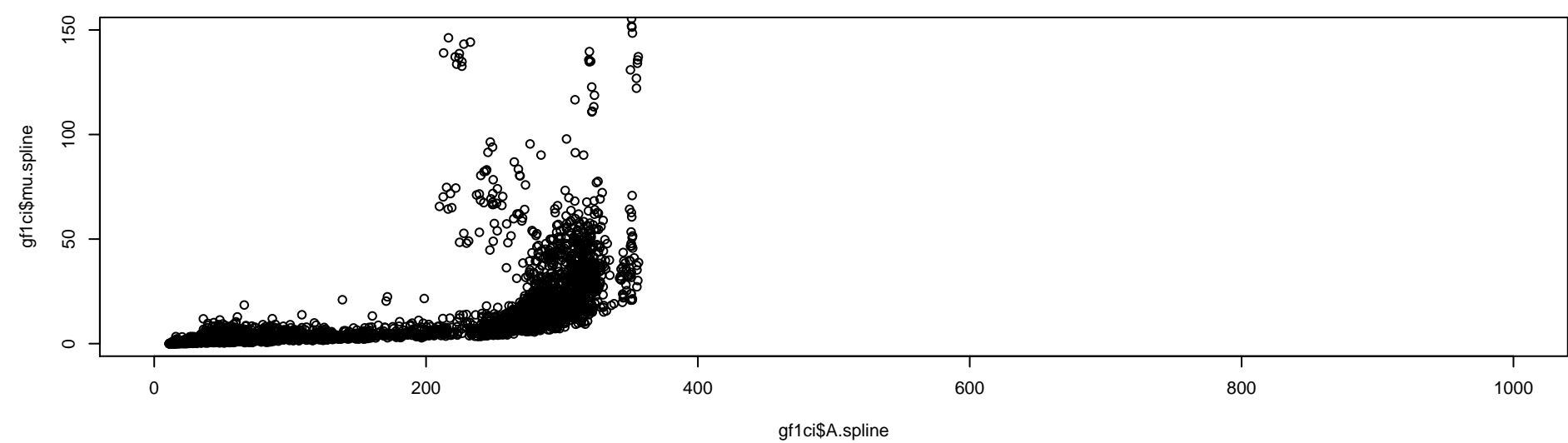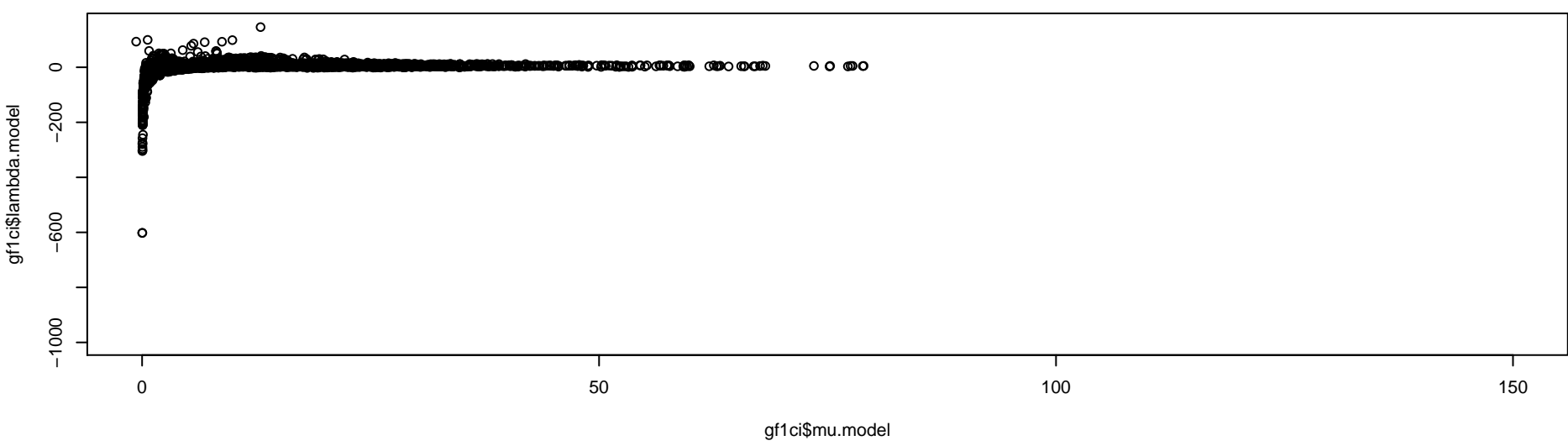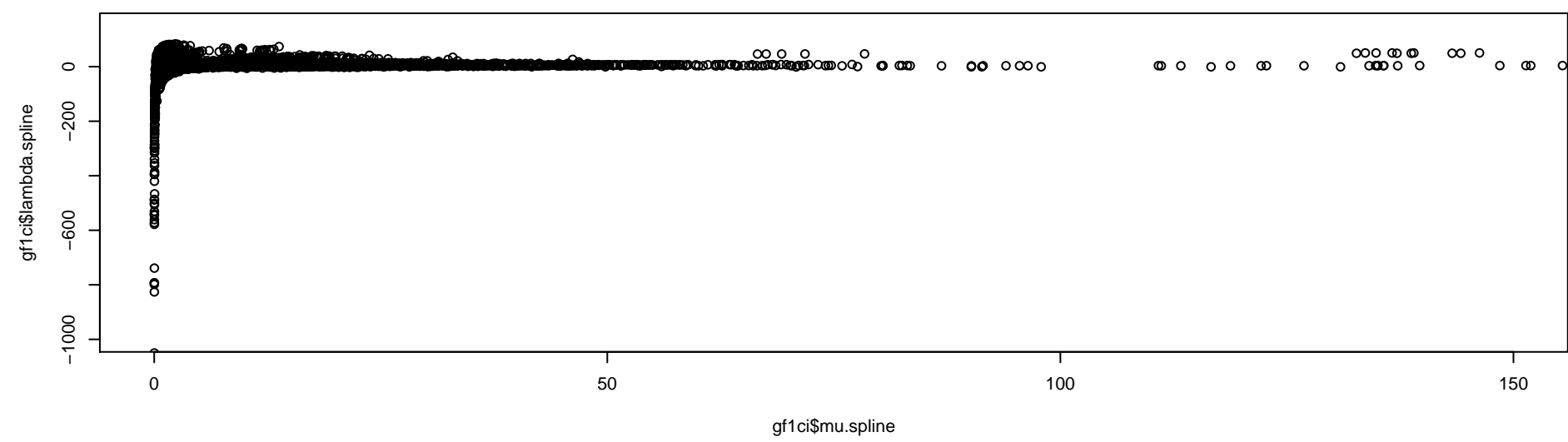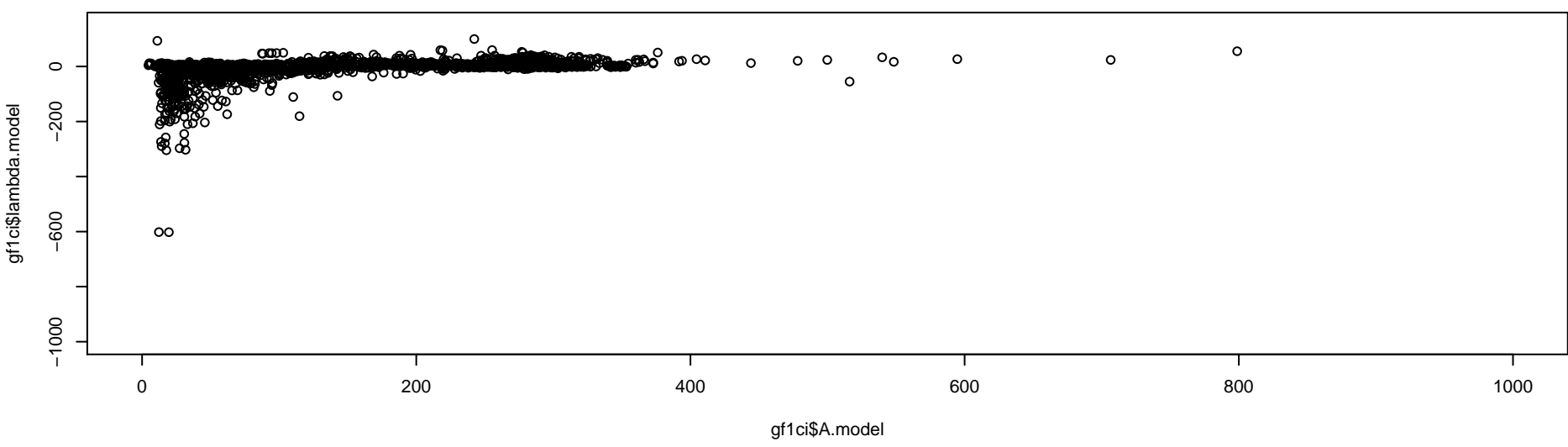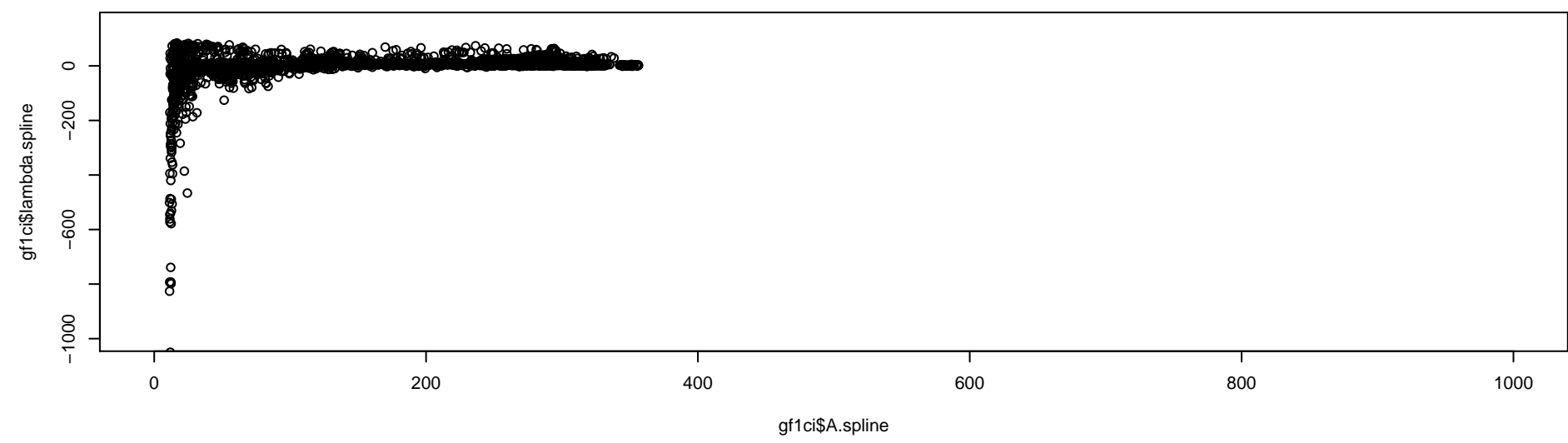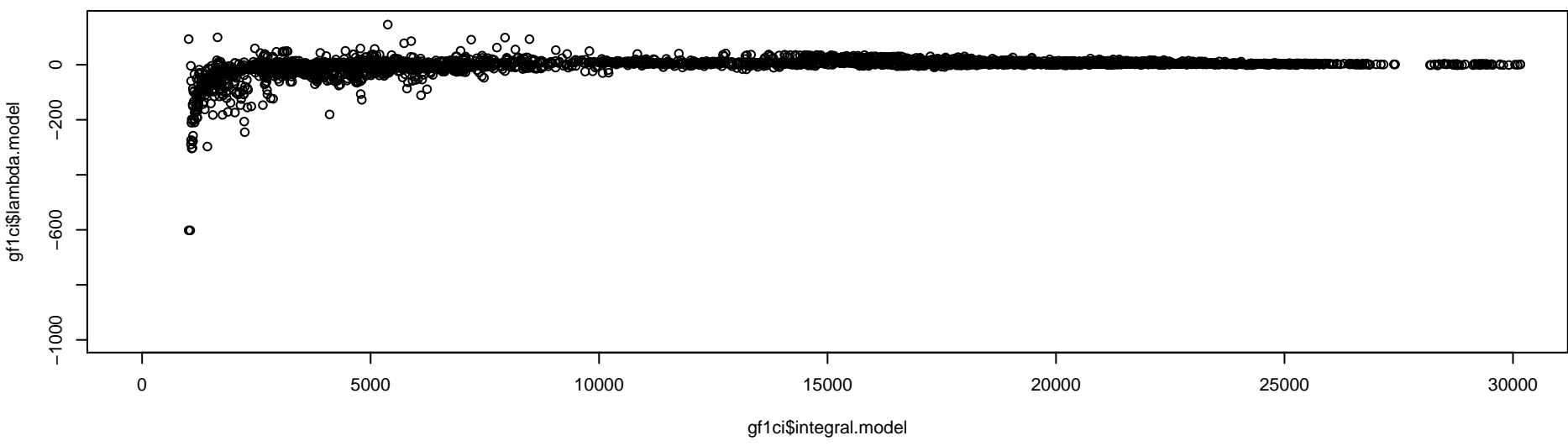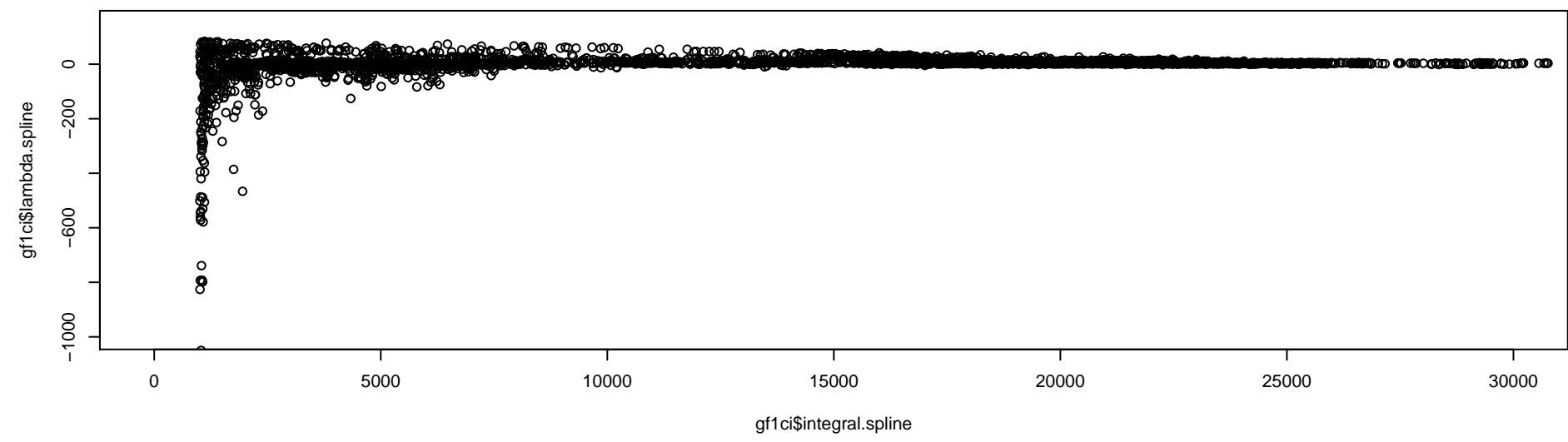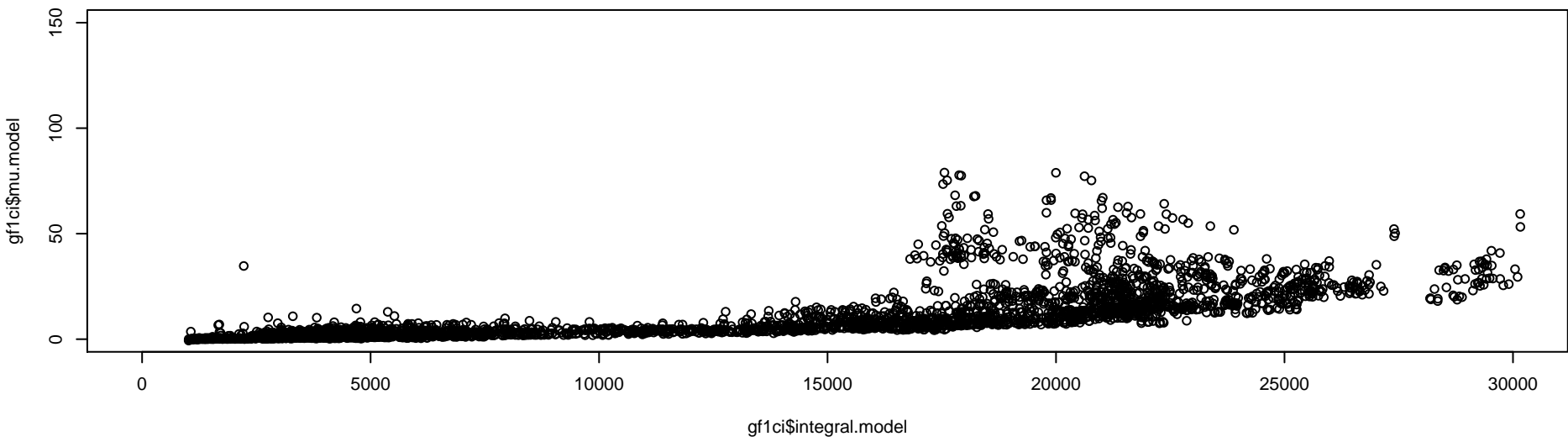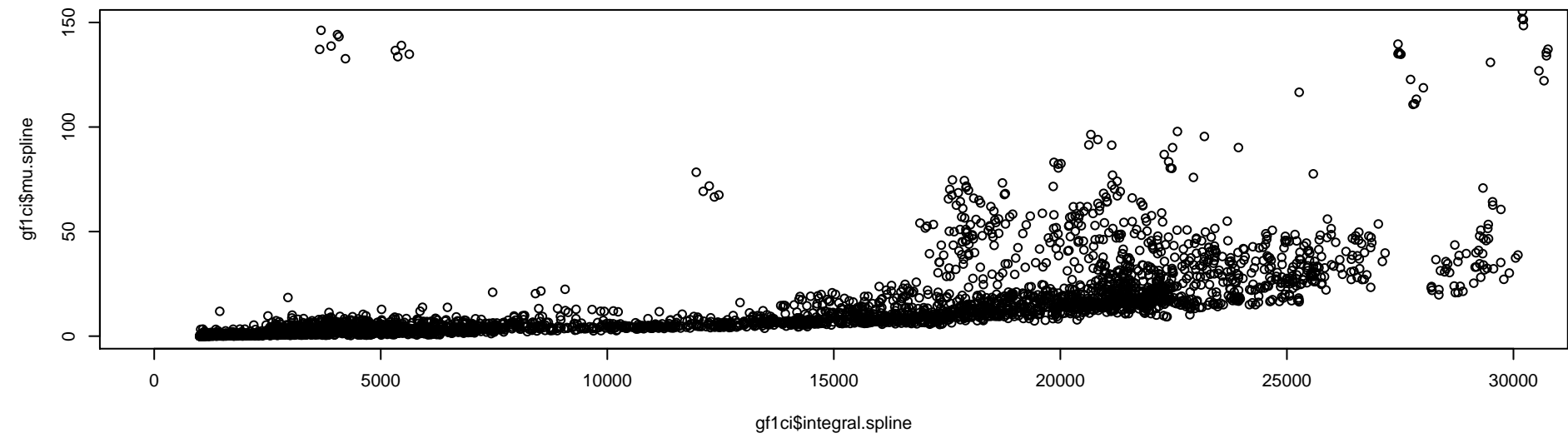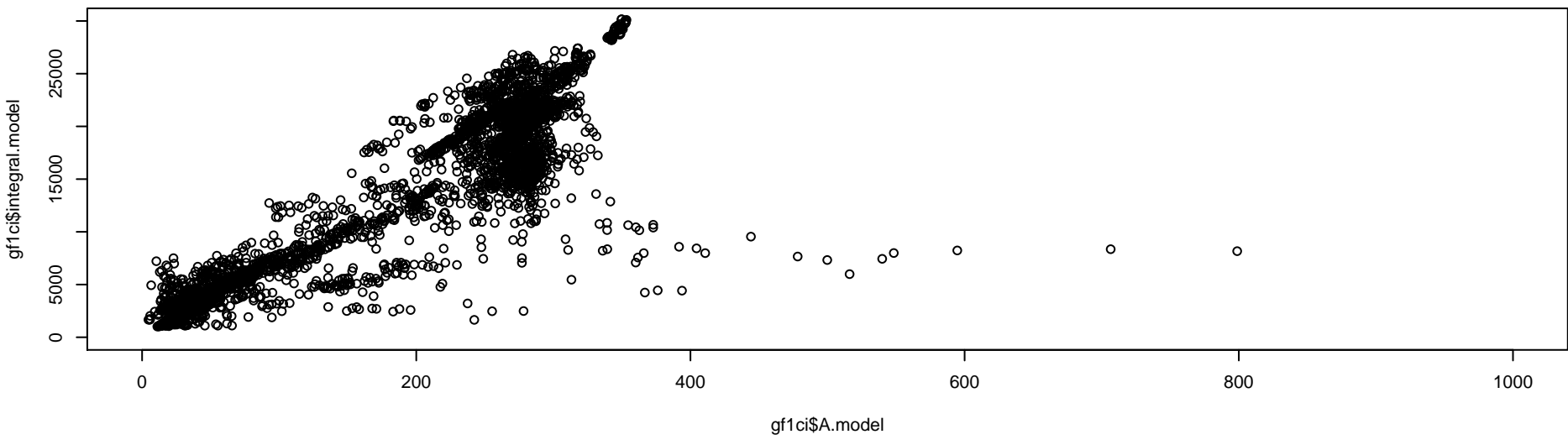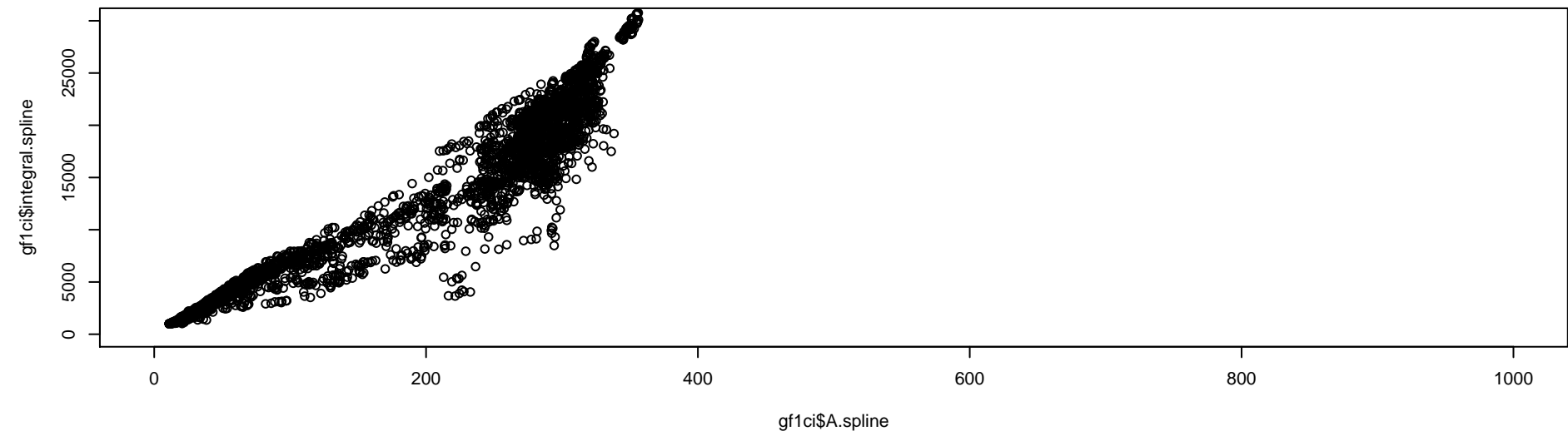

Supplement: File S10 — All-against-all correlation plots of the parameter estimates. (PDF) [file pone.0034846.s010.pdf]
