# Supplementary material for: Visualization and Curve-Parameter Estimation Strategies for Efficient Exploration of Phenotype Microarray Kinetics
Source: PLoS One. 2012 Apr 20;7(4):e34846. doi: 10.1371/journal.pone.0034846 (PMC3334903; doi:10.1371/journal.pone.0034846)
Supplement: File S9 — Behavior of the negative controls compared to negative reactions in other wells. (PDF) [file pone.0034846.s009.pdf]

Supplementary File S9 to Vaas et al., “Exploiting the wealth of Phenotype MicroArray data: efficacious visualization of and robust parameter estimation from respiration kinetics”

Behavior of the negative controls compared to negative reactions in other wells

As mentioned in the main text, it is frequently recommended to subtract the measurements from the well A01 (a negative-control well without any substrate) from those of the measurements from each other well *before* inferring curve parameters. We here (1) briefly recapture what we regard as the main underlying assumption of this recommendation and (2) discuss it in the light of some empirical observations with the datasets analyzed in the main manuscript. The fact that subtraction cannot that easily be applied to the inferred parameters themselves has been highlighted there.

1. Background assumptions when subtracting the negative control prior to parameter estimation

The recommended procedure is to subtract the, hopefully low, A01 curve from all other curves before pursuing with data analysis. This strategy assumes a biologically sensible additivity between the negative control and respiration reactions caused by the substrates. This additivity can be formally expressed as follows. Let $v_{ij} \geq 0$ be the measured value in the ith well at the jth time point, $s_{ij} \geq 0$ be the hypothetical value cleaned from background noise at these coordinates, $e_{ij} \geq 0$ the hypothetical background noise in this position, and n be the position of the negative-control well. Apparently we have

$$s_{ij} = v_{ij} - e_{ij}.$$

The suggestion to subtract the values in the negative-control well implies that

$$e_{ij} \approx v_{nj},$$

and, hence,

$$s_{ij} \approx v_{ij} - v_{nj}.$$

Due to the non-negativity constraint of all s_{ij} this implies that

$$v_{ij} \geq v_{nj}.$$

2. Empirical behavior of the negative controls in our datasets

Figure S9-1 compares the shapes of the negative-control curves between the four tested strains. Apparently the behavior of well A01 is strain-specific. *E. coli* DSM 18039 and the two *Pseudomonas* strains display a typical negative reaction, whereas the type strain of *E. coli* shows a more growth-like curve-shape (even though the maximum height is still low compared to the unambiguously positive reactions on the same plate).

Figure S9-2 confirms for *E. coli* DSM 30083^T that this typical shape of the negative-control curve occurs also throughout the 2nd biological replication. Moreover, we selected a well with a typical negative reaction, D03 (D-Arabitol), and compared it to the negative control. All D03 curves appeared shallower than the negative control.

In order to statistically confirm this observation, we compared the parameter values for maximum height (A) from the negative control well A01 with that from well D03. For each dataset, a single

one-sided t-test (test on decrease) with a confidence level of 95% was calculated, resulting in two statistically detectable group mean differences, $p = 6{,}241e^{-13}$ for dataset 1 and $p = 5{,}622^{e-10}$ for dataset 2. That is, D03 is significantly shallower than A01.

These empirical results are in sharp contrast to the above outlined theoretical assumptions which need to be fulfilled for subtracting the A01 values from the values of the other wells. They are therefore also in sharp contrast to the assumption of a biologically sensible additivity between the negative control and respiration reactions caused by the substrates. Rather, the negative control might display a reproducible, strain-specific growth-like behavior. This makes it impossible to regard it as an approximation of an error term to be subtracted from the measurements from each other well.

In such a situation, the choice a suitable strategy depends on the interpretation of the growth-like behavior in well A01. One could either discard these results as due to a not yet sufficiently optimized treatment of the strain under consideration and try to modify the pretreatment and/or the composition of the incubation medium until the curves in A01 become shallower. Alternatively, the plates could be used a such, but when dichotomizing them into positive and negative reactions, a threshold would need to be chosen that yielded negative reactions in A01. All in all, our observations (see main manuscript) strongly argue for this 2nd alternative.

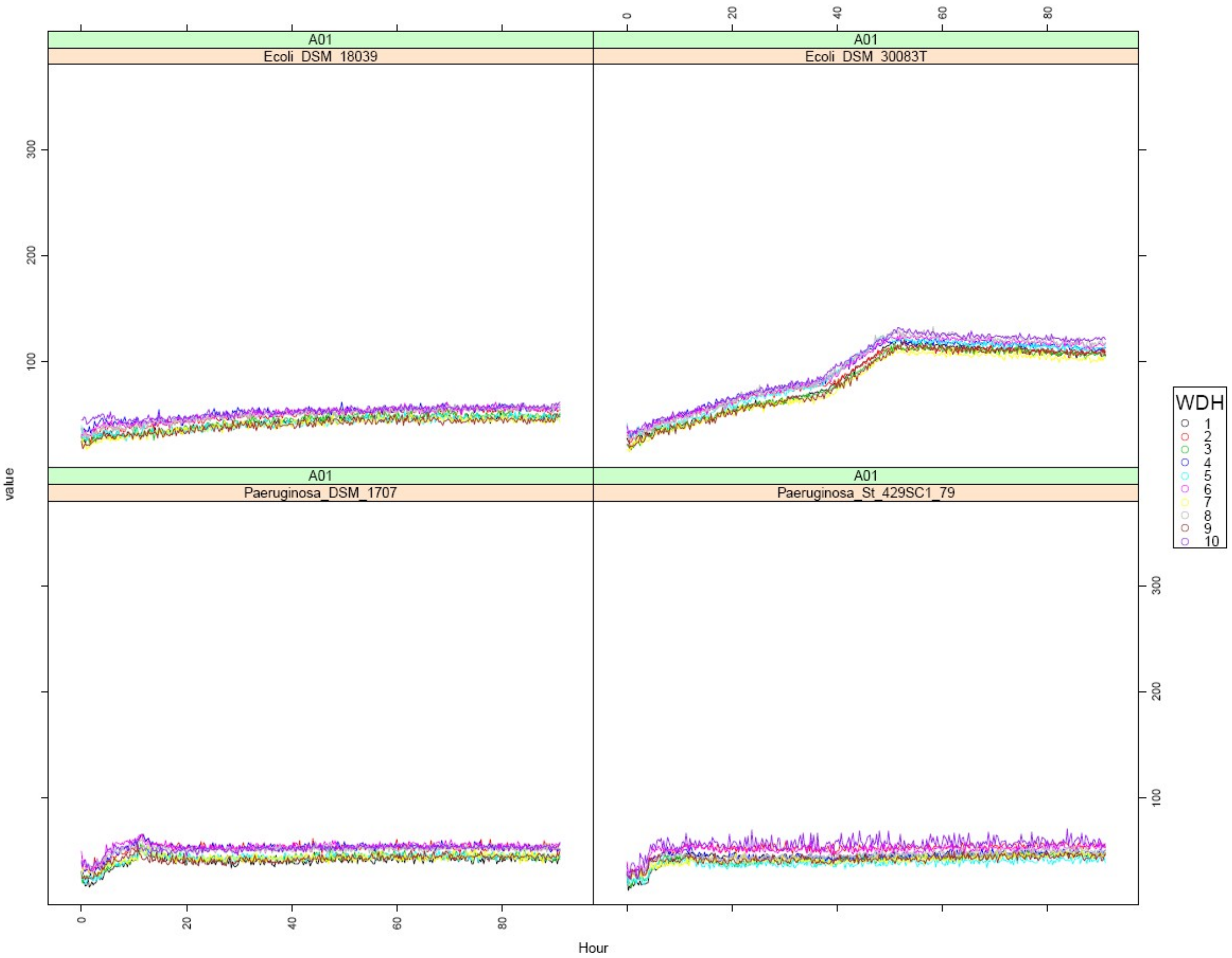

Figure S9-1. Visualization of PM curves as such *via* the function *xyplot()*. PM curves from all ten technical repetition (WDH) from the first biological replication for the four tested strains on the well A01 (negative control) arranged according a 2×4 panel layout. In the caption of each panel the corresponding the strain name is shown. The x-axes show the measurement times in hours, the y-axes the curve heights in OmniLog® units.

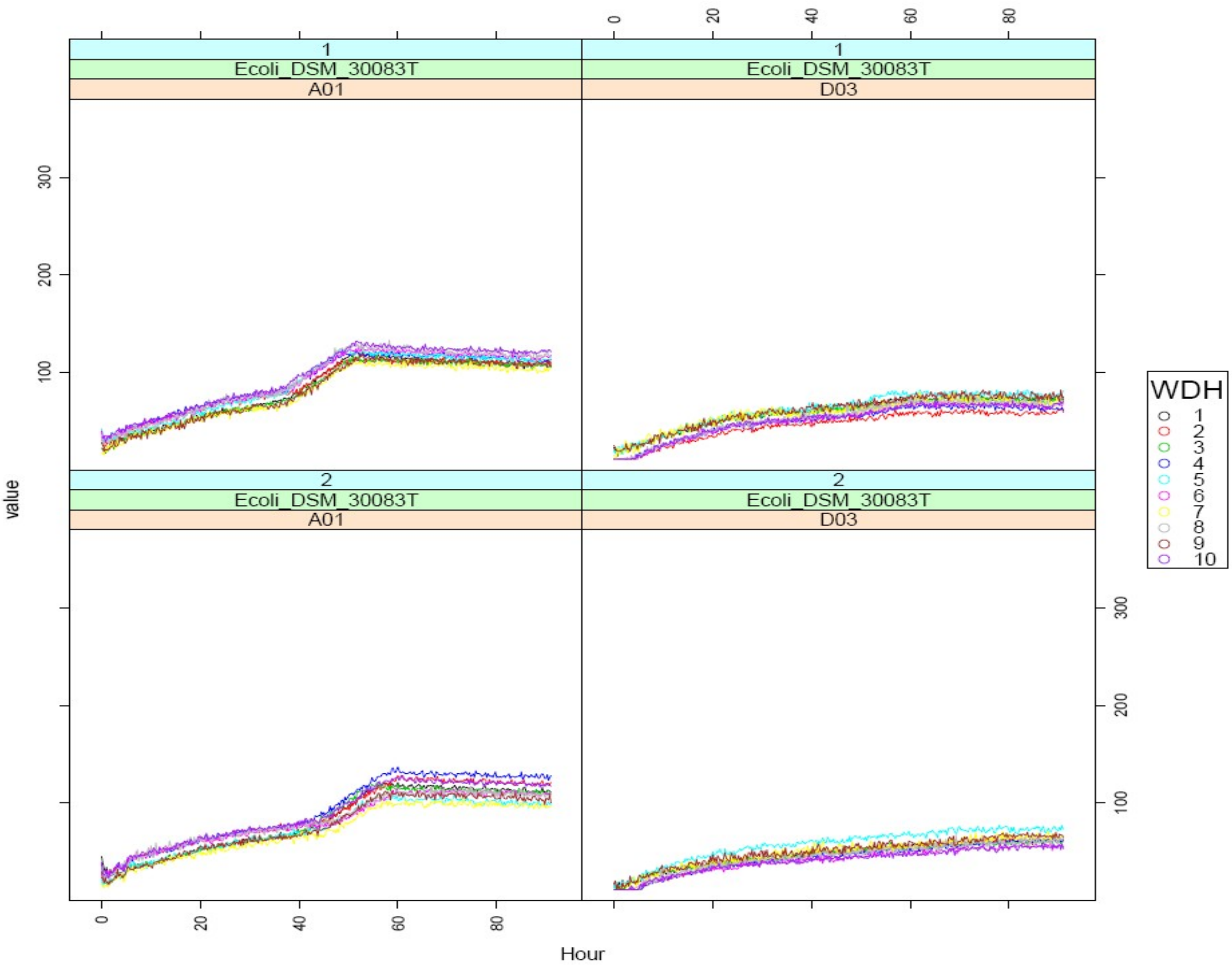

Figure S9-2. Visualization of PM curves as such *via* the function *xyplot()*. PM curves from all ten technical repetition from both datasets for the type strain of *E. coli* on the wells A01 (negative control) and D03 (D-Arabitol) were arranged according to a 2×4 panel layout. In the caption of each panel the corresponding dataset (biological replication 1 or 2), the coordinate of the well (A01 or D03) and the strain name is shown. The x-axes show the measurement times in hours, the y-axes the curve heights in OmniLog® units.
